# A chemical bactericide dioctyldiethylenetriamine (Xinjunan) exerts a non-lethal effect by inhibiting RpfG activity to regulate the quorum sensing system

**DOI:** 10.1371/journal.ppat.1014320

**Published:** 2026-06-10

**Authors:** Ling Jin, Xing Chen, Chaoyue Pang, Yongquan Huang, Changshun Ji, Yuanyuan Zhang, Xueqiao Liu, Yang Sun, Yu Chen

**Affiliations:** 1 School of Plant Protection, Anhui Agricultural University, Hefei, China; 2 Key Laboratory of Agri-products Quality and Biosafety (Anhui Agricultural University), Ministry of Education, Hefei, China; 3 Anhui Province Engineering Laboratory for Green Pesticide Development and Application, School of Plant Protection, Anhui Agricultural University, Hefei, China; 4 Key Laboratory of Integrated Crop Pest Management of Anhui Province, School of Plant Protection, Anhui Agricultural University, Hefei, China; University of Florida Institute of Food and Agricultural Sciences, UNITED STATES OF AMERICA

## Abstract

Bacterial diseases pose a major threat to global agriculture and food security. Bactericides are highly effective in disease control due to their direct antibacterial effect. However, it causes “life or death” selection pressure on the target bacteria and can lead to bactericide resistance. Quorum sensing inhibitors exert indirect antibacterial effect that weakens their virulence and combined use of bactericides and quorum sensing inhibitors demonstrated more significant disease control than a single agent. In this study, dioctyldiethylenetriamine, a bactericide that has previously been proven to have a direct antibacterial effect, exhibited an indirect antibacterial effect that weakened bacterial virulence. Using transposon sequencing, we found that the quorum sensing system, which is closely related to virulence in *Xanthomonas oryzae* pv. *oryzae* (*Xoo*, PXO99A), was significantly affected by dioctyldiethylenetriamine. It was further found that RpfG, which is responsible for transduction of the quorum sensing signal molecules in PXO99A, was significantly inhibited at both transcriptional and translational levels after dioctyldiethylenetriamine treatment, resulting in impaired phosphodiesterase activity, which in turn caused bacterial quorum quenching (QQ). QQ not only weakened the virulence of the bacteria, but also reduced their motility, which could effectively prevent the bacteria from escaping the direct antibacterial effect of dioctyldiethylenetriamine, thus providing a more effective control effect. Finally, we found that the QQ induced by dioctyldiethylenetriamine appeared to be specific, occurring in a subset of bacteria containing RpfG. The interspecific competitiveness within the same ecological niche also appeared to be weakened, which was accompanied with QQ. These findings have enriched our understanding of the antibacterial mechanism of bactericides and provided a theoretical basis for the scientific and rational use of bactericides as well as the development of “RpfG-based” indirect antibacterial agents with both efficient antibacterial activity and ecological safety.

## Author summary

Bactericides and quorum sensing inhibitors (QSIs) are effective in controlling bacterial disease due to the direct or indirect antimicrobial effects, respectively. In this study, dioctyldiethylenetriamine, a bactericide that has previously been proven to have direct antibacterial effect, exhibited indirect antibacterial effect. It caused bacterial quorum quenching (QQ) by inhibiting RpfG, which is responsible for transducing quorum sensing signal molecules in *Xanthomonas oryzae* pv. *oryzae*. QQ not only weakened the virulence of the bacteria, but also enhanced the direct antibacterial effect of dioctyldiethylenetriamine. Further, we found that the QQ induced by dioctyldiethylenetriamine appeared to be specific, occurring in a subset of bacteria containing RpfG. Bacterial interspecific competitiveness also appeared to be weakened accompanied with QQ. These findings not only enriched our understanding of the antibacterial mechanism of bactericides, but also provided a theoretical basis for the development of “RpfG-based” indirect antibacterial agents with both efficient antibacterial activity and ecological safety.

## Introduction

Plant diseases caused by pathogens pose a severe threat to the sustainability of global agriculture and food security [1]. Of these, bacteria have surpassed viruses as the second most damaging pathogen to crops, after fungi, and bacterial disease outbreaks can result in widespread crop yield reductions [2]. Among all the bacterial disease management strategies, application of bactericides is the most common, economical and effective management method [1]. Bactericides used for the control of plant bacterial diseases are categorized into copper-based pesticides, zinc-based pesticides, antibiotics and others, depending on the active ingredient contained [3]. The mechanism of bactericides usually includes disruption of the integrity of bacterial cell walls, interference with bacterial protein synthesis and inhibition of the bacterial energy synthesis pathway. For example, copper-based pesticides [4,5] and zinc thiazole [3] can disrupt the integrity of bacterial cell wall by releasing copper ions to inhibit the maturation of lipoproteins, a key component of the bacterial cell envelope, or by mediating cell wall disintegration with the aid of thiazole structures, respectively. Streptomycin [6] and zhongshengmycin [3] can respectively target the 30S ribosomal protein RpsL or hinder the translation of bacterial proteins at the RNA level, thereby interfering with the synthesis of bacterial proteins. Berberine [7], a plant-based bactericide, can affect the energy synthesis of bacteria by interfering with bacterial energy metabolism and respiration. Bactericides have shown high efficacy in disease control due to their direct antibacterial effect of killing bacteria. However, their direct antibacterial effect causes a “life or death” selection pressure on the target bacteria, and their long-term use may lead to the development of bacterial resistance [8]. This situation has prompted researchers to explore new agents to overcome the limitations of existing bactericides.

In recent years, indirect antibacterial methods that weaken the virulence of bacteria or induce plant immune responses have become one of the important strategies for the management of bacterial diseases [9–11]. Among them, quorum sensing inhibitors (QSIs) may exert relatively milder evolutionary pressure on the development of bacterial resistance by interfering with the bacteria’s quorum sensing (QS) and adopting an alternative strategy of inhibiting bacterial virulence rather than growth [9,12]. This strategy is not only effective in disease control but also helps to alleviate the emergence of bacterial resistance, providing a new direction for efficient crop protection. QS is a unique intercellular communication mechanism in which bacteria can sense the density of populations by secreting signaling molecules known as autoinducers (AIs) [13], and regulates gene expression through a series of cascading responses to coordinate population behavior, such as swimming, swarming, biofilm formation and virulence factor expression [14,15]. In the prevention and control of diseases, quercetin [16], curcumin [17], vanillin [18], and furanone [19] have been proven to cause quorum quenching (QQ) of bacteria by inhibiting the synthesis of QS signal molecules, blocking the binding of signal molecules to receptor proteins or degrading signal molecules, thus significantly reducing the virulence of pathogens [8]. These compounds play an important role in the prevention and control of pathogenic bacteria such as *Pseudomonas aeruginosa* (*Pa*) [20], *Staphylococcus aureus* (*Sa*) [20], and *Vibrio harveyi* [19].

It is worth noting that the combination of a QSI quercetin and copper nanoparticles with direct antibacterial effect not only effectively inhibited the biofilm formation of *Pa* and *Sa*, but also reduced inflammatory responses and accelerated the healing of bacteria-infected wounds in the host body [20]. In addition, the combination of sulfonamides, silver antimicrobial agents and QSIs has increased the toxicity against *Bacillus subtilis* (*Bs*) while reducing the use of antimicrobial agents [21]. In contrast, the combined use of *N*-(2-pyrimidyl) butanamide (a type of QSI) and antibiotics not only improved the efficacy against *Pa*, but also alleviated the development of bacterial resistance to antibiotics [22]. The combined use of agents with different modes of action showed more significant control effects than the individual use and helped to alleviate the emergence of bacterial resistance. This provides new strategies and ideas for integrated disease prevention and control. Recent studies have revealed that the plant-derived agent allicin has both direct and indirect antibacterial effects by inhibiting bacterial growth and interfering with the QS system [23,24]. However, the discovery that an agent has both direct and indirect dual antibacterial effects is relatively rare. In addition, the molecular mechanism by which the agent exerts a dual antibacterial effect is currently unknown.

Dioctyldiethylenetriamine (IUPAC name: N^1^-octyl-N^2^-[2-(octylamino)ethyl] ethane-1,2-dia-mine, CAS No. 57413-95-3, also known as Xinjunan in China) is an alkyl polyamine bactericide [25], that has been registered and widely used in China for disease control in a wide range of crops, including apple, tomato, cotton, and rice, since it was first reported to be used in the control of agricultural diseases in 1990 [2,25]. Its high efficacy and broad-spectrum antibacterial activity showed significant inhibitory effect on a variety of plant pathogenic bacteria, such as *Xanthomonas oryzae* pv. *oryzae* (*Xoo*), *Pseudomonas syringae* pv. *tomato*, *Acidovorax citrulli* (*Ac*), and *Clavibacter michiganensis* subsp. *michiganensis* [2]. The safety evaluation showed that dioctyldiethylenetriamine exhibited environmentally friendly characteristics of low residue [26] and no toxicity to non-target organisms [25,27]. These characteristics make dioctyldiethylenetriamine a powerful “weapon” against a wide range of bacterial plant diseases. Although dioctyldiethylenetriamine plays an important role in the field disease control, the molecular mechanism of its antibacterial action is still in the preliminary stage. The present research results show that dioctyldiethylenetriamine exerts its direct antibacterial effect through at least two pathways. One is to block energy synthesis by inhibiting the tricarboxylic acid cycle and oxidative phosphorylation process in bacteria, leading to bacterial death due to energy exhaustion [2]. Second, it disrupts intracellular iron homeostasis, leading to the entry and accumulation of large amounts of Fe^3+^ intracellularly, which triggers the Fenton reaction to produce hydroxyl radicals with high activity, disruption of DNA synthesis and bacterial death [25]. It is noteworthy that although dioctyldiethylenetriamine has been used for more than 30 years, no resistant strains have been reported in the field to date.

To clarify whether there is an indirect antibacterial effect in addition to the direct antibacterial effect of dioctyldiethylenetriamine, we selected the *Xoo*, whose growth was significantly inhibited by dioctyldiethylenetriamine, as a model strain and studied it with the transposon sequencing technology. The results of the transposon sequencing data suggested that dioctyldiethylenetriamine may have a regulatory effect on the QS of *Xoo*. Transcriptional, genetic, and physiological-biochemical evidence was provided to demonstrate that dioctyldiethylenetriamine can inhibit the QS of bacteria and thereby reduce bacterial virulence. Further studies revealed that inhibition of the QS by dioctyldiethylenetriamine enhanced its direct antibacterial effect. Notably, this inhibition phenomenon is specific and occurs in a subset of bacteria containing the RpfG. Moreover, *Xoo* with impaired QS exhibited a reduced ecological niche in interspecific competition among bacteria. This discovery not only reveals a new anti-bacterial mechanism of dioctyldiethylenetriamine, which provides a new perspective for the study of the mechanism of bactericides, but also provides a theoretical basis for the development of new types of bactericides with multiple mechanisms of action.

## Results

### QS system of *Xoo* in response to the bactericide dioctyldiethylenetriamine

**Fig 1:**
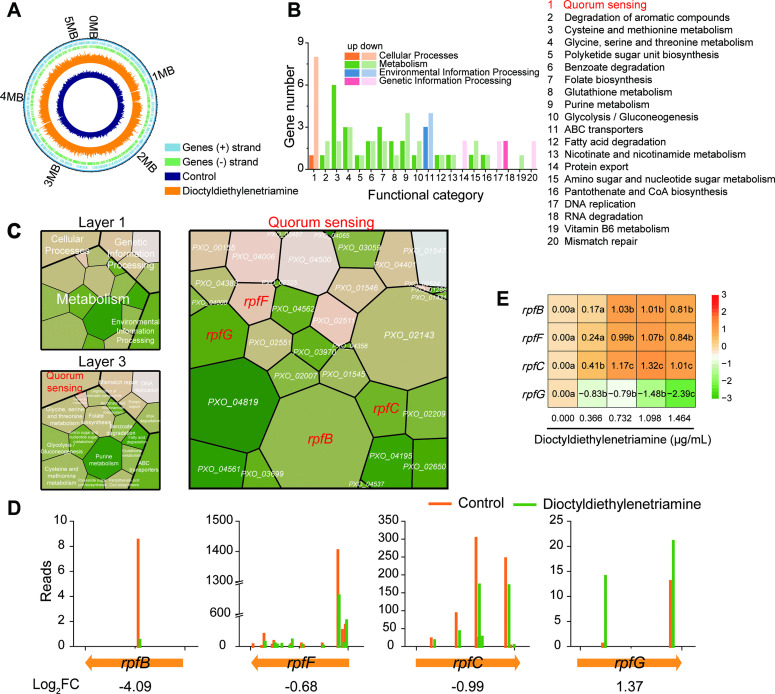
QS system of *Xoo* in response to the dioctyldiethylenetriamine. **(A)** Circular plot showing the transposon insertion abundance along the *Xoo* strain PXO99A genome under different treatment conditions. The two outer rings depict genes located on the (+) or (−) strand in PXO99A strain CP000967.2. The two inner rings show the histograms of transposon insertions on a per gene basis after growth of the library in dioctyldiethylenetriamine (orange) and control (blue) condition for 24 **h. (B)** Functional categories of differential genes identified by KEGG analysis. Details were listed in S7 Table. **(C)** Voronoi diagram-based visualization of PXO99A genes when exposed to dioctyldiethylenetriamine underlines differences (Only the top 20 differential gene function categories identified based on KEGG analysis were shown.). The colors of the polygons in the left figures indicate functional categories. Colors of polygons in right figure indicate genes. The larger the polygon area, the greater the change in transposon insertion abundance under dioctyldiethylenetriamine condition compared to control condition. **(D)** Distribution of transposon insertions in the *rpfB*, *rpfF*, *rpfC*, and *rpfG* genes in the transposon insertion libraries with and without dioctyldiethylenetriamine. The arrow length represents the corresponding gene length, and the direction of the arrow indicate the transcription direction of the gene. Log_2_FC represents the log-fold-change of transposon insertion abundance between treated (dioctyldiethylenetriamine) and untreated (control) groups. **(E)** Expression levels of *rpfB*, *rpfF*, *rpfC*, and *rpfG* genes in PXO99A strain after dioctyldiethylenetriamine treatment. Sample size *n* = 3. Numbers on the heatmap indicating relative gene expression levels were calculated using the log_2_2^-ΔΔCT^ method. The results of the same gene under different treatments were analyzed using one-way ANOVA followed by Tukey’s multiple range test, with different letters next to the numbers indicate statistically significant difference at *P* < 0.05.

To investigate whether there was an indirect antibacterial effect in addition to the direct antibacterial effect of dioctyldiethylenetriamine, we selected PXO99A (a representative strain of *Xoo*), whose growth was significantly inhibited by dioctyldiethylenetriamine, as a model strain. The effective concentration of 50% inhibition (EC_50_) of dioctyldiethylenetriamine against PXO99A was 0.366 μg/mL [2]. Following transposon mutant library of PXO99A previously established in our laboratory [28] was treated with dioctyldiethylenetriamine, transposon sequencing (Tn-seq, a high-throughput assay that can be used to identify genes that are critical for bacterial survival under specific selective pressures or to find potential target genes for compounds) was performed to investigate the mechanism of action of dioctyldiethylenetriamine (Fig 1A). A total of 315 differential genes were obtained based on the changes in transposon insertion abundance of each gene after dioctyldiethylenetriamine treatment (with |log_2_FC| ≥ 1 as the threshold, where log_2_FC represents the log-fold-change of transposon insertion abundance between treated and untreated groups). Of these, the transposon insertion abundance of 163 genes increased (log_2_FC > 1, potential drug target-related genes) and that of 152 genes decreased (log_2_FC < 1, potential drug resistance-related genes) (S1 Fig). Kyoto Encyclopedia of Genes and Genomes (KEGG) analysis showed that the differential genes were significantly enriched in the QS system (Fig 1B). The Voronoi diagram was created based on the significance of the differential genes (|log_2_FC| value), and the results showed that more differential genes were enriched in the QS system (Fig 1B), and the change of transposon insertion abundance of the system was significantly greater than that of other pathways (Fig 1C).

The QS system is a communication mechanism between bacterial cells that coordinates population behavior through the production of signaling molecules, including motility and virulence factors, which are essential for bacterial virulence. The signaling molecule of the QS system of *Xanthomonas* spp. is the diffusion signal factor (DSF), the processing, biosynthesis, sensing and transduction of which is regulated by a cluster of “regulation of pathogenicity factors” (*rpf*) genes [29]. Tn-seq results revealed that, in addition to *rpfB*, *rpfF*, *rpfC*, and *rpfG* within the QS system, the transposon insertion abundance of genes such as *PXO_04819* and *PXO_02143* also underwent significant changes before and after treatment with dioctyldiethylenetriamine (S1 Table). Although these genes belong to the QS system, they were not part of the *rpf* gene cluster and their functions were regulated by the *rpf* gene cluster. Considering that the *rpf* gene cluster was the core regulatory element of the QS system of *Xanthomonas* spp., we further analyzed the changes in the transposon insertion abundance of *rpfB*, *rpfF*, *rpfC*, and *rpfG* in the *rpf* gene cluster after treatment with dioctyldiethylenetriamine. We found that the abundance of transposon insertions in the *rpfB*, *rpfF*, and *rpfC* was reduced while *rpfG* was increased, with log_2_FC values of -4.09, -0.68, -0.99, and 1.37, respectively (Fig 1D). Additionally, the PXO99A strain in the mid-exponential phase was cultivated in 0.9% NaCl solution and the OD_600_ was normalized to 0.4, and it was then treated with different concentrations of dioctyldiethylenetriamine that did not inhibit bacterial growth under these conditions (S2 Fig). The qRT-PCR results showed that the expression levels of *rpfB*, *rpfF*, and *rpfC* were slightly up-regulated while that of *rpfG* was significantly down-regulated (Fig 1E). These suggested that the *rpfB*, *rpfF*, and *rpfC* might play an active role in the adaptation of bacteria to the dioctyldiethylenetriamine environment and the *rpfG* might be the target gene. It has been shown that deletion of *rpfB*, *rpfF*, and *rpfC* leads to changes in DSF content, whereas deletion of *rpfG* hinders the downstream transduction of QS signals and causes QQ in bacteria, although it does not affect DSF content [30–35]. Here, we found that the content of DSF did not change significantly after dioctyldiethylenetriamine treatment (S3 Fig). Therefore, we shifted the focus of our study to the effect of dioctyldiethylenetriamine on RpfG function to understand whether dioctyldiethylenetriamine could cause QQ by inhibiting RpfG activity.

### Dioctyldiethylenetriamine induces QQ of PXO99A by inhibiting the RpfG

**Fig 2:**
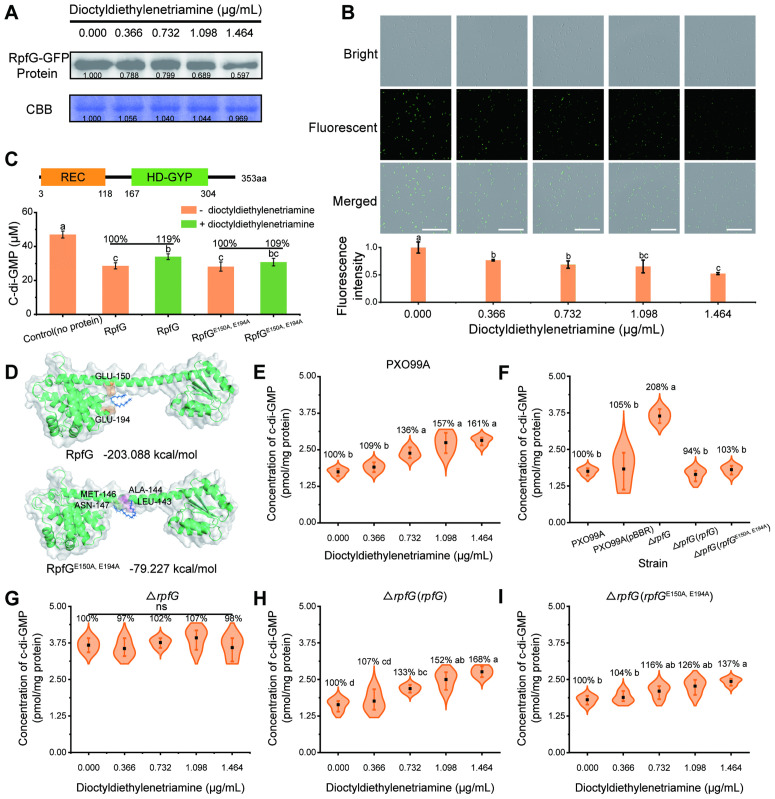
Evidence suggests that dioctyldiethylenetriamine inhibits RpfG activity. **(A)** Effect of different concentrations of dioctyldiethylenetriamine on the expression of RpfG-GFP protein in strain rpfG-GFP-PXO99A. The band intensities were quantified and analyzed using ImageJ, with numbers representing the relative intensities of the corresponding bands. Coomassie brilliant blue (CBB) staining was employed as a loading control to verify the equal amounts of protein across the gel. **(B)** Changes in fluorescent intensity of strain rpfG-GFP-PXO99A after treatment with different concentrations of dioctyldiethylenetriamine. Photographs were taken at 488 nm using a scanning confocal laser microscopy. Scale bar = 25 μm. The intensity of fluorescence was analyzed using ImageJ software. **(C)** Phosphodiesterase (PDE) activity assay of RpfG *in vitro*. Top: Schematic diagram of RpfG. The predicted domains are in the boxes, and the amino acid numbers are at the bottom based on the protein families (Pfam) database. Bottom: PDE activity assay of RpfG *in vitro*. The reaction system without additional protein was used as the negative control. The concentrations of dioctyldiethylenetriamine in the treatment group were either 0 or 1.464 μg/mL. **(D)** The binding mode of dioctyldiethylenetriamine between RpfG and RpfG^E150A, E194A^. **(E)** Detection of intracellular c-di-GMP content in PXO99A strain after dioctyldiethylenetriamine treatment. **(F)** Detection of intracellular c-di-GMP content in different bacterial strains. **(G)** Detection of intracellular c-di-GMP content in Δ*rpfG* strain after dioctyldiethylenetriamine treatment. **(H)** Detection of intracellular c-di-GMP content in *rpfG* genetic complementary strains (Δ*rpfG*(*rpfG*)) strain after dioctyldiethylenetriamine treatment. **(I)** Detection of intracellular c-di-GMP content in Δ*rpfG*(*rpfG*^E150A, E194A^) strain after dioctyldiethylenetriamine treatment. Results shown in (A) were obtained independently in two experiments and (B, C, E, F, G, H, I) in three experiments. Both the bar graphs and violin plots denote mean ± SD. Error bars indicate SD. Results were analyzed using one-way ANOVA followed by Tukey’s multiple range test, with different letters above the figures indicate statistically significant difference at *P* < 0.05, “ns” stands for not statistically significant. The percentage values in panels (C, E, F, G, H, I) represent the ratio of c-di-GMP content in either the dioctyldiethylenetriamine-treated group relative to the untreated control group or the mutant strains relative to the wild-type strain PXO99A.

To determine whether dioctyldiethylenetriamine affected RpfG function, we first determined whether dioctyldiethylenetriamine inhibited RpfG translation. We constructed the reporter strain rpfG-GFP-PXO99A and the control strain GFP-PXO99A to monitor RpfG translation. The rpfG-GFP-PXO99A and GFP-PXO99A strains were treated with dioctyldiethylenetriamine at different concentrations that did not inhibit bacterial growth (S2 Fig), and the crude proteins of the treated strains were extracted and analyzed for the content of RpfG-GFP and GFP by western blotting. The results showed that the RpfG content of rpfG-GFP-PXO99A was significantly reduced by treatment with dioctyldiethylenetriamine, and within the tested concentrations, the inhibitory effect was improved with the increasing concentration of dioctyldiethylenetriamine (Fig 2A). However, the content of GFP in the GFP-PXO99A strain was not affected by dioctyldiethylenetriamine (S4 Fig). Subsequently, the fluorescence intensity of RpfG-GFP and GFP were observed using laser confocal microscopy. We found that the fluorescence intensity of the rpfG-GFP-PXO99A strain decreased with the increasing concentration of dioctyldiethylenetriamine (Fig 2B), while that of the GFP-PXO99A strain showed no significant change (S4 Fig). The results obtained by laser confocal microscopy observation were consistent with those of western blotting, further demonstrating the inhibitory effect of dioctyldiethylenetriamine on RpfG. These results indicated that dioctyldiethylenetriamine not only inhibited the transcription but also the translation of *rpfG*.

Next, we tested whether dioctyldiethylenetriamine affected RpfG activity. RpfG contains the HD-GYP structural domain, which has been shown to have the ability to hydrolyze cyclic dimeric GMP (c-di-GMP) in *Lysobacter enzymogenes* [36]. We purified the RpfG, and *in vitro* assays showed its activity in hydrolyzing c-di-GMP. Following the addition of dioctyldiethylenetriamine, the c-di-GMP hydrolase activity of RpfG decreased, indicating that dioctyldiethylenetriamine could inhibit RpfG activity *in vitro* (Fig 2C). The three-dimensional structure of the RpfG protein was predicted using AlphaFold 3, and the binding mode and affinity between dioctyldiethylenetriamine and RpfG were simulated using Discovery Studio software. The results indicated that the predicted binding sites between dioctyldiethylenetriamine and RpfG were glutamic acid residues (Glu, E) at positions of 150 and 194, with a predicted binding energy of -203.088 kcal/mol (lower binding energy indicates stronger affinity between the two) (Fig 2D). To verify whether dioctyldiethylenetriamine inhibiting RpfG activity was related to these two sites, we mutated glutamic acid residues at positions of 150 and 194 (Glu, E) in RpfG to alanine (Ala, A). *In vitro* phosphodiesterase activity assays revealed that the mutated RpfG^E150A, E194A^ retained PDE activity, but its activity was scarcely inhibited by dioctyldiethylenetriamine (Fig 2C). Molecular docking simulations revealed that the binding affinity between the mutated RpfG^E150A, E194A^ and dioctyldiethylenetriamine was significantly decreased, with a predicted binding energy of -79.227 kcal/mol (Fig 2D).

To further test whether dioctyldiethylenetriamine caused changes in bacterial intracellular c-di-GMP content, the PXO99A was treated by different concentrations of dioctyldiethylenetriamine that did not inhibit bacterial growth (S2 Fig). The results showed that the content of c-di-GMP increased significantly after dioctyldiethylenetriamine treatment (Fig 2E). C-di-GMP is a ubiquitous dinucleotide second messenger molecule in diverse bacteria that controls a variety of physiological processes, including changes in bacterial motility, virulence, cell cycle and differentiation via intracellular protein effectors (or receptors) and riboswitches [36]. The synthesis and hydrolysis of c-di-GMP are regulated by diguanylate cyclases (DGCs) containing the catalytic structural domain of GGDEF and phosphodiesterases (PDEs) containing the catalytic structural domains of EAL or HD-GYP, respectively [36]. To clarify whether elevation of intracellular c-di-GMP content in PXO99A which was only due to the inhibition of RpfG activity by dioctyldiethylenetriamine, we constructed *rpfG* deletion mutant (Δ*rpfG*) and complemented strain (Δ*rpfG*(*rpfG*)), and examined the changes in the intracellular c-di-GMP content of these strains after dioctyldiethylenetriamine treatment. The results showed that the content of c-di-GMP was significantly increased in the Δ*rpfG* compared to the PXO99A, but there was no significant change after dioctyldiethylenetriamine treatment (Fig 2F and 2G). The content of c-di-GMP in the Δ*rpfG*(*rpfG*) was not significantly different from that of the PXO99A, however, both Δ*rpfG*(*rpfG*) and PXO99A exhibited a comparably significant increase in c-di-GMP content after dioctyldiethylenetriamine treatment (Fig 2F and 2H). Following expression of *rpfG*^E150A, E194A^ in Δ*rpfG*, intracellular c-di-GMP levels in the Δ*rpfG*(*rpfG*^E150A, E194A^) nearly recovered to those of the PXO99A strain (Fig 2F). Although dioctyldiethylenetriamine induced changes in intracellular c-di-GMP levels in Δ*rpfG*(*rpfG*^E150A, E194A^) cells, the magnitude of these changes was significantly reduced compared to the effect observed in the PXO99A strain (Fig 2I). This indicated that dioctyldiethylenetriamine inhibited RpfG function in bacteria by affecting the glutamic acids at positions 150 and 194 of RpfG. To solidify the conclusion that the increase in intracellular c-di-GMP levels in PXO99A resulted from the specific inhibition of RpfG activity by dioctyldiethylenetriamine, we expressed the potent heterologous PDE, Yhjh (containing an EAL domain) from *Escherichia coli* in Δ*rpfG* [36]. The results showed that c-di-GMP levels in Δ*rpfG*(*yhjH*) were significantly reduced compared to Δ*rpfG*, indicating Yhjh exhibited PDE activity in Δ*rpfG*. Following dioctyldiethylenetriamine treatment, c-di-GMP levels in the Δ*rpfG*(*yhjH*) mutant remained unchanged (S5 Fig). These results suggested that dioctyldiethylenetriamine might block the downstream transduction of signaling molecules by inhibiting the activity of RpfG, leading to an abnormal accumulation of bacterial intracellular c-di-GMP content, which triggered QQ.

In *Xoo*, high intracellular levels of c-di-GMP can specifically bind to the global transcription regulator Clp, thereby inhibiting the transcriptional activation activity of Clp to modulate the expression of virulence-related genes [37]. To clarify whether the accumulation of intracellular c-di-GMP induced by dioctyldiethylenetriamine-mediated inhibition of RpfG activity is directly involved in the regulation of *rpfG* expression, we detected the interaction between Clp and the promoter region of *rpfG* using a bacterial one-hybrid assay. The results showed that Clp could specifically bind to the promoter region of *rpfG* (S6 Fig), suggesting that Clp might directly regulate the transcription of *rpfG*. Meanwhile, we used a β-Glucuronidase (GUS) reporter system to examine the effect of Clp on *rpfG* promoter activity in *Xoo*. The results showed that *rpfG* promoter activity was significantly reduced in the *clp* deletion mutant (Δ*clp*) compared with the wild-type PXO99A strain, as reflected by decreased blue color development and lower GUS activity. Furthermore, treatment with either c-di-GMP or dioctyldiethylenetriamine significantly inhibited *rpfG* promoter activity in PXO99A, but had no significant effect on the Δ*clp* mutant (S6 Fig). Further qRT-PCR assays revealed that the transcriptional level of *rpfG* in the Δ*clp* was significantly decreased compared with that in the wild-type strain PXO99A (S6 Fig). These results indicated that Clp might act as a transcriptional activator to positively regulate *rpfG* expression, and that the regulatory effect of dioctyldiethylenetriamine on *rpfG* expression was depended on Clp. To determine the effect of Clp on the translation of RpfG, we used the Δ*clp* mutant as the research object, with dioctyldiethylenetriamine-treated and untreated groups, and detected RpfG protein levels by western blot. The results showed that RpfG protein levels in the Δ*clp* mutant exhibited almost no significant change after dioctyldiethylenetriamine treatment compared with the untreated group (S6 Fig). This phenomenon was distinct from the significant reduction in RpfG protein levels induced by dioctyldiethylenetriamine in the PXO99A strain (Fig 2A–2B). These findings demonstrated that dioctyldiethylenetriamine might induces abnormal accumulation of intracellular c-di-GMP by inhibiting RpfG activity, which in turn reduced the binding efficiency of Clp to the *rpfG* promoter and inhibited the transcription and translation of *rpfG*. It was worth noting that the *in vitro* inhibitory effect of dioctyldiethylenetriamine on RpfG activity (Fig 2C) was weaker than its inhibitory effect in bacteria (Fig 2E-2I). We speculated that this difference might be attributed to the fact that the *in vitro* reaction system could not fully simulate the complex intracellular microenvironment within bacteria and lacked the Clp-mediated transcriptional regulation mechanism that was unique to the *in vivo* setting. To elucidate the differential response mechanism of key QS system genes upon dioctyldiethylenetriamine treatment, we further determined the expression levels of *rpfB*, *rpfF*, and *rpfC* in the Δ*rpfG* strain. The results indicated that the transcriptional levels of *rpfB*, *rpfF*, and *rpfC* in the ΔrpfG strain were significantly higher than those in the PXO99A strain (S7 Fig). This phenomenon was consistent with the upregulated expression levels of *rpfB*, *rpfF*, and *rpfC* in PXO99A following dioctyldiethylenetriamine treatment (Fig 1E). In the *rpfG*-overexpressing strain (OE-*rpfG*), when the expression level of *rpfG* in the dioctyldiethylenetriamine-treated OE-*rpfG* strain was not significantly different from that in the untreated wild-type strain, the expression levels of *rpfF*, *rpfB*, and *rpfC* also showed no obvious changes compared with the untreated wild-type strain (S7 Fig). These results suggested that the dioctyldiethylenetriamine-induced upregulation of *rpfB*, *rpfF*, and *rpfC* expression was likely attributed to its inhibitory effect on RpfG activity. Therefore, we suggested that dioctyldiethylenetriamine could affect the glutamic acid residues at positions 150 and 194 of RpfG, thereby inhibiting RpfG activity. This process subsequently triggered QQ and elicited alterations in the expression levels of key genes within the QS system.

### Dioctyldiethylenetriamine reduces the virulence of PXO99A by inhibiting the QS system

**Fig 3:**
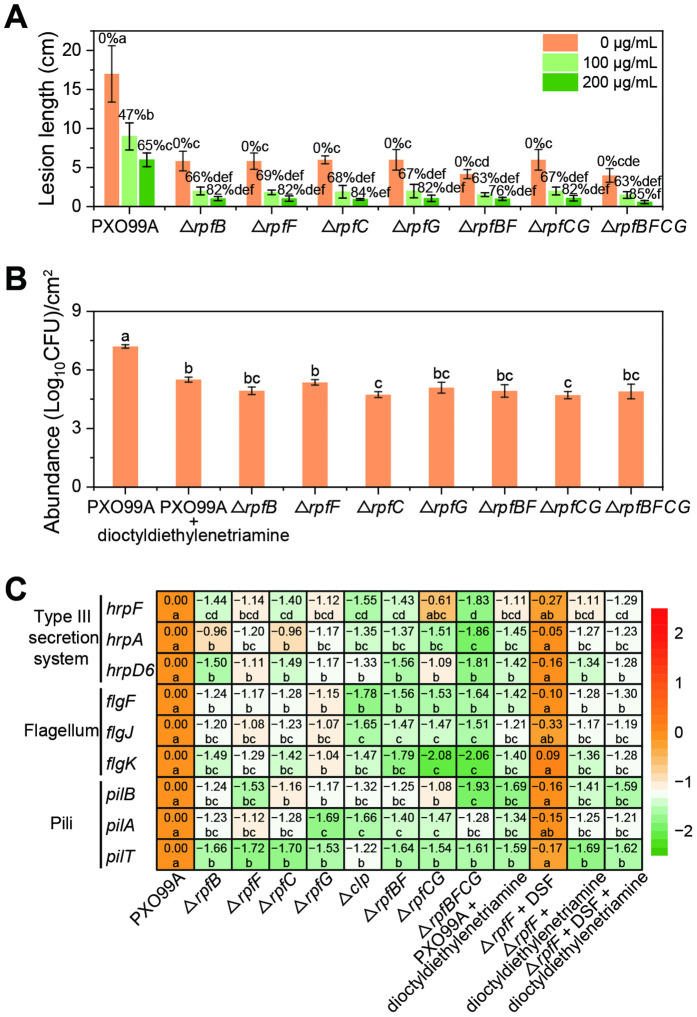
QS system drives in planta disease progression in *Xoo.* **(A)** The average lesion length of rice leaves inoculated with different strains and sprayed with different concentrations of dioctyldiethylenetriamine for 14 days. The number over the bar graphs indicates the efficacy of dioctyldiethylenetriamine against rice bacterial leaf blight. **(B)** Levels of colonization of rice leaves by different strains and the effect of dioctyldiethylenetriamine (200 μg/mL) on the colonization ability of strain PXO99A. **(C)** Expression levels of genes related to the type III secretion system, flagellum, and pili in different strains under various treatment conditions. Among these treatments, the concentration of dioctyldiethylenetriamine was 1.464 μg/mL, and the concentration of DSF was 10 μM. Numbers on the heatmap indicating relative gene expression levels were calculated using the log_2_2^-ΔΔCT^ method. Results shown in (A, B, C) were obtained independently in three experiments. Bar graphs denote mean ± SD. Error bars indicate SD. Results for the same strain under different concentrations of dioctyldiethylenetriamine treatment in **(A)**, results for different strains in **(B)**, and results for the same gene under different treatments in (C) were analyzed using one-way ANOVA, followed by Tukey’s multiple range test. Different letters in the bar graphs and heatmaps indicate statistically significant difference at *P* < 0.05.

To clarify whether dioctyldiethylenetriamine could reduce bacterial virulence by inhibiting the QS system, we first investigated whether impaired function of the QS system affected the virulence of the PXO99A on rice. We constructed the deletion mutants (Δ*rpfB*, Δ*rpfF*, Δ*rpfC*, Δ*rpfG*, Δ*rpfBF*, Δ*rpfCG*, and Δ*rpfBFCG*) of key genes of the QS system, and inoculated the mutants and PXO99A on rice leaves, respectively. After 14 days, the virulence of different strains was assessed by lesion length. The results showed that average lesion length of rice leaves inoculated with PXO99A was 17 cm, while lesion length of leaves inoculated with mutant strains significantly reduced (≤ 6 cm) (Fig 3A and S8 Fig). In addition, we quantified the number of bacteria on rice leaves. The results showed that the bacterial population of the mutants was significantly lower than that of the PXO99A (Fig 3B). Meanwhile, the expression levels of genes encoding bacterial virulence factors, such as the type III secretion system (T3SS) [38], flagella [39], and pili [37], were significantly lower in the QS system key gene deletion mutants and the c-di-GMP effector protein Clp deletion strain compared with the wild-type strain PXO99A (Fig 3C). These results indicated that the QS system was indispensable for the full virulence and colonization of *Xoo* in rice. Furthermore, QQ led to abnormal elevation of intracellular c-di-GMP levels in bacteria, which in turn inhibited the transcriptional activation of virulence-related genes by global transcriptional regulators Clp, resulting in the downregulation of virulence-related gene expression.

Next, we further evaluated the control efficacy of dioctyldiethylenetriamine against rice bacterial leaf blight (BLB). We sprayed 0, 100, and 200 μg/mL dioctyldiethylenetriamine on rice leaves inoculated with different strains and evaluated the control effect based on lesion length. The results showed that dioctyldiethylenetriamine was effective against BLB. At 100 and 200 μg/mL, control efficacies of dioctyldiethylenetriamine were 47% and 64% against the PXO99A, respectively, while the efficacies were 63–68% and 76–85% against the mutants, respectively (Fig 3A and S8 Fig). We further examined the colonization ability of bacteria following treatment with dioctyldiethylenetriamine (at the concentration of 200 μg/mL) and the expression levels of some virulence-related genes (the concentration of dioctyldiethylenetriamine was 1.464 μg/mL, which did not inhibit bacterial growth, S2 Fig). The PXO99A treated with dioctyldiethylenetriamine showed a similar weakening as that of mutant strains in colonization ability (Fig 3B). Meanwhile, the expression levels of genes encoding virulence factors such as the type III secretion system [38], flagella [39], and pili [37] of bacteria were significantly down-regulated, which was also observed in the mutants (Fig 3C). These results suggested that dioctyldiethylenetriamine could effectively reduce bacterial virulence by inhibiting the QS system of bacteria, thus controlling bacterial diseases.

Furthermore, to further confirm that the attenuation of bacterial virulence by dioctyldiethylenetriamine via QS system inhibition was attributed to its suppression of RpfG and subsequent interference with intracellular QS signal transduction, rather than disruption of DSF biosynthesis, we employed the Δ*rpfF* strain (in which DSF synthesis is significantly impaired due to *rpfF* gene deletion [31]) as the experimental subject. Three treatments were conducted respectively: Δ*rpfF* strain treated with DSF alone, Δ*rpfF* strain treated with dioctyldiethylenetriamine alone, and Δ*rpfF* strain co-treated with dioctyldiethylenetriamine and exogenous DSF. The transcriptional levels of virulence factor-encoding genes were detected using qRT-PCR. The results showed that supplementation with DSF alone significantly alleviated the downregulation of virulence-related genes in the Δ*rpfF* strain and restored their expression levels nearly to those of the wild-type PXO99A strain (Fig 3C). This indicated that exogenous DSF effectively compensated for the abnormal expression of virulence-related genes caused by DSF signal deficiency in the Δ*rpfF* strain. In the Δ*rpfF* strain, no significant difference in the expression levels of virulence-related genes was observed between the group co-treated with dioctyldiethylenetriamine and exogenous DSF and the group treated with dioctyldiethylenetriamine alone. Moreover, the gene expression levels under both treatments failed to be restored to the level of the wild-type strain PXO99A (Fig 3C). These findings demonstrated that alterations in DSF content did not affect the inhibitory effect of dioctyldiethylenetriamine on the expression of virulence-related genes. Therefore, we concluded that dioctyldiethylenetriamine reduced bacterial virulence by inhibiting the QS system, which was achieved through the specific suppression of RpfG activity, thereby interfering with the intracellular transduction process of QS signals.

### Indirect antibacterial effect of dioctyldiethylenetriamine enhances its direct antibacterial effect against bacteria

**Fig 4:**
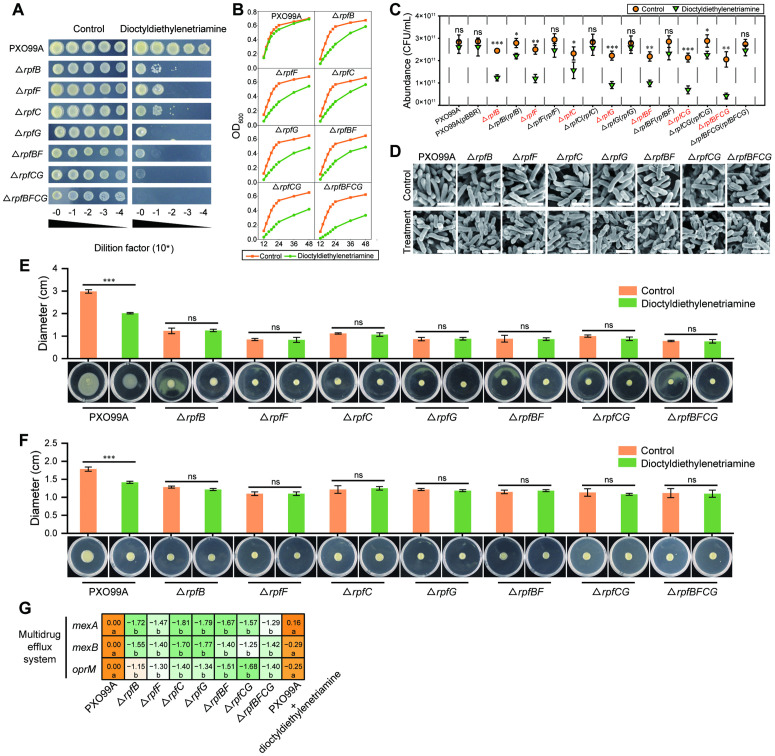
*In vitro* antibacterial activity of dioctyldiethylenetriamine against different bacterial strains. **(A)** Growth of different strains on medium containing dioctyldiethylenetriamine (1.6 μg/mL). **(B)** Growth curves of bacteria under different concentrations of dioctyldiethylenetriamine treatment (0 or 0.183 μg/mL). **(C)** Abundance of different strains after 16 h of incubation in conditions with and without dioctyldiethylenetriamine (0 or 0.183 μg/mL). Scatter plots denote mean ± SD. Error bars indicate SD. **(D)** Cell morphology of bacterial changes in response to dioctyldiethylenetriamine (0 or 0.366 μg/mL) by scanning electronic microscopy. Scale bar = 1 μm. **(E)** Swimming motility. The concentration of dioctyldiethylenetriamine is 0 or 0.183 μg/mL. **(F)** Swarming motility. The concentration of dioctyldiethylenetriamine is 0 or 0.183 μg/mL. Bar graphs denote mean ± SD. Error bars indicate SD. **(G)** Expression levels of multidrug efflux pump system-related genes in different strains and strain PXO99A treated with dioctyldiethylenetriamine (1.464 μg/mL). Numbers on the heatmap indicating relative gene expression levels were calculated using the log_2_2^-ΔΔCT^ method. Results shown in (A, B, C, D, E, F, G) were obtained independently in three experiments. Results for the same strain under different treatments in (C, E, F) were analyzed using one-way ANOVA followed by Tukey’s multiple range test, with “*” stands for statistically significant differences, *P* < 0.05; “**” stands for statistically significant differences, *P* < 0.01; “***” stands for statistically significant differences, *P* < 0.001; “ns” stands for not statistically significant difference. For **(G)**, the results of different treatments targeting the same gene were analyzed by one-way ANOVA followed by Tukey’s multiple range test; different letters in the figure indicate statistically significant differences at the *P* < 0.05 level.

Previous studies have demonstrated the direct antibacterial effect of dioctyldiethylenetriamine on bacteria [2,25]. And in this study, we found that dioctyldiethylenetriamine provided a higher control effect on diseases caused by the mutants, of which the key gene in the QS system were deleted, than the wild-type. Therefore, we further explored whether there was a link between the indirect antibacterial effect of dioctyldiethylenetriamine in inhibiting QS sensing and its direct antibacterial effect on bacteria. We found that the growth of the PXO99A and complemented strains was not significantly affected by dioctyldiethylenetriamine (Fig 4A and S9 Fig), while the growth of all the mutant strains was significantly inhibited (Fig 4A). Among the single mutant strains, deletion of the *rpfG* led to enhanced sensitivity to dioctyldiethylenetriamine. The ∆*rpfBFCG* with complete deletion of the *rpfB*, *rpfF, rpfC*, and *rpfG* was hardly able to grow on medium containing dioctyldiethylenetriamine. In addition, similar results were also obtained by liquid cultures of the above strains (Fig 4B, S10 Fig and S2 Table) as well as in tests of bacterial abundance counts (Fig 4C).

We used scanning electron microscopy to observe the effect of dioctyldiethylenetriamine on bacterial morphology. The results showed that in the absence of dioctyldiethylenetriamine, the cell morphology of the different strains was characterized by roundness and fullness and did not significantly differ from each other (Fig 4D and S11 Fig). This suggested that the deletion of key genes of the QS system in PXO99A did not change its cellular morphology. In the presence of dioctyldiethylenetriamine, all the cells of the mutant strains showed different degrees of damage, whereas the cell morphology of the PXO99A showed no significant change. Among them, the ∆*rpfG*, ∆*rpfBF*, ∆*rpfCG*, and ∆*rpfBFCG* showed the most serious cellular damage and even cellular breakage (Fig 4D and S11 Fig). We also found that bacterial motility, which is associated with bactericide resistance, was significantly inhibited in the PXO99A after dioctyldiethylenetriamine treatment, as well as in the mutant strains before and after dioctyldiethylenetriamine treatment compared to the untreated PXO99A. In addition, the swimming and swarming motility of the mutants were not significantly different after dioctyldiethylenetriamine treatment (Fig 4E and 4F). The expressions of multidrug efflux-related genes *mexA*, *mexB* and *oprM* in the tested strains were detected by qRT-PCR (Fig 3C). The results showed that compared to the PXO99A, the transcriptional levels of these genes were significantly reduced in the mutants. After dioctyldiethylenetriamine treatment, the expression levels of the *mexA*, *mexB* and *oprM* genes in the PXO99A were not changed significantly (Fig 4G). These results suggested that when the QS system was impaired, the motility and the expression of multidrug efflux-related genes of the bacteria were decreased, and their sensitivity to dioctyldiethylenetriamine was significantly enhanced. Additionally, we assumed that dioctyldiethylenetriamine might have other potential target sites. When the QS system was inhibited, it could further enhance the inhibitory activity of the bactericide on other targets, thereby exerting a more effective antibacterial effect.

The antibacterial activity assays of dioctyldiethylenetriamine in combination with other bactericides was conducted. Both PXO99A and ∆*rpfG* strains were treated with the mixture of dioctyldiethylenetriamine and kasugamycin (or zinc thiazole), respectively, with the ratio of 1:9. The synergistic effect was evaluated using the synergy coefficient method to assess interactions among different bactericides in the combination systems [40,41]. The results showed that for the PXO99A strain, both combinations of dioctyldiethylenetriamine with kasugamycin or zinc thiazole exhibited synergistic effects [synergy ratio (SR) of 1.774 and 1.563, respectively]. For the ∆*rpfG* strain, the combinations of dioctyldiethylenetriamine with either kasugamycin or zinc thiazole exhibited additive effects (SR of 1.089 and 1.229, respectively) (S3 Table). This indicated that dioctyldiethylenetriamine could not only inhibit RpfG function to disrupt the QS system, but also enhanced the direct antibacterial activity of other bactericides against the PXO99A strain.

### Dioctyldiethylenetriamine-mediated QQ is present in some bacteria containing RpfG

**Fig 5:**
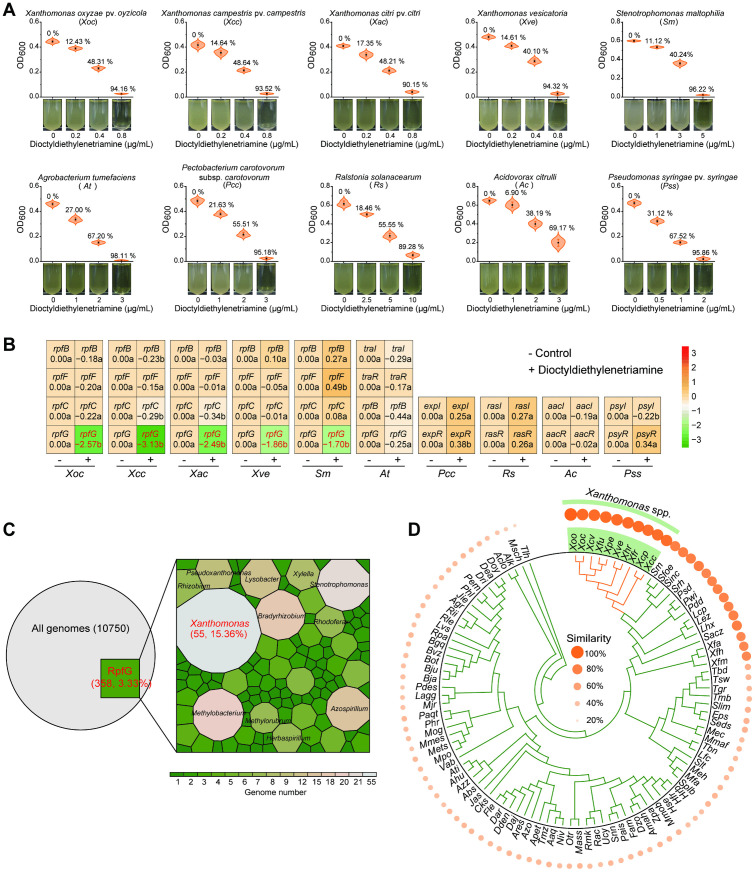
Effect of dioctyldiethylenetriamine on quorum sensing system of different pathogenic bacteria. **(A)** Determination of EC_50_ of dioctyldiethylenetriamine against different strains by turbidity method. The numbers on the violin plot indicate the inhibition rate of dioctyldiethylenetriamine on bacterial growth. **(B)** Expression levels of genes related to quorum sensing system in different strains treated with dioctyldiethylenetriamine (4 times EC_50_ value). Numbers on the heatmap indicating relative gene expression levels were calculated using the log_2_2^-ΔΔCT^ method. The results of the same gene under different treatments within the same strain were analyzed using one-way ANOVA followed by Tukey’s multiple range test, with different letters next to the numbers indicate statistically significant difference at *P* < 0.05. **(C)** Conservation and distribution of RpfG. Left: Distribution of gene encoding protein of the RpfG (KO term: K13815) within the KEGG database. Right: The genus to which the RpfG encoded protein belongs. Details were given in S8 Table. The larger the polygon area, the greater the number of bacteria contained in the genus. **(D)** Phylogenetic relationship of RpfG in 100 bacteria. Details were given in S9 Table. Results shown in (A, B) were obtained independently in three experiments.

To clarify whether dioctyldiethylenetriamine affects the QS system of other plant pathogenic bacteria, we first tested the inhibitory activity of dioctyldiethylenetriamine against 10 pathogenic bacteria, including *Xanthomonas oxyzae* pv. *oryzicola* (*Xoc*), *Xanthomonas campestris* pv. *campestris* (*Xcc*), *Xanthomonas citri* pv. *citri* (*Xac*), *Xanthomonas vesicatoria* (*Xve*), *Stenotrophomonas maltophilia* (*Sm*), *Agrobacterium tumefaciens* (*At*), *Pectobacterium carotovorum* subsp. *carotovorum* (*Pcc*), *Ralstonia solanacearum* (*Rs*), *Ac*, and *Pseudomonas syringae* pv. *syringae* (*Pss*). The results showed that dioctyldiethylenetriamine exhibited significant growth inhibition on *Xoc*, *Xcc*, *Xac*, *Xve*, *Sm*, *At*, *Pcc*, *Rs*, *Ac*, and *Pss*, with EC_50_ of 0.38, 0.37, 0.38, 0.38, 2.32, 1.39, 1.55, 4.50, 2.31, and 0.70 μg/mL, respectively (Fig 5A).

We further examined the effects of dioctyldiethylenetriamine (4 times EC_50_ value) treatment on the expression of key genes in the QS system by qRT-PCR. This concentration was selected because it showed no inhibitory effect on bacterial growth when applied at the bacterial initial OD_600_ of 0.4 as confirmed by S2 Fig and it induced the most significant suppressive effect on *rpfG* gene expression in the PXO99A strain (Fig 1E). The genes detected by qRT-PCR included the “*rpf*” cluster genes (*rpfB*, *rpfF*, *rpfC*, and *rpfG*) in *Xanthomonas* [32], the *expI* and *expR* in *Pectobacterium* [42], the *rasI* and *rasR* in *Ralstonia* [43], the *aacI* and *aacR* in *Acidovorax* [44], and the *psyI* and *psyR* in *Pseudomonas* [45]. The results showed that the *rpfG* expression level and the content of c-di-GMP of *Xanthomonas* spp. were significantly down-regulated and increased after dioctyldiethylenetriamine treatment, respectively (Fig 5B and S12 Fig). Following treatment with dioctyldiethylenetriamine, expression levels of key genes in the QS systems of other bacteria were not changed by more than 2 times (Fig 5B), and no significant alteration was observed in c-di-GMP content (S12 Fig), and there was no significant change in the content of acyl-homoserine lactones (AHLs, the QS signaling molecules) (S13 Fig). These results suggested that dioctyldiethylenetriamine had a specific inhibitory effect on the *rpfG*.

Next, we utilized the KEGG database (which contains 10,750 fully sequenced and consistently annotated genomes of organisms, including 1,157 eukaryotes, 9,162 bacteria, and 447 archaea) for the conservation analysis of RpfG-like proteins (KO term: K13815). The results showed that out of the total 10,750 organisms, only 358 bacteria (3.33%) encoded RpfG-like proteins, of which 55 (15.36%) belonged to the *Xanthomonas* (Fig 5C). Further phylogenetic analysis of the amino acid sequences of 100 bacteria containing RpfG-like protein showed that the RpfG sequences of *Xanthomonas* spp. were highly conserved (sequence similarity with *Xoo* > 90%). The RpfG sequences of *Xylella*, *Stenotrophomonas*, *Pseudoxanthomonas*, *Lysobacter* showed 70–82% similarity to *Xoo*, while the RpfG sequences of other bacteria showed less than 50% similarity to *Xoo* (Fig 5D).

We further examined the effects of dioctyldiethylenetriamine (4 times EC_50_ value) on the expression levels of *rpfB*, *rpfF*, *rpfC*, and *rpfG* in *Sm* (the amino acid sequence of RpfG with 80.47% similarity to that of PXO99A), and that of *rpfB*, *rpfG* (*rpfF* and *rpfC* have not been reported) and the key genes *traI* and *traR* of the QS system in *At* [46] (the amino acid sequence of RpfG with 38.52% similarity to that of PXO99A). The results showed that dioctyldiethylenetriamine only inhibited the expression of the *rpfG* in *Sm*, but with no significant effect on the expression levels of other genes (Fig 5B). Meanwhile, the c-di-GMP content in *Sm* was reduced after the treatment with dioctyldiethylenetriamine, whereas no obvious alteration in c-di-GMP levels was observed in *At* (S12 Fig). Multiple sequence alignment of RpfG amino acid sequences was performed among five *Xanthomonas* strains and the *Sm* strain. The results showed that the glutamic acid residues at positions 150 and 194 of RpfG in the PXO99A strain were highly conserved in the homologous sequences of all the tested strains (S14 Fig). Therefore, we suggested that dioctyldiethylenetriamine could specifically inhibit *rpfG* expression in *Xanthomonas* spp. and strains with high amino acid sequence similarity to the *rpfG* of *Xanthomonas* spp., thereby triggering QQ of these bacteria. In contrast, for bacteria with less similarity to the *rpfG* of *Xanthomonas* spp. or without *rpfG*, dioctyldiethylenetriamine might not affect their QS system. It was worth noting that the antibacterial activity of dioctyldiethylenetriamine against the *At* and *Pcc* strains (whose QS systems might not be affected by dioctyldiethylenetriamine) was higher than that against the *Sm* strain (whose QS system might be affected by dioctyldiethylenetriamine). This result suggested that the antibacterial effect of dioctyldiethylenetriamine varies among on different bacterial strains, and this difference might be related not only to its QQ effect but also to the direct antibacterial action that was independent of RpfG. Previous studies reported that dioctyldiethylenetriamine exhibits differential direct antibacterial activity against diverse bacteria, with stronger activity against strains of the genus *Xanthomonas* [2]. This also explained why the EC_50_ value for *Sm* remained high despite the impact of dioctyldiethylenetriamine on its QS system, whereas the EC_50_ values for *Xanthomonas* spp. strains were significantly lower.

### Impairment of the QS system reduces the interspecific competitive ability of *Xoo*

**Fig 6:**
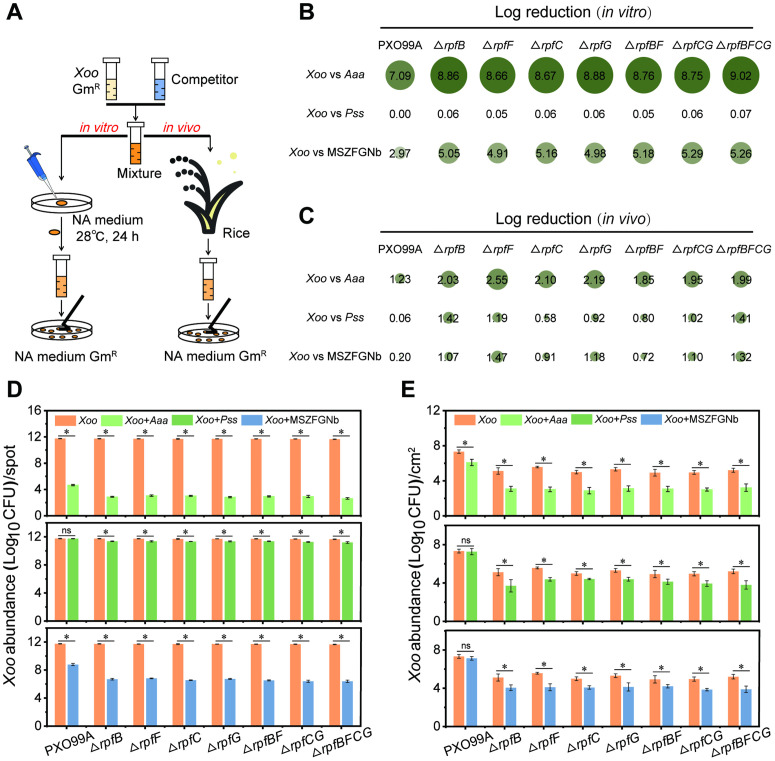
The effect of QS system on the interspecific competitiveness of *Xoo.* **(A)** Working model for *in vivo* and *in vitro* assessment of the effect of QS systems on the competitive ability of *Xoo* strains. **(B)** Differences in *in vitro* assay of the abundance of PXO99A (wild type) and gene deletion mutants surviving in the presence or absence of competing bacteria *Aaa*, *Pss*, and MSZFGNb based on log reduction analysis (Log_10_ CFU from the single culture group − Log_10_ CFU from the competitive group). Larger circle diameters and darker circle colors represent larger log reduction. **(C)** Differences in *in vivo* assay of the abundance of PXO99A and gene deletion mutants surviving in the presence or absence of competing bacteria *Aaa*, *Pss*, and MSZFGNb based on log reduction analysis. Larger circle diameters and darker circle colors represent larger log reduction. **(D)** *In vitro* assay of the abundance of PXO99A and gene deletion mutants surviving in the presence or absence of competing bacteria *Aaa*, *Pss*, and MSZFGNb. **(E)** *In vivo* assay of the abundance of *Xoo* strains in rice lesion sites caused by PXO99A and gene deletion mutants in the presence and absence of competing bacteria *Aaa*, *Pss*, and MSZFGNb. Results shown in (B, C, D, E) were obtained independently in three experiments. Bar graphs denote mean ± SD. Error bars indicate SD. Results were analyzed using one-way ANOVA followed by Tukey’s multiple range test, with “*” stands for statistically significant difference at *P* < 0.05, “ns” stands for not statistically significant.

In natural environments, the infection ability of *Xoo* on rice was not only directly regulated by its own virulence, but also closely related to its ability to survive in complex microbial communities. In order to clarify whether the competitive ability and survival advantage of *Xoo* in the natural environment would be weakened after its QS system was inhibited, we selected *Acidovorax avenae* subsp. *avenae* (*Aaa*, which causes bacterial leaf streak of rice [47]), *Pss* (which causes rice bacterial leaf spot [48]), and a rice endophytic bacterium (MSZFGNb, which belongs to the *Pseudomonas* spp.) as competitors of *Xoo* for the competition assays *in vitro* and *in vivo* (Fig 6A). In an *in vitro* competition assay, we co-incubated *Xoo*, including wild-type strain PXO99A and mutant strains (∆*rpfB*, ∆*rpfF*, ∆*rpfC*, ∆*rpfG*, ∆*rpfBF*, ∆*rpfCG*, and ∆*rpfBFCG*), with each of the three competing strains (*Aaa*, *Pss*, and MSZFGNb) for 24 h in NA medium and subsequently the number of *Xoo* was counted. Analysis of the log reduction (Log_10_ CFU from the single culture group − Log_10_ CFU from the competitive group) revealed that all seven mutants had significantly larger log reduction compared to the PXO99A, indicating a significant reduction in their competitive ability compared to the wild-type strain. Among them, *Aaa* had the most significant inhibitory effect on *Xoo*, followed by MSZFGNb (Fig 6B and 6D). This suggested that impairment of the QS system negatively affected the ability of *Xoo* to survive in the environment.

To validate the results of the *in vitro* assay and to explore the effect of the impaired QS system on the viability of the *Xoo in vivo*, we also conducted a competition assay in rice. We made bacterial suspensions of the PXO99A and each of the seven mutants by mixing them with competing strains, respectively, and then inoculated them onto rice leaves. After 14 days, the abundance of *Xoo* isolated from diseased leaves was tested. The results showed that compared with the mixed-inoculated PXO99A, the number of mutants isolated from the diseased leaves was significantly reduced, and the log reduction was significantly increased (Fig 6C and 6E). These results suggested that impairment of the QS system significantly reduced the ability of *Xoo* to survive when competing with other bacteria *in vitro* and in rice. The expression of genes in type VI secretion system (T6SS) [49] associated with bacterial competitiveness in *Xoo* was detected by qRT-PCR. The results showed that compared to PXO99A, the transcriptional levels of these genes in the mutant strain were almost significantly reduced. Following treatment with dioctyldiethylenetriamine, the expression levels of these genes in the PXO99A strain also exhibited a decreasing trend (S15 Fig). Therefore, we suggested that impairment of the QS system of *Xanthomonas* spp., not only directly impaired the virulence of the bacteria, but also significantly reduced its competitiveness in the ecological niche.

## Discussion

**Fig 7:**
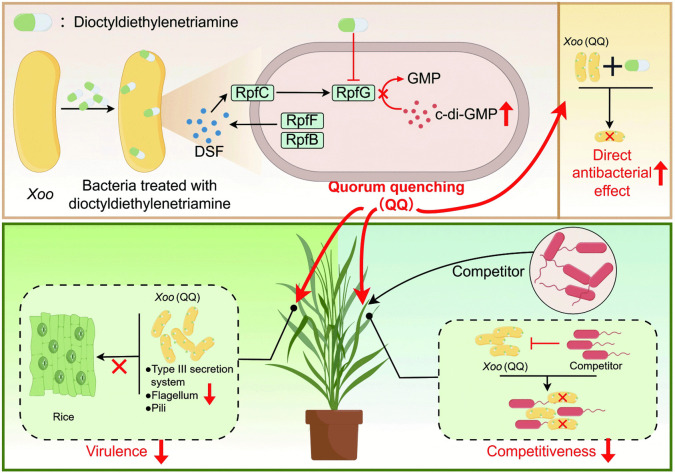
Dioctyldiethylenetriamine has an indirect antibacterial effect by inhibiting the QS system of bacteria. Dioctyldiethylenetriamine exerted a significant inhibitory effect on *rpfG* expression in *Xoo* at both transcriptional and translational levels and inhibited the phosphodiesterase activity of RpfG. This inhibition blocked the intracellular transduction of QS signals, triggered QQ, and led to an abnormal accumulation of intracellular c-di-GMP. *Xoo* strains inhibited by dioctyldiethylenetriamine showed a decrease in colonization ability and virulence factor expression, which directly led to a decrease in bacterial virulence. At the same time, the QQ of *Xoo* strains reduced their competitiveness in interspecific competition and lowered their ecological niche. In addition, the indirect antibacterial effect of dioctyldiethylenetriamine against strain *Xoo* further enhanced its direct antibacterial effect, thereby increasing its antibacterial activity. This figure was drawn using Figdraw (URL: https://www.figdraw.com).

In the field control of plant bacterial diseases, the commonly used agents include bactericides, plant immunity inducers and inhibitors of virulence factors. They achieve effective disease management by acting as a direct antibacterial effect against pathogenic bacteria [2], inducing the immune defense response of the plant itself [50], or weakening the virulence of the bacteria [12], respectively. In addition, some antagonistic bacteria not only can secrete antibacterial substances to inhibit or kill pathogenic bacteria [51], but also can induce plants to initiate their own defense responses to resist pathogen infestation [52], showing both direct and indirect antibacterial effects on pathogenic bacteria. However, research on existing agents is often limited to the analysis of a single antibacterial mechanism. For example, research on bactericides usually focuses on elucidating their direct antibacterial mechanisms; research on plant immunity inducers focuses on how they induce plant defense responses; and research on inhibitors of virulence factors (such as QSIs or type III secretion system inhibitors) focuses only on identifying the specific ways in which they weaken the virulence of the pathogenic bacteria. These studies often neglect that in addition to the main mechanism of action, there may be other potential mechanisms that have not yet been explored. It is of great significance to identify the multiple action mechanisms of agents for optimizing the prevention and control strategy of diseases, improving the control effect of agents and reducing the risk of bacterial resistance. In view of this, transposon sequencing technology was applied in this study to explore the possible action sites of dioctyldiethylenetriamine (Fig 1). We found that the bactericide dioctyldiethylenetriamine that was previously reported to have a direct antibacterial effect, exhibited a new mode of action in this study (Fig 7). Specifically, dioctyldiethylenetriamine was able to effectively inhibit the QS system responsible for regulating group behavior and virulence factor expression in *Xoo*. This indirect antibacterial effect, which weakened the virulence of bacteria, was ecologically and biologically important for the protection of plants from bacterial infection. Thus, our results added a dimension to the understanding of the mechanism of bactericides, demonstrating that bactericides could control diseases not only by direct antibacterial effect that inhibited bacterial growth or killed bacteria, but also by indirect antibacterial effect that reduced bacterial virulence through inhibition of the QS system.

The QQ of bacteria induced by dioctyldiethylenetriamine showed a “non-canonical” pattern. Earlier studies found that QQ is achieved by three main pathways, including inhibition of signal molecule production, degradation of signal molecule, and inhibition of signal molecule binding to receptor proteins, which usually results in changes in the content of the signal molecule [53]. For example, in most gram-negative bacteria, QQ is usually mediated by a combination of LuxI homologous genes (responsible for the synthesis of signal molecule) and LuxR homologous genes (which act as receptor for signal molecule and regulate the transcription of downstream genes), and inhibition of both types of genes often causes changes in the content of signal molecules [43,45]. However, in the present study, we found that the QQ induced by dioctyldiethylenetriamine did not change the content of the QS signal molecules DSF in the PXO99A, which was significantly different from the three main QQ mechanisms previously identified, all of which involved in the changes in the content of the signal molecules. The results of molecular docking, *in vitro* PDE activity assay and bacterial intracellular c-di-GMP content detection indicated that dioctyldiethylenetriamine could inhibit the activity ofRpfG (an intracellular response regulator responsible for signal transduction). This inhibitory effect on RpfG might block the intracellular transduction of QS signals and trigger an abnormal accumulation of intracellular c-di-GMP, and reduce bacterial virulence (Figs 1–3). It was worth noting that RpfC, the receptor protein of the signal molecule in *Xanthomonas* spp., did not directly regulate downstream genes, but regulated the PDE activity of RpfG through phosphorylation, and then mediated the expression of downstream genes. Thus, RpfG did not directly affect the content of signal molecules. This “non-canonical” QQ mechanism demonstrated by dioctyldiethylenetriamine provides a new perspective for the development of QSIs. Furthermore, the *in vitro* inhibitory effect of dioctyldiethylenetriamine on RpfG activity (Fig 2C) was weaker than its inhibitory effect in bacteria (Fig 2E–2I). This difference might be due to the fact that the *in vitro* reaction system was difficult to fully simulate the complex intracellular microenvironment of bacteria and did not possess the unique transcriptional regulatory pathway mediated by the c-di-GMP effector protein Clp in bacteria. Based on this, we speculated that in the PXO99A strain, dioctyldiethylenetriamine, in addition to directly inhibiting RpfG activity, could also further enhance the inhibition of RpfG activity through the Clp-mediated transcriptional regulatory pathway or other potential pathways, and thereby intensifying the QQ effect of bacteria.

As an intercellular communication system widely existing in microorganisms, the QS system plays a crucial role in pathogenesis process of bacteria by regulating the expression of virulence factors [54]. In this study, we demonstrated that dioctyldiethylenetriamine could effectively protect crops against PXO99A by inhibiting its QS system. Specifically, when the key genes *rpfB* (involved in DSF turnover [55]), *rpfF* (responsible for DSF biosynthesis [56]), *rpfC* (a receptor protein for DSF [56]), and *rpfG* (involved in DSF transduction [56], which is inhibited by dioctyldiethylenetriamine) in the QS system were deleted in PXO99A strain, the colonization ability and the expression level of virulence related genes in rice leaves were significantly decreased, and eventually led to the reduction of bacterial virulence. A similar phenomenon occurred in *Xoo* strains in which the virulence significantly decreased with the deletion of *rpfB* or *rpfC* [57]. It was worth noting that no significant difference in virulence was observed in the single mutant strains (∆*rpfB*, ∆*rpfF*, ∆*rpfC*, or ∆*rpfG*), double mutant strains (∆*pfBF* or ∆*rpfCG*), or even quadruple mutant strain (∆*rpfBFCG*) (Fig 3). This phenomenon has also been reported in other plant pathogenic bacteria. For example, deletion of either *rasI* or *rasR*, the key regulatory genes of the QS system in *Rs* [43], resulted in a significant reduction in bacterial virulence, but there was no significant difference in virulence between different deletion mutants. These results suggested that the regulation of the QS system was a complex and delicate process, and that the deletion of any key genes could lead to the loss of QS function in bacteria, which in turn weakens their virulence. Therefore, interference with different key genes in QS system (such as inhibition of signal molecule production, degradation of signal molecule, inhibition of signal molecule binding to receptor protein or blocking the transduction of signal molecule, etc.) might have the same effect in regulating bacterial virulence.

In addition, our study revealed that impaired function of the QS system, both *in vitro* and *in planta*, was more conducive to the direct antibacterial effect of dioctyldiethylenetriamine on strain PXO99A (Figs 3 and 4). This QS-mediated protection appeared to be associated with QS-controlled phenotypes. Specifically, bacteria could regulate their motility through QS systems to actively migrate to a more favorable environment, thereby improving their tolerance to antibacterial substances such as antibiotics [58]. However, a significant reduction in bacterial swimming and swarming motility were observed in all our constructed QS key gene deletion mutant strains (Fig 4). Therefore, the QQ effect triggered by dioctyldiethylenetriamine not only weakened bacterial virulence by reducing the expression of colonization and virulence factors, but also effectively prevented bacteria from escaping the direct antibacterial effect of dioctyldiethylenetriamine through reduced motility, thus conferring a more efficient disease-control effect of dioctyldiethylenetriamine. Moreover, the expression levels of multidrug efflux-related genes *mexA*, *mexB*, and *oprM* decreased in the QS key gene deletion mutants and this might also be one of the reasons for their enhanced sensitivity to dioctyldiethylenetriamine (Fig 4G).

QS system is not only an important tool for intraspecific communication in bacteria, but also a key defense mechanism against interspecific competition [49]. When bacterial competitors use the T4SS or T6SS to translocate toxic effector proteins into other bacterial cells to induce cell death, QS of the bacteria might enhance protection against killing activity conferred by T4SS/T6SS of competing strains as a counterattack strategy [49,59]. Thus, inhibition of QS might weaken interspecific competitiveness of bacteria. Previous studies have found that typical QSIs such as quercetin [16], curcumin [17], vanillin [18], and furanone [19] have broad-spectrum quorum-sensing inhibitory effect. For example, quercetin can effectively inhibit the QS of bacteria such as *Pa* [60], *Salmonella* [61], and *Vibrio cholerae* [62]; curcumin also has significant inhibition on the QS system of *Pa* [63], *Vibrio* spp. [64], and *Bs* [65]. In contrast, dioctyldiethylenetriamine, which has QS inhibition function found in this study, appeared to have inhibitory activity only on the QS system of *Xanthomonas* spp. and strains with high amino acid sequence similarity to the *rpfG* of *Xanthomonas* spp., presenting specificity in affecting RpfG-like proteins (Fig 5). In this case, dioctyldiethylenetriamine caused QQ of *Xanthomonas* spp., which might selectively reduce the interspecific competitiveness of bacteria of this genus, thus further mitigating diseases caused by this genus. Our results showed that impaired QS function reduced the ability of *Xoo* strain in interspecific competition, both in the *in vitro* and *in planta* (Fig 6), and concurrently downregulated the expression levels of partial genes in T6SS that were associated with bacterial competitiveness (S15 Fig) [49]. Given that *Xanthomonas* spp. account for nearly one-third of the top ten bacterial plant pathogens in the field of crop protection [66], we believed that dioctyldiethylenetriamine, by QQ of *Xanthomonas* spp. might provide a new pathway for the prevention and control of diseases caused by *Xanthomonas* spp. while maintaining the diversity of microbial communities.

In conclusion, this study demonstrated that the bactericide, dioctyldiethylenetriamine, exhibits diverse modes of action in disease control, and that in addition to the direct antibacterial effect to disease control, it can also be used to control disease through indirect antibacterial effect by inhibiting the QS system to reduce bacterial virulence, which enriched our understanding of the mechanism of action of bactericides. On the other hand, the indirect antibacterial effect of dioctyldiethylenetriamine against *Xanthomonas* spp. achieved by inhibiting the QS system also enhanced its direct antibacterial effect against the bacterium, which provided a theoretical basis for the development of “RpfG-based” indirect antibacterial agents with both efficient antibacterial activity and ecological safety.

## Materials and methods

### Bactericide, bacterial strains, and growth conditions

The bactericides in technical-grade in this study were dioctyldiethylenetriamine (IUPAC name: N^1^-octyl-N^2^-[2-(octylamino)ethyl] ethane-1,2-dia-mine, CAS No. 57413-95-3, also known as Xinjunan in China, which was provided by Greenland Chemical Co., Ltd. of China), kasugamycin (CAS No. 6980-18-3) and zinc thiazole (CAS No. 3234-62-6), which were dissolved in methanol, sterile water, and dimethyl sulfoxide, respectively. The bacterial strains used in this study were listed in S4 Table. *Xanthomonas* spp.*, Pseudomonas* spp., and *Acidovorax avenae* subsp. *avenae* (*Aaa*) strains were grown in Nutrient Broth (NB; 5 g peptone, 10 g sucrose, 1 g yeast extract, and 3 g beef extract in 1 L water) medium supplemented with the appropriate antibiotics. *Escherichia coli* (*E. coil*), *Ralstonia solanacearum*, *Stenotrophomonas maltophilia*, and *Agrobacterium tumefaciens* strains were grown in Luria-Bertani (LB; 5 g yeast extract, 10 g tryptone, and 10 g NaCl in 1 L water) medium with appropriate antibiotics. The medium for β-galactosidase activity assay was AT (2 g (NH_4_)_2_SO_4_, 0.078 g MgSO_4_, 0.0076 g CaCl_2_, 0.005 g FeSO_4_·7H_2_O, 0.0022 g MnSO_4_·H_2_O, 10.7 g KH_2_PO_4_, and 0.5% Glucose in 1 L water). The non-selective medium used for the bacterial one-hybrid assay contained 15 g/L agar, 3.39 g/L Na₂HPO₄, 3 g/L KH₂PO₄, 0.5 g/L NaCl, 1 g/L NH₄Cl, 20 mL of 20% glucose, 10 mL of 20 mM adenine hydrochloride (adenine HCl), 100 mL of 10 × His Dosup, 1 mL of 1 M MgSO₄, 1 mL of 1 M thiamine hydrochloride (thiamine HCl), 1 mL of 10 mM ZnSO₄, 30 μg/mL kanamycin, 25 μg/mL chloramphenicol, and 12.5 μg/mL tetracycline. Unless otherwise stated, the default concentrations of antibiotics used in this study were as follows: kanamycin (Km; 50 μg/mL), gentamicin (Gm; 40 μg/mL) and streptomycin (S; 50 μg/mL).

### Construction of plasmids and mutant strains

Gene deletion mutants and rpfG-GFP-PXO99A mutant were constructed by homologous single cross-over method using the suicide vector pK18mobSacB [67]. The flanking regions of each gene were amplified using PCR and ligated into the vector pK18mobSacB. The constructed vectors were transformed into wild-type strains by electroporation. Recombinants were selected in NAN (NB medium without sucrose and 1.75% (w/v) agar) with added Km. The recombinants were incubated in antibiotic-free NBN (NB medium without sucrose) with shaking for 10 h and then spread in NAS (NB medium with 10% (w/v) sucrose and 1.75% (w/v) agar), and when single colonies grew, the colonies were picked and verified by PCR. Genetic complementation or constitutive expression was performed by constructing the recombinant vector pBBR1MCS5, which was provided in trans into corresponding bacterial strains [67]. Heterologous expression of RpfG or RpfG^E150A, E194A^ protein was performed by constructing recombinant vector pET28a. A full-length DNA fragment encoding the RpfG protein or RpfG^E150A, E194A^ was cloned using the PXO99A genome as the template. And the DNA fragment was ligated to the pET28a vector to generate the pET28a::*rpf*G or pET28a::*rpf*G ^E150A, E194A^ vector with an N-terminal His tag, and the plasmid was introduced into *E. coil* BL21 (DE3) for expression of RpfG or RpfG^E150A, E194A^ protein. For β-Glucuronidase (GUS) activity assays, the recombinant pHM1 vectors were constructed and introduced in trans into the corresponding bacterial strains. The specific primers used to construct the mutants were shown in S5 Table and the plasmids were shown in S6 Table.

### Library preparation, sequencing and analysis for transposon insertion sequencing (Tn-seq)

A collection of mutants previously formed in this laboratory by random insertion of transposons (strain PXO99A) was treated with 0.5 μg/mL of dioctyldiethylenetriamine. The DNA libraries for Tn-seq analysis were constructed, as described previously [28], and sequenced using an Illumina PE150 sequencer. Differential genes between samples under different treatments were selected by multiplicity of fold change > 2, and differential genes were analyzed using Kyoto Encyclopedia of Genes and Genomes (KEGG).

### RNA preparation and qRT-PCR analysis

Bacteria were cultured in NB or LB medium until mid-exponential. After bacteria were centrifuged and collected, the bacterial concentration was normalized to OD_600_ = 0.4 using 0.9% NaCl. Next, different concentrations of dioctyldiethylenetriamine or 10 μM DSF were added to the normalized cultures. After incubation at 28°C and 60 rpm for 24 hours, the samples were centrifuged and washed. The total RNA was extracted using a Bacterial RNA Rapid Extraction Kit (Jinbaite, China) according to the manufacturer’s instructions. RNA concentrations were measured by a Nanodrop ND-1000 UV Spectrophotometer (Thermo Fisher, USA). Then, 1 μg RNA from each sample was used to generate total cDNA by using the PrimeScript RT Master Kit (Takara, Japan). The qRT-PCR was performed using TB Green Premix Ex Taq II (Takara, Japan) with a CFX96 real-time PCR detection system (Bio-Rad, USA). The transcript level of *gyrB* or *actin* gene was amplified as the internal control. Each reaction was performed in three biological repeats, and the relative gene expression levels were calculated using the log_2_2^-ΔΔCT^ method [28]. All the primers used in this study were listed in S5 Table.

### DSF extraction and quantification

The *Xanthomonas oryzae* pv. *oryzae* (*Xoo*) strain was cultured on NB medium until mid-exponential, then subjected to centrifugation, washed with sterile 0.9% NaCl, and normalized to an OD_600_ of 0.4. Next, different concentrations of dioctyldiethylenetriamine were added to the normalized cultures and incubated for 48 h at 28 °C, 60 rpm. The 200 mL of bacterial supernatant was collected by centrifugation and the supernatant was acidified to a pH of 4.0 with diluted HCl and then extracted twice with an equal volume of ethyl acetate. After evaporation of ethyl acetate by rotary evaporator, the residues were dissolved using methanol and the solution was filtered using a membrane filter with a pore size of 0.22 μm. The filtered extract was injected into a C_18_ reversed-phase HPLC column and eluted with methanol and water (80:20 [vol/vol] water containing 0.1% formic acid) at a flow rate of 1.0 mL/min [68]. Detection of DSF was performed using a Waters 2475 UV-visible detector at 210 nm, and signal integration and monitoring were performed using Empower Pro software. DSF standard solution was used as a control, and the DSF content in the sample was calculated based on the peak area.

### Detection of protein expression by western blot and fluorescence microscopy

After the strains were treated with different concentrations of dioctyldiethylenetriamine for 24 h, the precipitate was collected by centrifugation and resuspended using phosphate buffered saline (PBS). After the bacteria were broken by ultrasonic cell crusher, the supernatant was collected by centrifugation and protein concentration was determined by BCA assay kit. Protein (10 μg) samples were resolved by 12.5% SDS-PAGE and electro-transferred onto the PVDF membrane for western blot assay. The RpfG-GFP or GFP protein in the membrane was tested with anti-GFP tag rabbit antibody, and horseradish peroxidase-conjugated secondary antibody (goat anti-rabbit) was used for chemiluminescent detection of bound ligands for all blot assays. Detection was performed using Western ECL reagents. Images were digitized using the Chemidoc MP Imaging System (Bio-Rad, USA) and the strips were analyzed for grayscale values using ImageJ software.

After rpfG-GFP-PXO99A or GFP-PXO99A was treated strain with different concentrations of dioctyldiethylenetriamine for 24 h, the green fluorescence (488 nm) was observed using a laser confocal microscope (Nikon, Japan). The intensity of fluorescence was analyzed using ImageJ software.

### Purification of RpfG protein

The *E. coil* BL21(DE3)-rpfG strain was cultured in 400 mL of LB (Km) medium at 37°C with shaking until the OD_600_ reached 0.5, and then induced by the addition of 0.5 mM of isopropyl β-D-1-thiogalactopyranoside (IPTG) at 16°C for 24 h. The bacteria cells were collected by centrifugation and resuspended in 20 mL of PBS, and then the bacteria were broken by ultrasonic cell crusher. Cell debris was removed by centrifugation, and the supernatant was passed through 1 mL of Ni-NTA before being washed with 50 mL of wash buffer to remove heterogeneous proteins, followed by 15 mL of elution buffer to elute RpfG protein [36]. Protein purity was assessed by SDS-PAGE and protein concentration was determined using BCA assay kit (Leagene, China).

### Phosphodiesterase (PDE) activity assay *in vitro*

PDE activity was assayed in a 100 μL reaction volume containing 50 mM Tris-HCl (pH 7.6), 50 mM NaCl, 5 mM MnCl_2_, 20 mM MgCl_2_, 50 μM c-di-GMP and 2 μM target protein. The reaction mixture was incubated at 28°C for 1 h, followed by boiling for 10 min to stop the reaction. Then, the reaction mixture was centrifuged at 12,000 rpm for 10 min to remove precipitated [36]. The content of cyclic dimeric GMP (c-di-GMP) in the supernatant was determined by microbial cyclic diguanosine acid ELISA Kit (Caobenyuan, China).

### Molecular docking analysis

The three-dimensional structure of the RpfG and RpfG^E150A, E194A^ were predicted using AlphaFold 3 (https://alphafoldserver.com). Molecular docking simulations between dioctyldiethylenetriamine and the RpfG or RpfG^E150A, E194A^ were performed using Discovery Studio software (Accelrys Inc., San Diego, CA, USA). The binding affinity between dioctyldiethylenetriamine and RpfG or RpfG^E150A, E194A^ was assessed based on binding energy, where lower binding energy indicates a more stable interaction between the ligand and receptor [69]. Finally, the molecular docking results were visualized using PYMOL (Delano Science, San Carlos, CA, USA).

### C-di-GMP extraction and quantification

Bacteria were cultured in NB medium until mid-exponential. After bacteria were centrifuged and collected, the bacterial concentration was normalized to OD_600_ = 0.4 using 0.9% NaCl. Next, different concentrations of dioctyldiethylenetriamine were added to the normalized cultures. After incubation at 28°C and 60 rpm for 24 hours, the samples were centrifuged and washed. Bacterial pellet from 10 mL culture was broken by ultrasonic crusher and the protein was quantified by BCA assay (Leagene, China). Bacterial pellet from 40 mL was extracted c-di-GMP using 0.6 M HClO_4_ and 2.5 M K_2_CO_3_ [36]. The content of c-di-GMP in the samples was determined by microbial cyclic diguanosine acid ELISA kit (Caobenyuan, China). The c-di-GMP concentration was calculated as follow: c-di-GMP concentration = c-di-GMP content in bacterial pellets (pmol)/protein content in bacterial pellets (mg).

### Bacterial one-hybrid assay

A bacterial one-hybrid system was utilized to verify the potential interaction between the Clp protein and the promoter of *rpfG* gene. The coding region of Clp protein was cloned into the PTRG vector, while the promoter region of *rpfG* gene was inserted into the pBXcmT vector. Subsequently, the two recombinant plasmids were co-transformed into competent cells of *E. coli* XL1-Blue MRF’ Kan strain. If there was an interaction between Clp protein and the *rpfG* promoter region, the *E. coli* strain harboring both PTRG-Clp and pBXcmT-PrpfG recombinant plasmids could grow normally on the selective medium. This selective medium was prepared by adding 3-amino-1,2,4-triazole (3-AT) with a final concentration of 5 mM and streptomycin (Str) with a final concentration of 8 μg/mL to the non-selective medium.

### GUS activity assay

The GUS reporting system was employed to investigate the effect of Clp on the activity of the *rpfG* promoter. The test bacteria were cultured in NB medium until mid-exponential. After bacteria were centrifuged and collected, the bacterial concentration was normalized to OD_600_ = 0.4 using 0.9% NaCl. The GUS activity assay was performed as follows: (1) the semi-quantitative method (solid culture). The 5 μL of normalized bacterial suspension was inoculated onto NA medium containing 50 μg/mL 5-Bromo-4-chloro-3-indolyl-β-D-glucuronide acid (X-gluc). For treatment groups, 1.464 μg/mL dioctyldiethylenetriamine or 16 μmol/L c-di-GMP was added to the medium, while a control group without dioctyldiethylenetriamine or c-di-GMP was included. All samples were incubated at 28°C for 5 days, and colony color development was observed and recorded. (2) Quantitative assay (liquid culture). To normalized bacterial cultures, dioctyldiethylenetriamine at a final concentration of 1.464 μg/mL or c-di-GMP at 16 μmol/L was added as treatment groups, while cultures without addition of dioctyldiethylenetriamine or c-di-GMP served as the control group. After incubation at 28°C with shaking at 60 rpm for 24 h, 25 μL of each treated culture was collected, and cells were harvested by centrifugation. Then, 1 mL of Sonic buffer (5 mmol/L EDTA, 1% Triton X-100, 10 mmol/L β-mercaptoethanol, and 20 mmol/L Tris-HCl) was added to the cell pellet, followed by thorough mixing and cell lysis. After centrifugation, 125 μL of the supernatant was transferred and mixed with 375 μL of GUS reaction buffer (50 mmol/L Na_3_PO_4_, 1 mmol/L MUG, 10 mmol/L β-mercaptoethanol, and 0.1% Triton X-100). Reactions were carried out at 37°C for 30 min and terminated by adding 0.2 mol/L Na_2_CO_3_. Fluorescence was measured using a microplate reader at an excitation wavelength of 365 nm and an emission wavelength of 456 nm, and GUS activity was calculated accordingly. GUS activity was expressed as nM/min/OD_600_, where nM represents the amount of 4-methylumbelliferone (MU) produced by hydrolysis of 4-methylumbellifery-β-D-glucuronide (MUG) per minute in 1 mL of bacterial culture adjusted to OD_600_ = 1.0.

### Antibacterial activity in the pot experiment

The *Xoo* strains (OD_600_ = 0.5) were inoculated onto rice (40-day-old) leaves using the leaf-cutting method, and 24 h after inoculation, the entire rice plant was sprayed with 0, 100 or 200 μg/mL commercial agent of dioctyldiethylenetriamine until droplets fell, respectively [70].

The progression of disease was assessed 14 days after inoculation. In addition, 1 cm^2^ of rice leaves were cut from the disease-health junction, sterilized with 75% ethanol for 30 s, added to 1 mL of NB medium, and crushed using a ball mill. The suspension was spread on NA (NB medium with 1.75% (w/v) agar) and counted after single colonies grew.

### *In vitro* antibacterial activity

The *Xoo* strains were cultured until mid-exponential phase in NB medium, then subjected to centrifugation, washed with sterile 0.9% NaCl, and normalized to an OD_600_ of 1.0. A serial dilution was produced by adding 0.9% sterile NaCl to the resulting bacterial solution, and then 5 μL of the bacterial solution was inoculated onto NA medium containing 0 or 1.6 μg/mL dioctyldiethylenetriamine [71]. The plates were incubated at 28°C for 2–3 days before being assessed for colony growth.

### Growth assay

The *Xoo* strains were cultured until mid-exponential phase in NB medium, then subjected to centrifugation, washed with sterile 0.9% NaCl, and normalized to an OD_600_ of 0.5. Next, 1% of the normalized cultures were transferred to NB medium containing 0 or 0.183 μg/mL dioctyldiethylenetriamine and incubated at 28°C with agitation at 180 rpm in a shaker incubator. The OD_600_ of the bacterial solution at different time points was measured using a microplate reader (Thermo, USA) [71]. In addition, 100 μL of the bacterial solution incubated for 16 h was taken, gradient diluted and spread in NA medium, and the number of colonies was counted after single colonies were grown.

### Bacterial morphology observation

*Xoo* strains were cultured in NB medium until logarithmic phase of growth, and then subjected to centrifugation, washed with sterile 0.9% NaCl, and normalized to an OD_600_ of 0.4, 0 or 0.366 μg/mL of dioctyldiethylenetriamine was added, and incubated for 24 h at 28°C with agitation at 60 rpm in a shaker incubator. The cultured bacteria were collected by centrifugation and fixed using a fixative (5% glutaraldehyde) for 12 h. A portion of the bacteria was dehydrated by gradient dehydration with different concentrations of ethanol, dried using a CO_2_ critical point desiccator (Leica, Germany), and the cell morphology was observed by scanning electron microscopy (Hitachi, Japan) [70].

### Swimming and swarming assays

The *Xoo* strains were cultured until mid-exponential phase in NB medium, then subjected to centrifugation, washed with sterile 0.9% NaCl, and normalized to an OD_600_ of 1.0. A serial dilution was produced by adding 0.9% sterile NaCl to the resulting bacterial solution, and then 2 μL of the bacterial solution was inoculated onto NB solid plates (Medium containing 0.3% (w/v) agar for the swimming assay; Medium containing 0.6% (w/v) agar was used for the swarming assays) containing 0 or 0.183 μg/mL dioctyldiethylenetriamine [12]. The plates were incubated at 28°C for 1–2 days, after which the colony diameters were analyzed.

### Determination of effective concentration of 50% inhibition (EC_50_) of bactericides against bacteria

The antibacterial activity of bactericides (dioctyldiethylenetriamine, kasugamycin or zinc thiazole) or bactericide mixtures (dioctyldiethylenetriamine: kasugamycin = 1:9 or dioctyldiethylenetriamine: zinc thiazole = 1:9) against bacteria *in vitro* was determined by classical turbidimetric method [2]. Bacteria were grown in NB/LB medium at 28°C and shaken at 180 rpm until an optical density at 600 nm (OD_600_) = 0.5. Subsequently, bacteria were inoculated into NB or LB medium containing bactericide or bactericide mixtures with different concentrations, respectively, which then were grown at 28°C for shaking culture at 180 rpm. The equal amount of solvent (ratio of bacterial solution:medium = 1:99, v:v) was regarded as control. The OD_600_ values were measured using a microplate reader and the EC_50_ values were calculated by regressing the percentage of growth inhibition against the log-transformed bactericide concentration. The synergistic ratio of bactericide mixtures was calculated according to Wadley formulas [40,41]. EC_50(th)_, the theoretical EC_50_ values of the mixtures were calculated as follows: EC_50(th)_ = (a + b) / (a / EC_50(A)_ + b / EC_50(B)_), where A, B represent the individual components, and a, b represents the ratios of these components in the mixtures. Synergy ratio (SR) was calculated as follows: SR = EC_50(th)_ / EC_50(ob)_, where EC_50(ob)_ are the observed EC_50_ values of the mixtures. SR < 0.5 indicates antagonistic interactions between the two bactericides in the mixture, 0.5 ≤ SR ≤ 1.5 indicates additive interactions, whereas SR > 1.5 indicates synergistic interactions.

### Acyl-homoserine lactone (AHL) bioassay

An effective AHL bioassay strain of *Agrobacterium tumefaciens* JZA1 was used to determine AHL production by the β-galactosidase method. JZA1 was grown in LB medium at 28°C with shaking at 180 rpm until an optical density at 600 nm (OD_600_) = 0.5. Fresh AT medium was added with 1% and 8% addition of JZA1 and the supernatant of the strain was tested, respectively, and the resulting cultures were incubated at 28°C and 180 rpm with shaking until the OD_600_ reached 0.4-0.8. 200 μL of culture was added to 800 μL Z-Buffer (8.5 g/L Na_2_HPO_4_, 5.5 g/L NaH_2_PO_4_·H_2_O, 0.75 g/L KCl, 0.246 g/L MgSO_4_·7H_2_O, and 2.7 mL/L β-mercaptoethanol) in a 2-mL centrifuge tube supplemented with 10 μL 0.5% SDS and 15 μL chloroform. After spinning for 20 s, 100 μL of the chromogenic substrate ONPG (2-Nitrophenyl β-D-galactopyranoside) at a concentration of 3 mg/mL was added and this time was recorded as T1. Once the reaction mixture turned yellow, 600 μL of 1M Na_2_CO_3_ was immediately added to terminate the reaction, and this time was recorded as T2. The solution was centrifuged before measuring the optical density of the supernatant at 420 nm (OD_420_). AHL activity was quantified as β-galactosidase activity / (Miller units) and expressed by the formula 1000 × OD_420_ / OD_600_ (T2-T1) / 0.2 [59].

### Phylogenetic analysis and sequence alignment

Amino acid sequence of RpfG protein from different strains was downloaded from the National Center for Biotechnology Information (NCBI) database for phylogenetic reconstruction and sequence alignment, and the amino acid sequence information used in this study was listed in S9 Table. Phylogenetic tree was generated using MEGA 6.0 software with the neighbor-joining (NJ) algorithm based on full-length amino acid sequences. Bootstrap analysis with 1000 replicates was used to assess the importance of nodes, and a p-distance model was used to ensure that divergent domains could contribute to the topology of the NJ tree [2]. Sequence alignment was carried out using DNAMAN software.

### Bacterial competition assay

For *in vitro* competition assay of phyllosphere bacteria, different strains were cultured in NB medium containing appropriate antibiotics until the mid-exponential stage. Then, the bacteria were centrifuged at 6000 rpm and resuspended in fresh NB medium to prepare bacterial suspensions with OD_600_ of 1.0. *Aaa*, which causes bacterial leaf streak of rice, *Pseudomonas syringae* pv. *syringae* (*Pss*), which causes rice brown spot, and *Pseudomonas* spp. (MSZFGNb, a rice endophytic bacterium), were used as competing strains, and the competing strains and different *Xoo* strains (with Gm resistance) were mixed in the ratios of *Xoo*:*Aaa* = 2:1, *Xoo*:*Pss* = 1:2, and *Xoo*:MSZFGNb = 1:1, respectively. Then 5 μL of the mixed bacterial solution and 2.5 μL of the unmixed bacterial solution were spotted on NA medium, respectively, and incubated at 28 °C. After 24 hours of incubation, the bacteria were resuspended in 1 mL of NB, followed by a 10-fold gradient dilution, and the diluted bacterial solution was spread on NA medium containing Gm antibiotic. The plates were incubated at 28 °C until single colonies grew and were counted.

For *in vivo* competition assay of phyllosphere bacteria, different strains were grown overnight in NB medium containing appropriate antibiotics, and the bacteria were centrifuged at 6000 rpm and resuspended in fresh NB medium to prepare bacterial suspensions with OD_600_ of 1.0. *Aaa*, *Pss*, and MSZFGNb were used as competitors. The competitors and different *Xoo* strains (with Gm resistance) were mixed in the ratios of *Xoo*:*Aaa* = 2:1, *Xoo*:*Pss* = 1:2, and *Xoo*:MSZFGNb = 1:1, respectively. The mixed and unmixed bacterial solutions were inoculated onto rice (40-day-old) leaves by leaf-cutting method, respectively. Disease progression was assessed 14 days after inoculation, and 1 cm^2^ of rice leaves were cut from the disease-health junction, sterilized with 75% ethanol for 30 s, added to 1 mL of NB medium, and crushed in a ball mill. The suspension was spread on NA medium containing Gm antibiotic and counted after single colonies grew.

## Supporting information

S1 Fig Differential genes identified based on transposon sequencing. The number of differential genes was identified by increased (up) and decreased (down) transposon insertion abundance after treatment with dioctyldiethylenetriamine.
(TIF)

S2 Fig OD_600_ values of bacteria under different treatment conditions. Sample size *n* = 3. Bar graphs denote mean ± SD. Error bars indicate SD. Results were analyzed using one-way ANOVA followed by Tukey’s multiple range test, with “ns” stands for not statistically significant.
(TIF)

S3 Fig Detection of diffuser signal factor (DSF) content. DSF content of quorum sensing signal molecules in PXO99A treated with dioctyldiethylenetriamine was determined. Sample size *n* = 3. Violin plots denote mean ± SD. Error bars indicate SD. Results were analyzed using one-way ANOVA followed by Tukey’s multiple range test, with “ns” stands for not statistically significant.
(TIF)

S4 Fig The effect of dioctyldiethylenetriamine on the content of GFP protein. (A) Effect of different concentrations of dioctyldiethylenetriamine on the expression of GFP protein in strain GFP-PXO99A. The band intensities were quantified and analyzed using ImageJ, with numbers representing the relative intensities of the corresponding bands. Coomassie brilliant blue (CBB) staining was employed as a loading control to verify the equal amounts of protein across the gel. (B) Changes in fluorescent intensity of strain GFP-PXO99A after treatment with different concentrations of dioctyldiethylenetriamine. Photographs were taken at 488 nm using a scanning confocal laser microscopy. Scale bar = 25 μm. The intensity of fluorescence was analyzed using ImageJ software. Error bars indicate SD. Results were analyzed using one-way ANOVA followed by Tukey’s multiple range test, with different letters above the figures indicating statistically significant difference at *P* < 0.05, while the same letters stand for not statistically significant.
(TIF)

S5 Fig Detection of c-di-GMP content. The content of intracellular c-di-GMP in Δ*rpfG*(*yhjH*) strain treated with dioctyldiethylenetriamine was detected. Sample size *n* = 3. Violin plots denote mean ± SD. Error bars indicate SD. Results were analyzed using one-way ANOVA followed by Tukey’s multiple range test, with “ns” stands for not statistically significant.
(TIF)

S6 Fig The expression of *rpfG* is directly regulated by Clp. (A) Detection of the interaction between Clp protein and the *rpfG* promoter region via bacterial one-hybrid assay. (B) Diagrammatic sketch showing the processes for GUS reporter vector. T0T1 are terminators, and P_*rpfG*_ is the promoter sequence of *rpfG*. (C) The effect of c-di-GMP (16 μM) on the activity of hydrolyzing 5-Bromo-4-chloro-3-indolyl-β-D-glucuronide acid (X-gluc) in different strains. (D-E) The effect of c-di-GMP (16 μM) on the activity of hydrolyzing 4-methylumbellifery-β-D-glucuronide (MUG) in different strains. (F) The effect of dioctyldiethylenetriamine (1.464 μg/mL) on the activity of hydrolyzing X-gluc in different strains. (G-H) The effect of dioctyldiethylenetriamine (1.464 μg/mL) on the activity of hydrolyzing MUG in different strains. (I) Expression levels of *rpfG* in strains PXO99A and Δ*clp* determined by qRT-PCR. (J) Effect of different concentrations of dioctyldiethylenetriamine on the expression of RpfG-GFP protein in strain RpfG-GFP-Δ*clp*. The band intensities were quantified and analyzed using ImageJ, with numbers representing the relative intensities of the corresponding bands. Coomassie brilliant blue (CBB) staining was employed as a loading control to verify the equal amounts of protein across the gel. Results in (D, E, G, H) were analyzed using one-way ANOVA followed by Tukey’s multiple range test, with different letters above the figures indicating statistically significant difference at *P* < 0.05.
(TIF)

S7 Fig Detection of gene expression levels. The expression levels of *rpfB*, *rpfF*, and *rpfC* genes in the Δ*rpfG* strain, as well as the expression levels of *rpfB*, *rpfF*, *rpfC* and *rpfG* genes in OE-*rpfG* (treated with 2.0 μg/mL dioctyldiethylenetriamine) and PXO99A strains were detected respectively by qRT-PCR. Results are presented as mean ± SD, and error bars represent SD.
(TIF)

S8 Fig Antibacterial activity in the pot experiment. Leaf damage of rice after inoculation with different strains and spraying with different concentrations of dioctyldiethylenetriamine for 14 days.
(TIF)

S9 Fig *In vitro* antibacterial activity. Growth of different strains in the presence of 0 or 1.6 μg/ml of dioctyldiethylenetriamine.
(TIF)

S10 Fig Growth assay. Growth curves of bacteria under different concentrations of dioctyldiethylenetriamine treatment (0 or 0.183 μg/mL). Sample size *n* = 3.
(TIF)

S11 Fig Cell morphology of bacterial changes in response to dioctyldiethylenetriamine by scanning electronic microscopy. The concentrations of dioctyldiethylenetriamine in the control group and treatment group were 0 and 0.366 μg/ml, respectively. Scale bar = 1 μm. The arrows indicate the location of cells exhibiting morphological changes.
(TIF)

S12 Fig Determination of cyclic dimeric GMP (c-di-GMP) content. Effect of dioctyldiethylenetriamine (4 times EC_50_ value) treatment on c-di-GMP content in *Xanthomonas oxyzae* pv. *oryzicola* (*Xoc*), *Xanthomonas campestris* pv. *campestris* (*Xcc*), *Xanthomonas citri* pv. *citri* (*Xac*), *Xanthomonas vesicatoria* (*Xve*), *Stenotrophomonas maltophilia* (*Sm*), *Agrobacterium tumefaciens* (*At*), *Pectobacterium carotovorum* subsp. *carotovorum* (*Pcc*), *Ralstonia solanacearum* (*Rs*), *Acidovorax citrulli* (*Ac*), and *Pseudomonas syringae* pv. *syringae* (*Pss*) strains. Sample size *n* = 3. Bar graphs denote mean ± SD. Error bars indicate SD. Results were analyzed using one-way ANOVA followed by Tukey’s multiple range test, with “*” stands for statistically significant difference at *P* < 0.05, “ns” stands for not statistically significant.
(TIF)

S13 Fig Determination of acyl-homoserine lactone (AHL) content. Effect of dioctyldiethylenetriamine (4 times EC_50_ value) treatment on the production of AHL in *Pectobacterium carotovorum* subsp. *carotovorum* (*Pcc*), *Ralstonia solanacearum* (*Rs*), *Acidovorax citrulli* (*Ac*), *Pseudomonas syringae* pv. *syringae* (*Pss*), and *Agrobacterium tumefaciens* (*At*) strains was indicated by the activity of β-galactosidase. Sample size *n* = 3. Bar graphs denote mean ± SD. Error bars indicate SD. Results were analyzed using one-way ANOVA followed by Tukey’s multiple range test, with “ns” stands for not statistically significant.
(TIF)

S14 Fig Amino acid sequence alignment of RpfG proteins from different strains. Abbreviations: *Xoo*, *Xanthomonas oryzae* pv. *oryzae*; *Xoc*, *Xanthomonas oryzae* pv. *oryzicola*; *Xcc*, *Xanthomonas campestris* pv. *campestris*; *Xac*, *Xanthomonas citri* pv. *citri*; *Xve*, *Xanthomonas vesicatoria*; *Sm*, *Stenotrophomonas maltophilia*.
(TIF)

S15 Fig Expression levels of genes related to the T6SS system in different strains treated with dioctyldiethylenetriamine (4 times EC_50_ value). Numbers on the heatmap indicating relative gene expression levels were calculated using the log_2_2^-ΔΔCT^ method. The results of the same gene under different treatments were analyzed using one-way ANOVA followed by Tukey’s multiple range test, with different letters next to the numbers indicate statistically significant difference at *P* < 0.05.
(TIF)

S1 Table Changes in transposon insertion abundance of 34 genes in the quorum sensing system following dioctyldiethylenetriamine treatment. (DOCX)

S2 Table Growth curves of bacteria under different treatment conditions. (DOCX)

S3 Table The *in vitro* antibacterial activity of dioctyldiethylenetriamine and dioctyldiethylenetriamine mixtures. (DOCX)

S4 Table Strains used in this study. (DOCX)

S5 Table Primers used in this study. (DOCX)

S6 Table Plasmids used in this study. (DOCX)

S7 Table Kyoto Encyclopedia of Genes and Genomes (KEGG) pathway enrichment analysis of differentially genes. (DOCX)

S8 Table Distribution of gene encoding protein of the RpfG (KO term: K13815) within the KEGG database. (DOCX)

S9 Table Phylogenetic relationship of RpfG in 100 bacteria. (DOCX)
