# Supplementary figures and images for: A chemical bactericide dioctyldiethylenetriamine (Xinjunan) exerts a non-lethal effect by inhibiting RpfG activity to regulate the quorum sensing system
Source: PLoS Pathog. 2026 Jun 10;22(6):e1014320. doi: 10.1371/journal.ppat.1014320 (PMC13274925; doi:10.1371/journal.ppat.1014320)

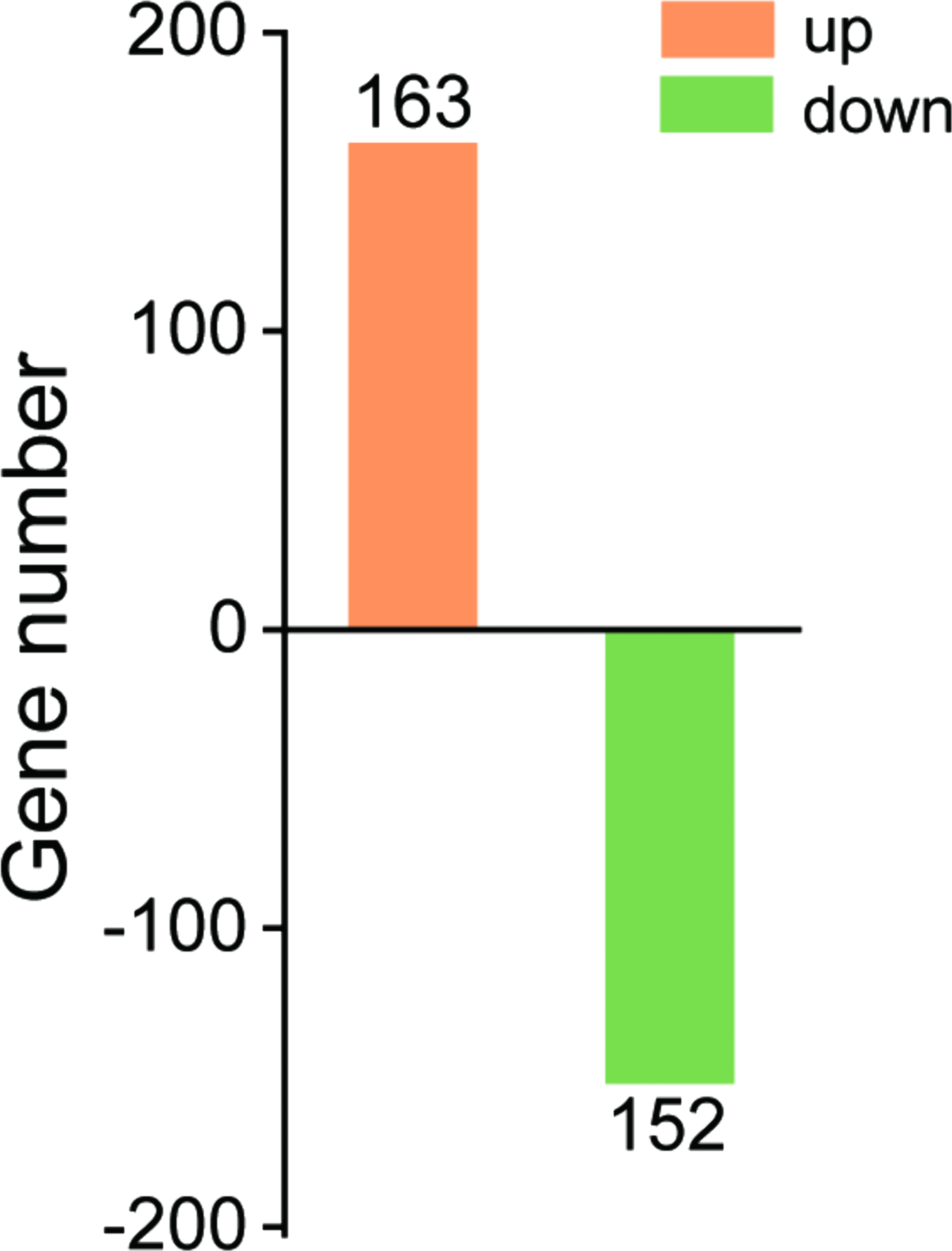

Supplement: S1 Fig — The number of differential genes was identified by increased (up) and decreased (down) transposon insertion abundance after treatment with dioctyldiethylenetriamine. (TIF) [file ppat.1014320.s001.tif]

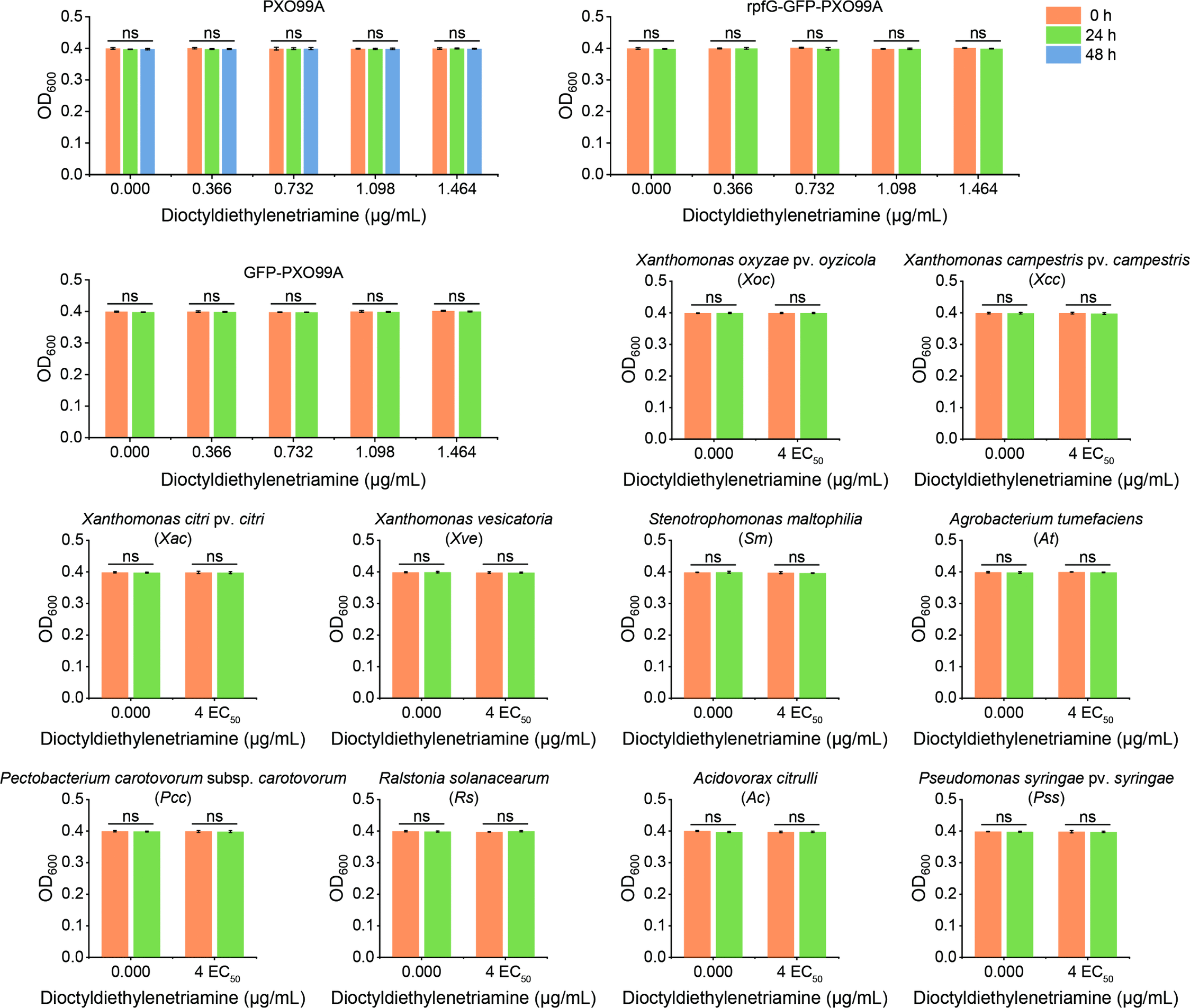

Supplement: S2 Fig — Sample size n = 3. Bar graphs denote mean ± SD. Error bars indicate SD. Results were analyzed using one-way ANOVA followed by Tukey’s multiple range test, with “ns” stands for not statistically significant. (TIF) [file ppat.1014320.s002.tif]

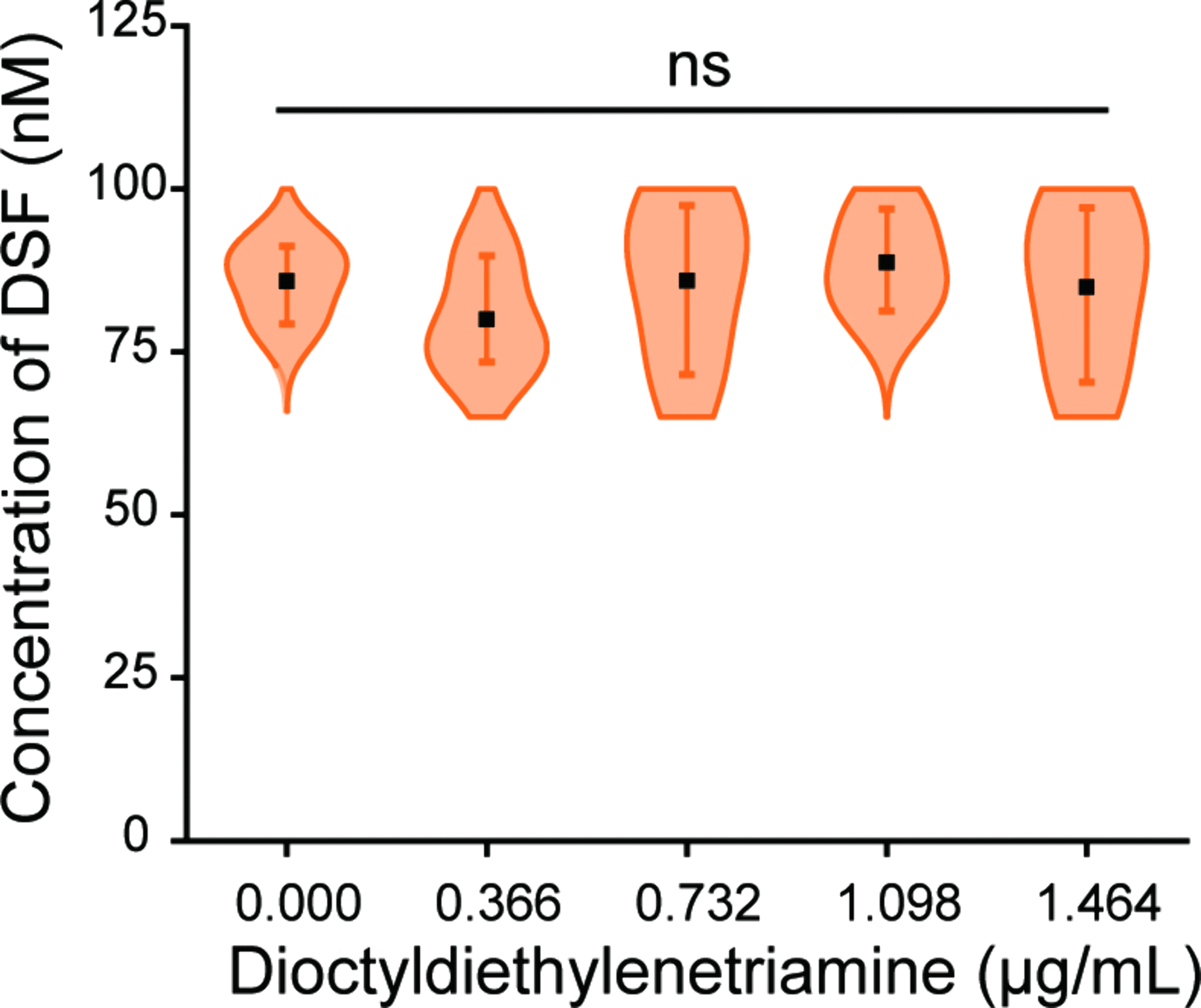

Supplement: S3 Fig — DSF content of quorum sensing signal molecules in PXO99A treated with dioctyldiethylenetriamine was determined. Sample size n = 3. Violin plots denote mean ± SD. Error bars indicate SD. Results were analyzed using one-way ANOVA followed by Tukey’s multiple range test, with “ns” stands for not statistically significant. (TIF) [file ppat.1014320.s003.tif]

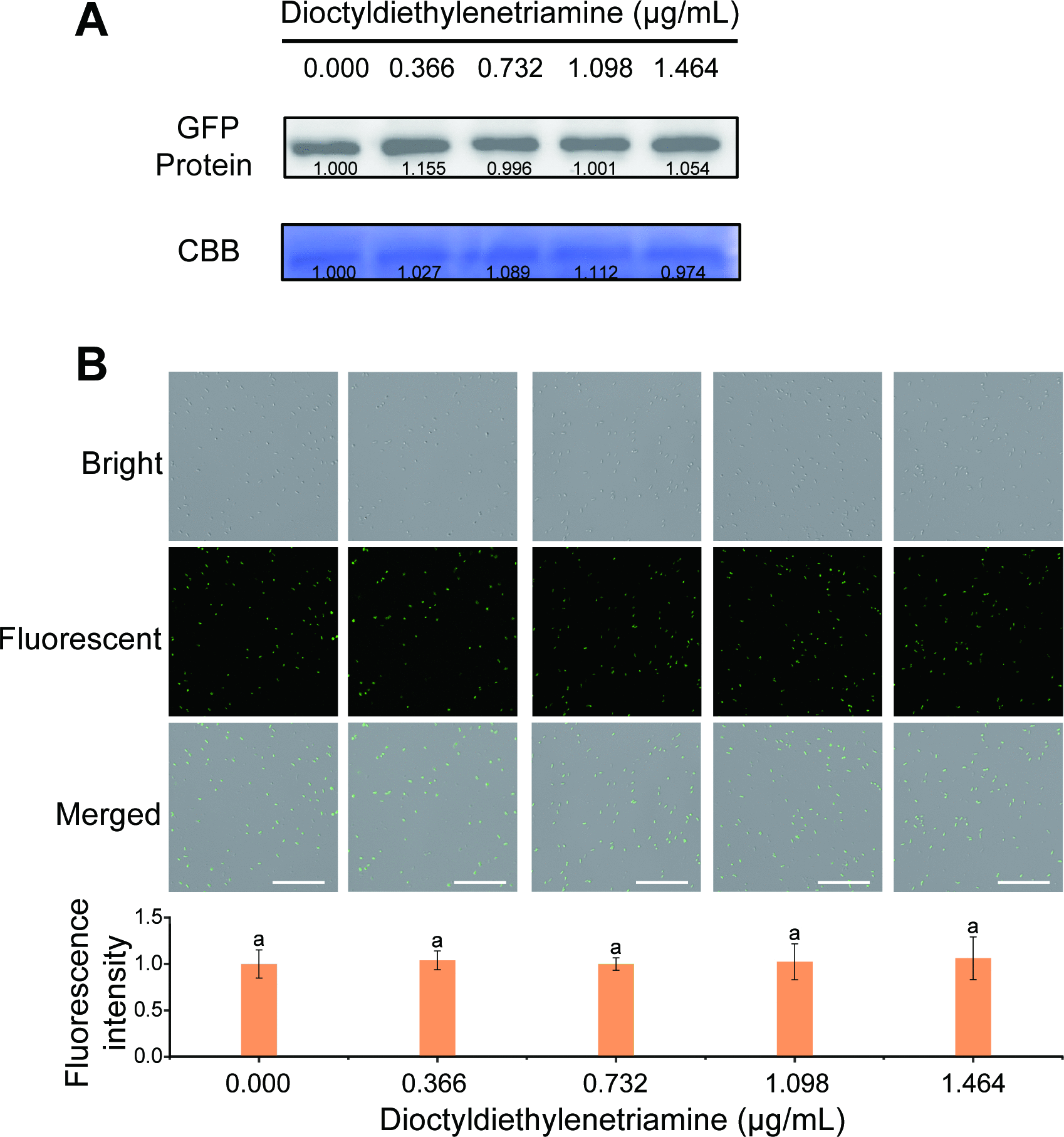

Supplement: S4 Fig — (A) Effect of different concentrations of dioctyldiethylenetriamine on the expression of GFP protein in strain GFP-PXO99A. The band intensities were quantified and analyzed using ImageJ, with numbers representing the relative intensities of the corresponding bands. Coomassie brilliant blue (CBB) staining was employed as a loading control to verify the equal amounts of protein across the gel. (B) Changes in fluorescent intensity of strain GFP-PXO99A after treatment with different concentrations of dioctyldiethylenetriamine. Photographs were taken at 488 nm using a scanning confocal laser microscopy. Scale bar = 25 μm. The intensity of fluorescence was analyzed using ImageJ software. Error bars indicate SD. Results were analyzed using one-way ANOVA followed by Tukey’s multiple range test, with different letters above the figures indicating statistically significant difference at P < 0.05, while the same letters stand for not statistically significant. (TIF) [file ppat.1014320.s004.tif]

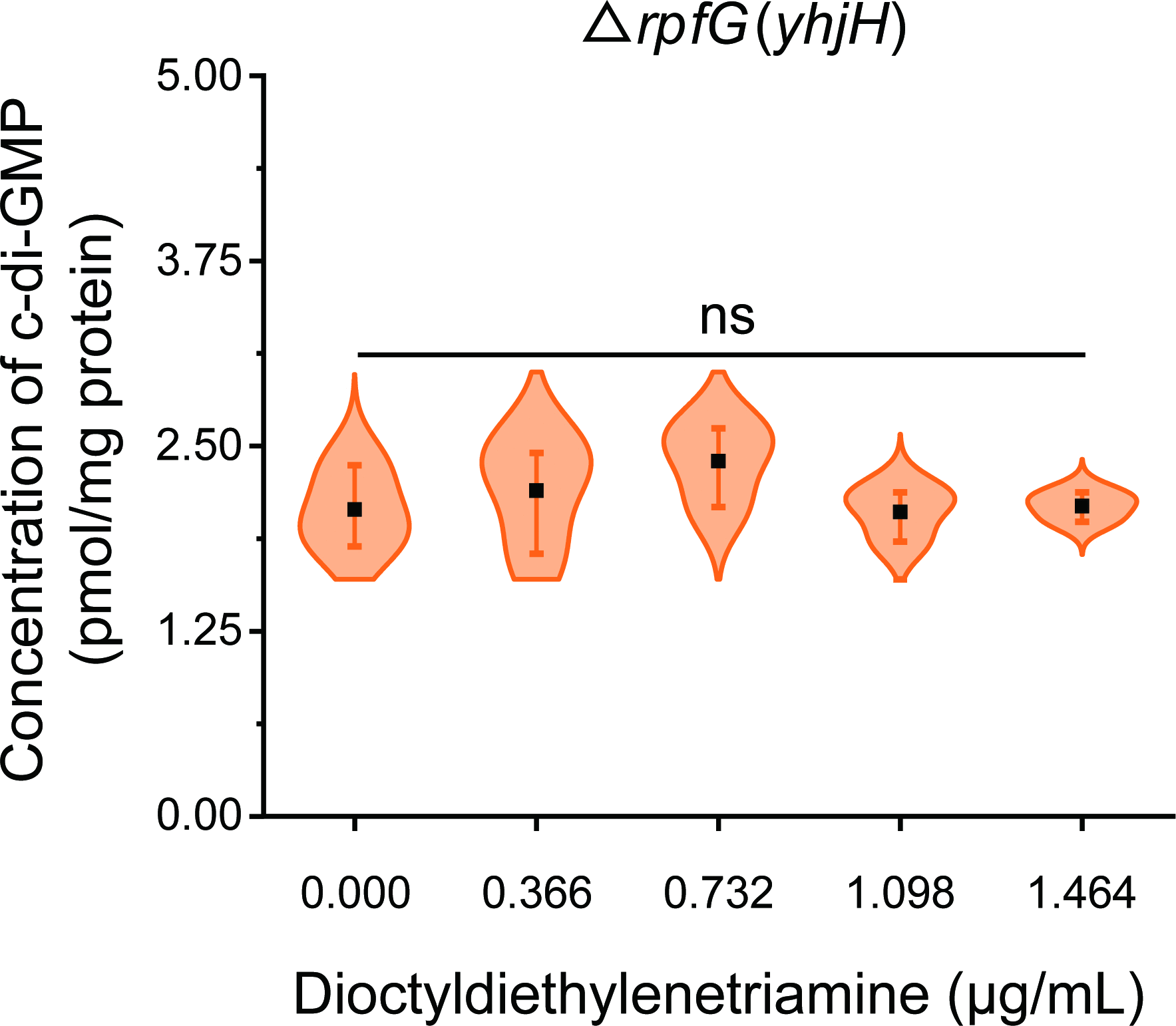

Supplement: S5 Fig — The content of intracellular c-di-GMP in ΔrpfG(yhjH) strain treated with dioctyldiethylenetriamine was detected. Sample size n = 3. Violin plots denote mean ± SD. Error bars indicate SD. Results were analyzed using one-way ANOVA followed by Tukey’s multiple range test, with “ns” stands for not statistically significant. (TIF) [file ppat.1014320.s005.tif]

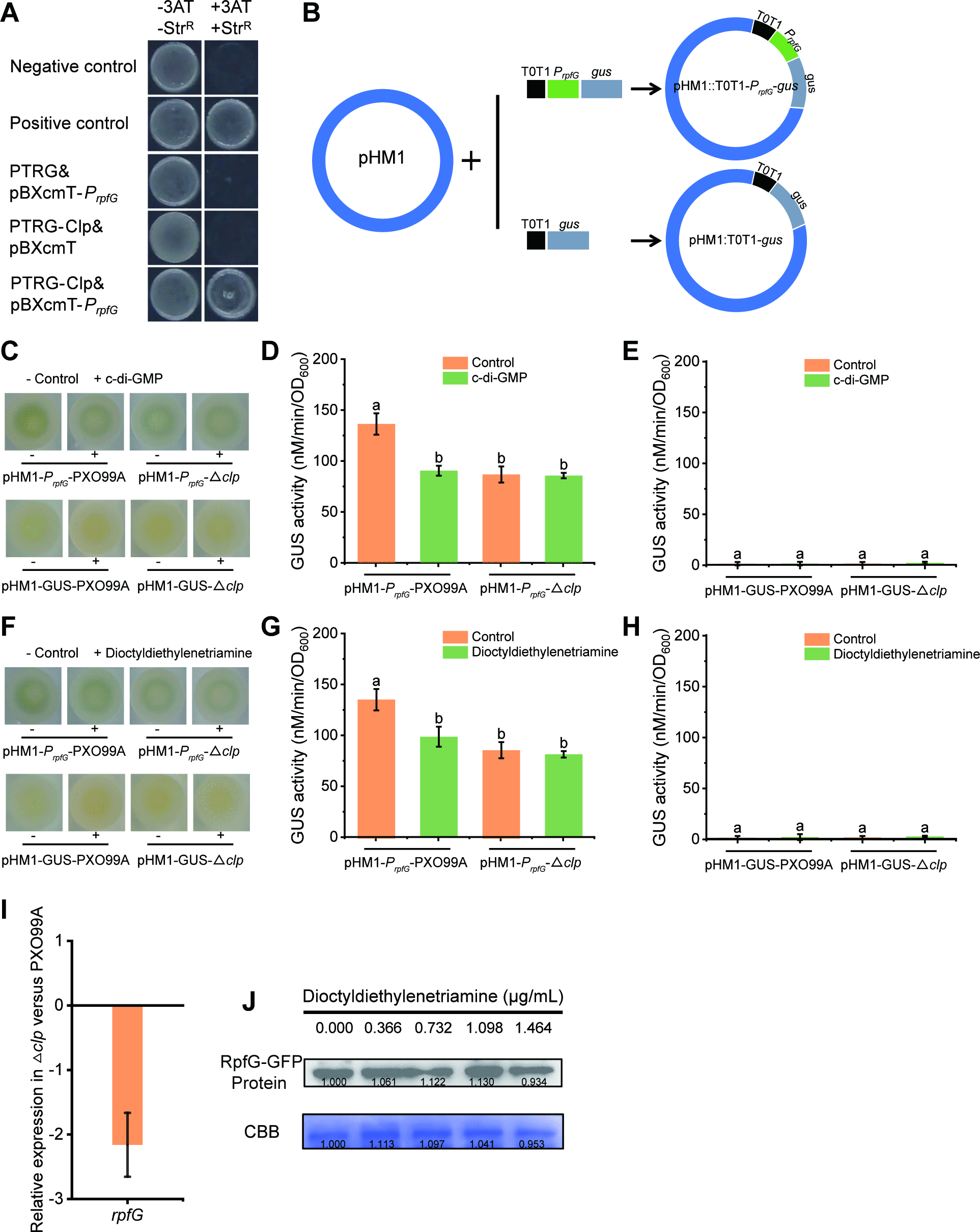

Supplement: S6 Fig — (A) Detection of the interaction between Clp protein and the rpfG promoter region via bacterial one-hybrid assay. (B) Diagrammatic sketch showing the processes for GUS reporter vector. T0T1 are terminators, and PrpfG is the promoter sequence of rpfG. (C) The effect of c-di-GMP (16 μM) on the activity of hydrolyzing 5-Bromo-4-chloro-3-indolyl-β-D-glucuronide acid (X-gluc) in different strains. (D-E) The effect of c-di-GMP (16 μM) on the activity of hydrolyzing 4-methylumbellifery-β-D-glucuronide (MUG) in different strains. (F) The effect of dioctyldiethylenetriamine (1.464 μg/mL) on the activity of hydrolyzing X-gluc in different strains. (G-H) The effect of dioctyldiethylenetriamine (1.464 μg/mL) on the activity of hydrolyzing MUG in different strains. (I) Expression levels of rpfG in strains PXO99A and Δclp determined by qRT-PCR. (J) Effect of different concentrations of dioctyldiethylenetriamine on the expression of RpfG-GFP protein in strain RpfG-GFP-Δclp. The band intensities were quantified and analyzed using ImageJ, with numbers representing the relative intensities of the corresponding bands. Coomassie brilliant blue (CBB) staining was employed as a loading control to verify the equal amounts of protein across the gel. Results in (D, E, G, H) were analyzed using one-way ANOVA followed by Tukey’s multiple range test, with different letters above the figures indicating statistically significant difference at P < 0.05. (TIF) [file ppat.1014320.s006.tif]

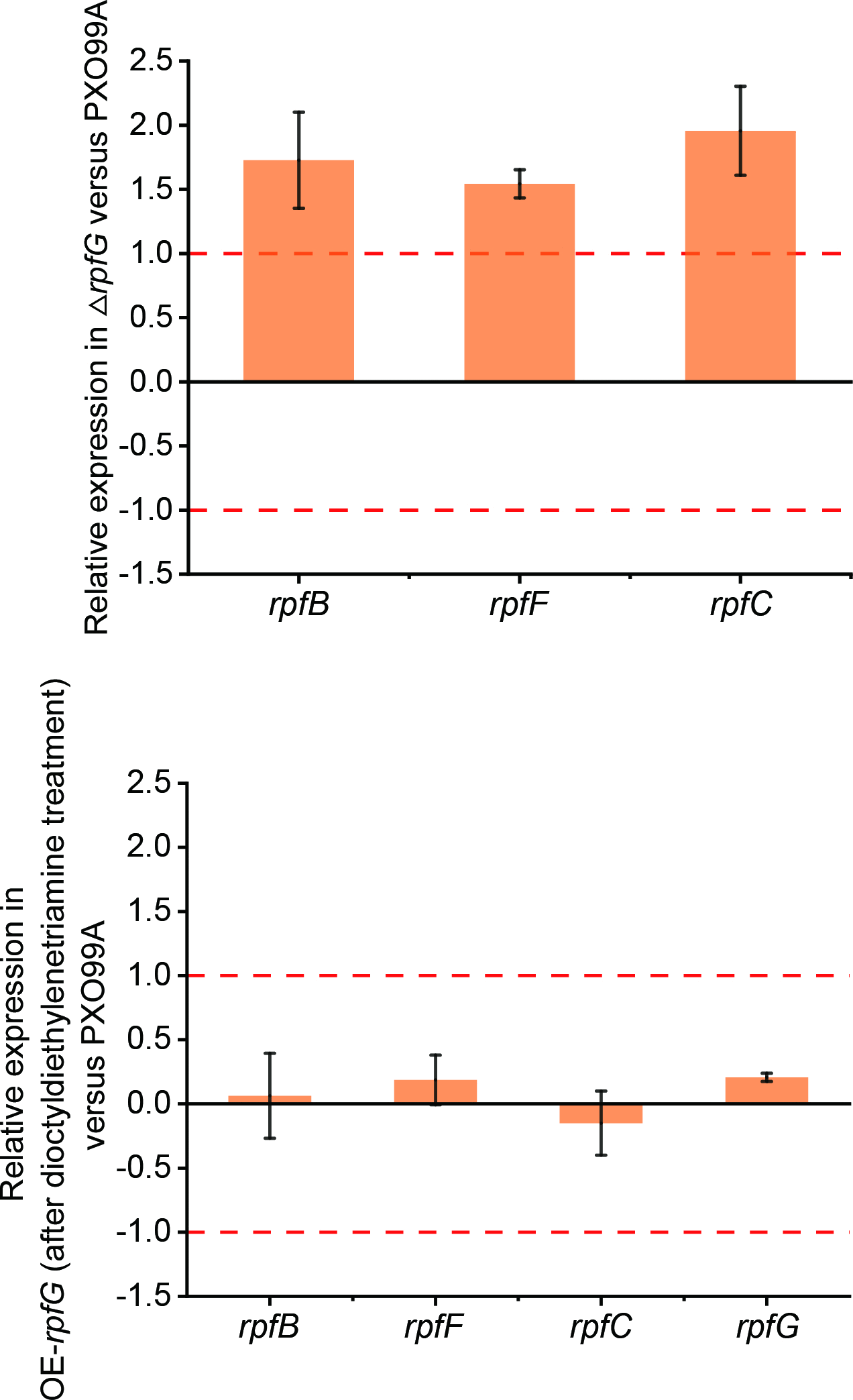

Supplement: S7 Fig — The expression levels of rpfB, rpfF, and rpfC genes in the ΔrpfG strain, as well as the expression levels of rpfB, rpfF, rpfC and rpfG genes in OE-rpfG (treated with 2.0 μg/mL dioctyldiethylenetriamine) and PXO99A strains were detected respectively by qRT-PCR. Results are presented as mean ± SD, and error bars represent SD. (TIF) [file ppat.1014320.s007.tif]

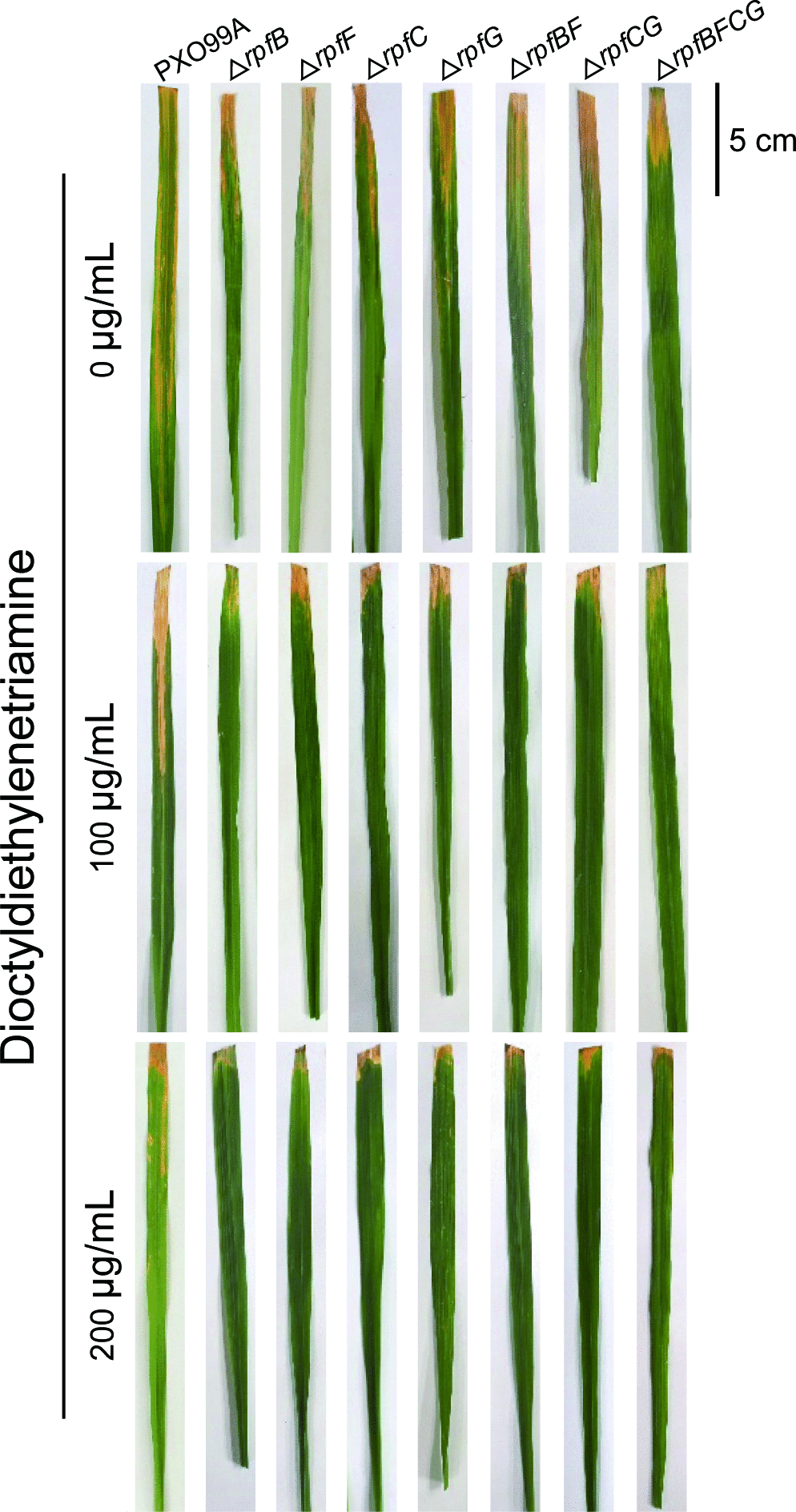

Supplement: S8 Fig — Leaf damage of rice after inoculation with different strains and spraying with different concentrations of dioctyldiethylenetriamine for 14 days. (TIF) [file ppat.1014320.s008.tif]

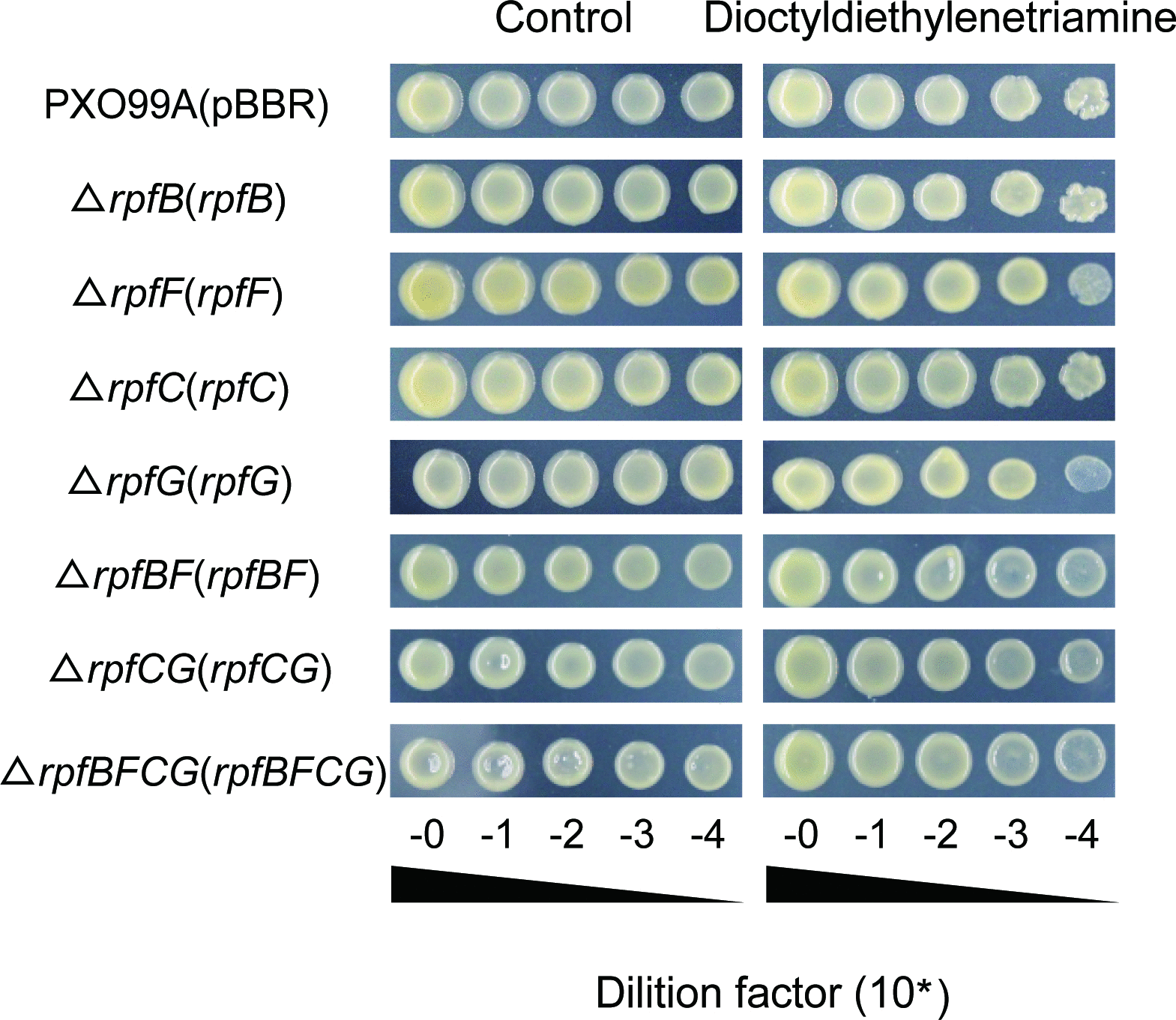

Supplement: S9 Fig — Growth of different strains in the presence of 0 or 1.6 μg/ml of dioctyldiethylenetriamine. (TIF) [file ppat.1014320.s009.tif]

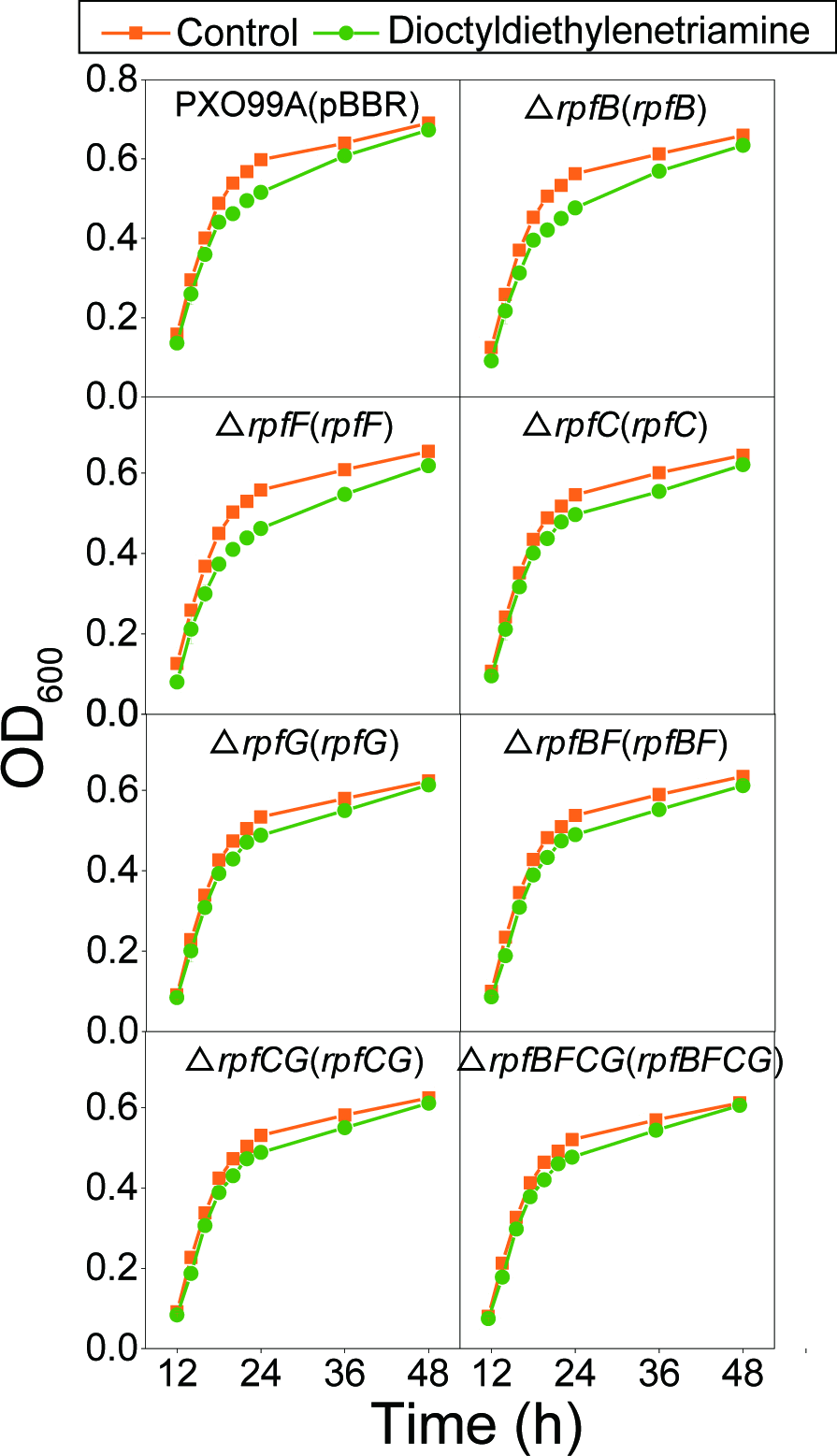

Supplement: S10 Fig — Growth curves of bacteria under different concentrations of dioctyldiethylenetriamine treatment (0 or 0.183 μg/mL). Sample size n = 3. (TIF) [file ppat.1014320.s010.tif]

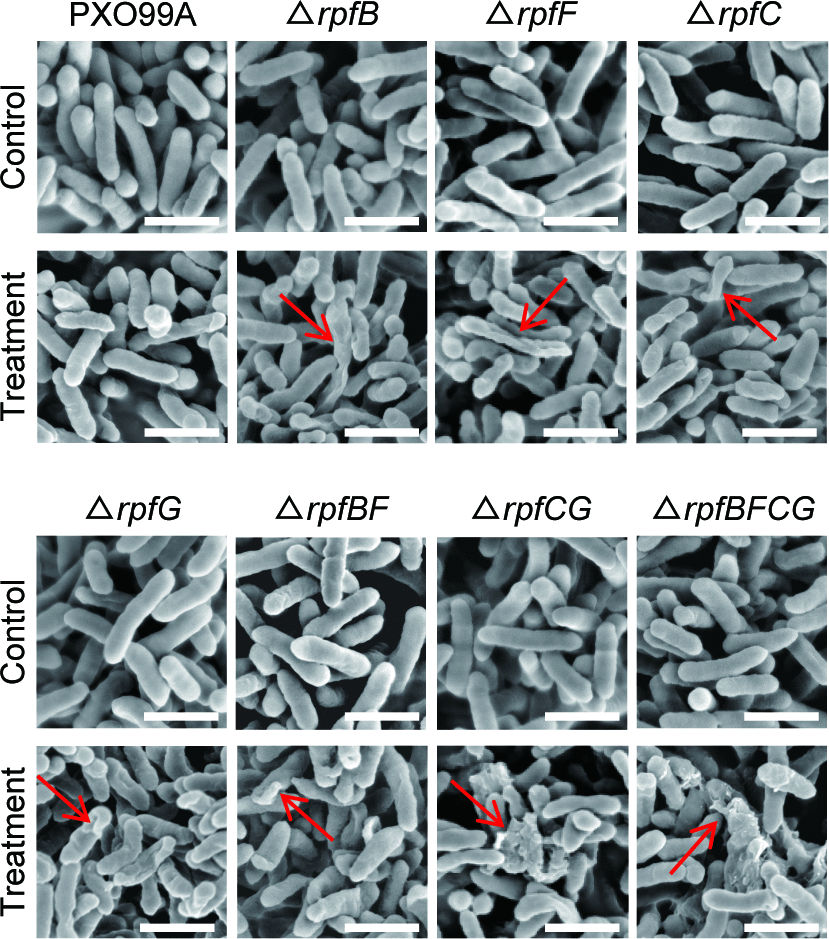

Supplement: S11 Fig — The concentrations of dioctyldiethylenetriamine in the control group and treatment group were 0 and 0.366 μg/ml, respectively. Scale bar = 1 μm. The arrows indicate the location of cells exhibiting morphological changes. (TIF) [file ppat.1014320.s011.tif]

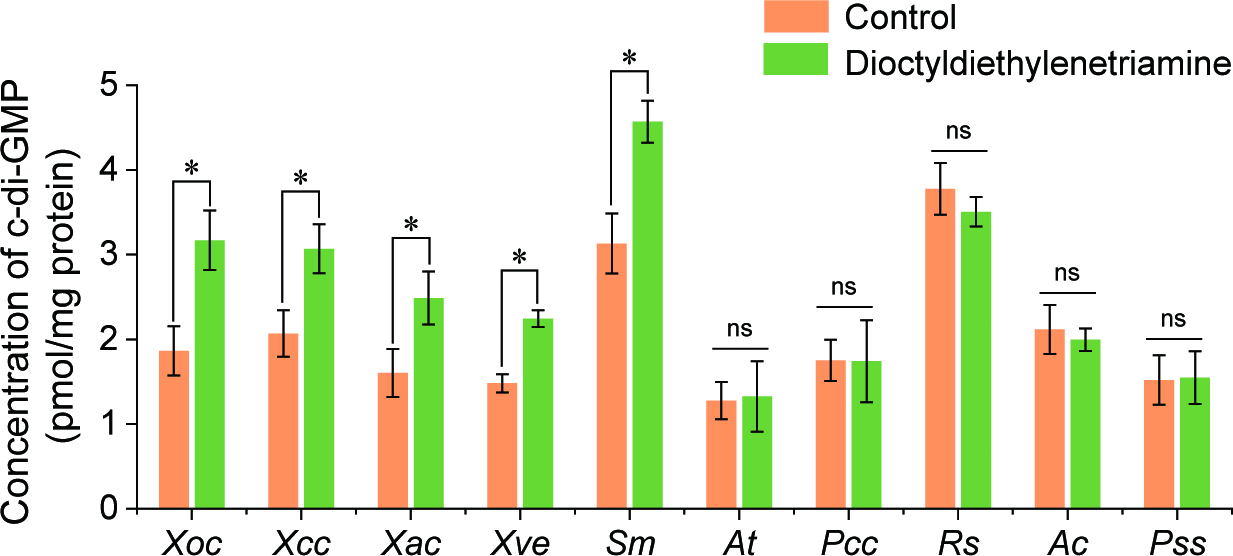

Supplement: S12 Fig — Effect of dioctyldiethylenetriamine (4 times EC50 value) treatment on c-di-GMP content in Xanthomonas oxyzae pv. oryzicola (Xoc), Xanthomonas campestris pv. campestris (Xcc), Xanthomonas citri pv. citri (Xac), Xanthomonas vesicatoria (Xve), Stenotrophomonas maltophilia (Sm), Agrobacterium tumefaciens (At), Pectobacterium carotovorum subsp. carotovorum (Pcc), Ralstonia solanacearum (Rs), Acidovorax citrulli (Ac), and Pseudomonas syringae pv. syringae (Pss) strains. Sample size n = 3. Bar graphs denote mean ± SD. Error bars indicate SD. Results were analyzed using one-way ANOVA followed by Tukey’s multiple range test, with “*” stands for statistically significant difference at P < 0.05, “ns” stands for not statistically significant. (TIF) [file ppat.1014320.s012.tif]

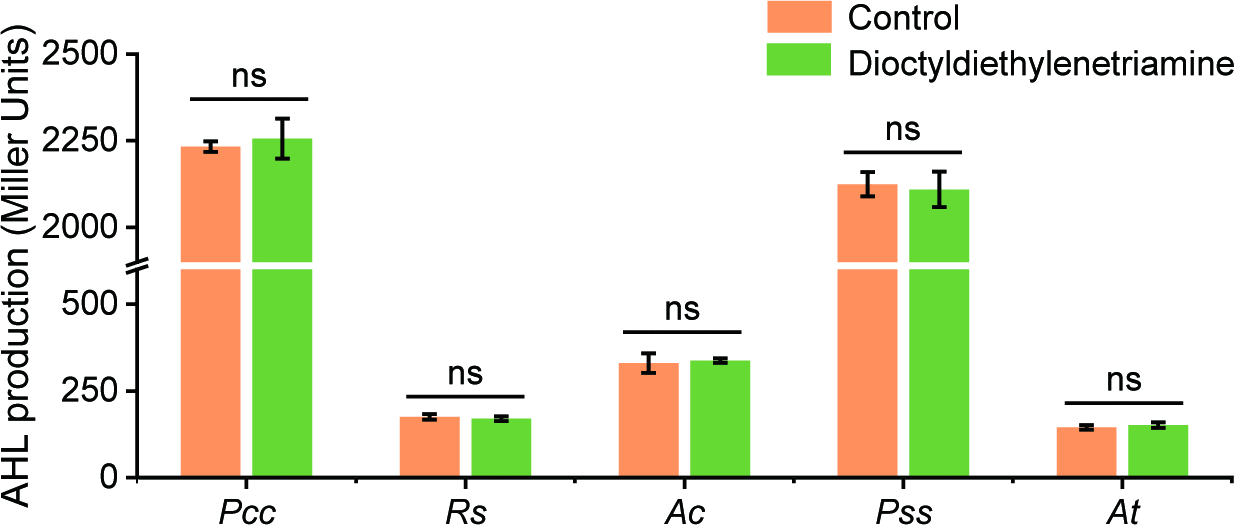

Supplement: S13 Fig — Effect of dioctyldiethylenetriamine (4 times EC50 value) treatment on the production of AHL in Pectobacterium carotovorum subsp. carotovorum (Pcc), Ralstonia solanacearum (Rs), Acidovorax citrulli (Ac), Pseudomonas syringae pv. syringae (Pss), and Agrobacterium tumefaciens (At) strains was indicated by the activity of β-galactosidase. Sample size n = 3. Bar graphs denote mean ± SD. Error bars indicate SD. Results were analyzed using one-way ANOVA followed by Tukey’s multiple range test, with “ns” stands for not statistically significant. (TIF) [file ppat.1014320.s013.tif]

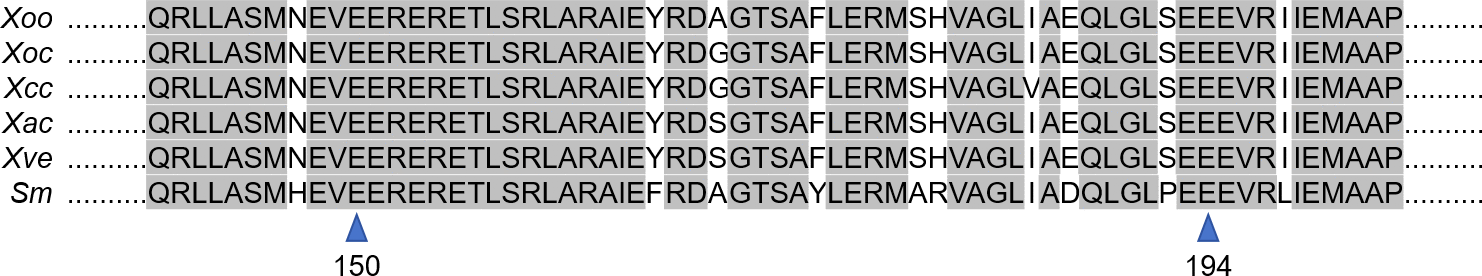

Supplement: S14 Fig — Abbreviations: Xoo, Xanthomonas oryzae pv. oryzae; Xoc, Xanthomonas oryzae pv. oryzicola; Xcc, Xanthomonas campestris pv. campestris; Xac, Xanthomonas citri pv. citri; Xve, Xanthomonas vesicatoria; Sm, Stenotrophomonas maltophilia. (TIF) [file ppat.1014320.s014.tif]

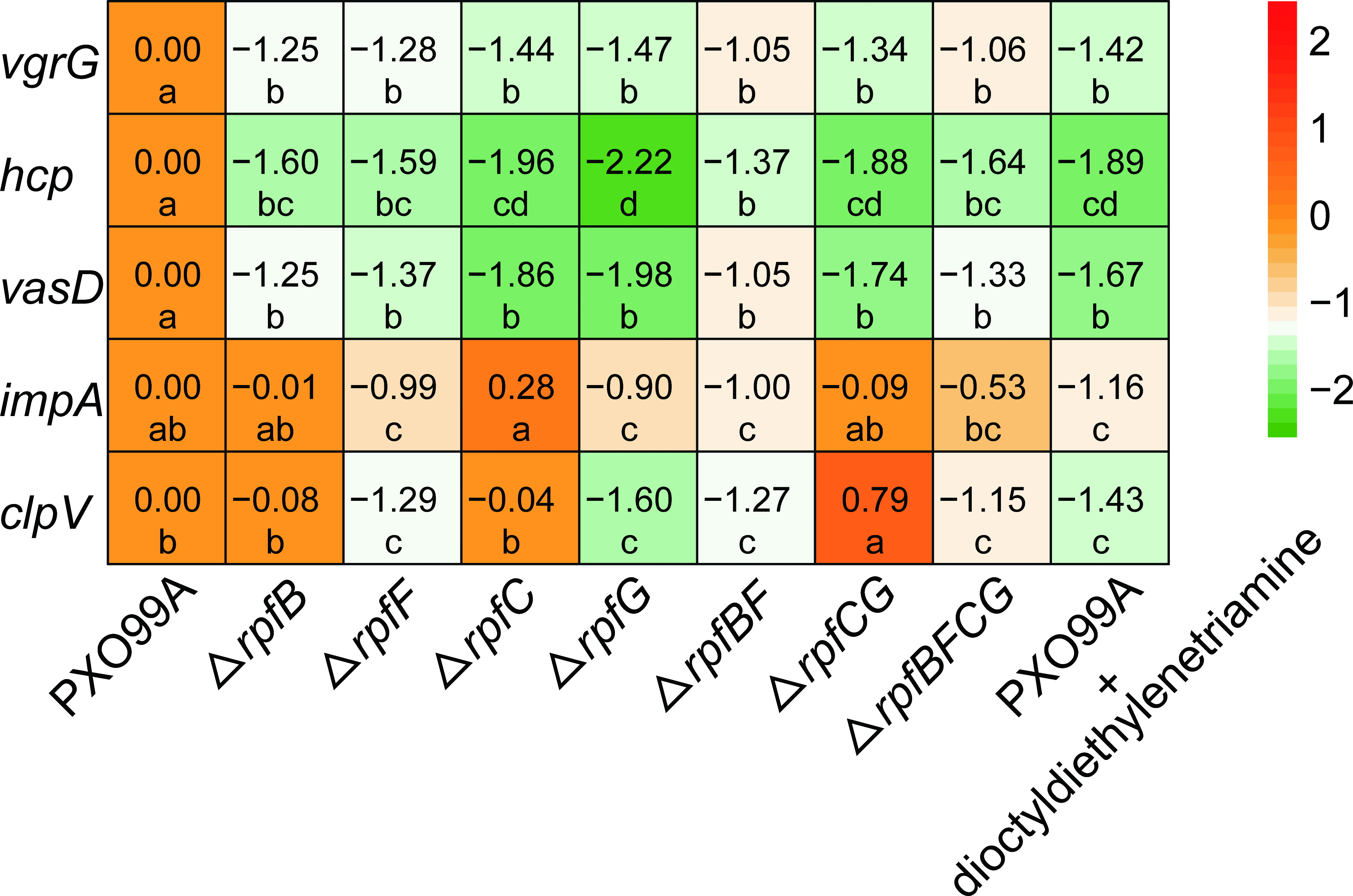

Supplement: S15 Fig — Numbers on the heatmap indicating relative gene expression levels were calculated using the log22-ΔΔCT method. The results of the same gene under different treatments were analyzed using one-way ANOVA followed by Tukey’s multiple range test, with different letters next to the numbers indicate statistically significant difference at P < 0.05. (TIF) [file ppat.1014320.s015.tif]
