# Supplementary material for: A chemical bactericide dioctyldiethylenetriamine (Xinjunan) exerts a non-lethal effect by inhibiting RpfG activity to regulate the quorum sensing system
Source: PLoS Pathog. 2026 Jun 10;22(6):e1014320. doi: 10.1371/journal.ppat.1014320 (PMC13274925; doi:10.1371/journal.ppat.1014320)
Supplement: S1 Table — (DOCX) [file ppat.1014320.s016.docx]

**S1 Table.** Changes in transposon insertion abundance of 34 genes in the quorum sensing system following dioctyldiethylenetriamine treatment.

| **Gene id** | **Log_2_FC^a^** |
| --- | --- |
| PXO_00070 | 1.37 |
| PXO_00524 | 0.00 |
| PXO_01286 | 0.00 |
| PXO_01431 | 0.00 |
| PXO_02325 | 0.00 |
| PXO_03487 | 0.00 |
| PXO_04065 | 0.00 |
| PXO_04358 | 0.00 |
| PXO_04537 | 0.00 |
| PXO_04000 | -0.14 |
| PXO_03699 | -0.44 |
| PXO_03970 | -0.56 |
| PXO_02007 | -0.65 |
| PXO_00155 | -0.65 |
| PXO_00068 | -0.68 |
| PXO_01545 | -0.72 |
| PXO_03059 | -0.73 |
| PXO_04562 | -0.73 |
| PXO_01547 | -0.73 |
| PXO_02511 | -0.75 |
| PXO_02650 | -0.77 |
| PXO_01546 | -0.78 |
| PXO_02551 | -0.80 |
| PXO_04383 | -0.80 |
| PXO_04401 | -0.89 |
| PXO_00069 | -0.99 |
| PXO_02209 | -1.00 |
| PXO_04561 | -1.05 |
| PXO_04195 | -1.06 |
| PXO_04006 | -1.17 |
| PXO_04500 | -1.44 |
| PXO_04819 | -3.04 |
| PXO_02143 | -3.64 |
| PXO_00067 | -4.09 |

^a^ Log_2_FC represents the log-fold-change of transposon insertion abundance between treated and untreated groups.
