# Supplementary material for: A chemical bactericide dioctyldiethylenetriamine (Xinjunan) exerts a non-lethal effect by inhibiting RpfG activity to regulate the quorum sensing system
Source: PLoS Pathog. 2026 Jun 10;22(6):e1014320. doi: 10.1371/journal.ppat.1014320 (PMC13274925; doi:10.1371/journal.ppat.1014320)
Supplement: S2 Table — (DOCX) [file ppat.1014320.s017.docx]

**S2 Table.** Growth curves of bacteria under different treatment conditions.

| **Time (h)** | **PXO99A** | | |
| --- | --- | --- | --- |
|  | **Control (OD_600_)** | **Dioctyldiethylenetriamine (OD_600_)** |  |
| 12 | 0.162±0.009 | 0.146±0.004 | * |
| 14 | 0.298±0.007 | 0.266±0.025 | ns |
| 16 | 0.408±0.006 | 0.369±0.010 | ** |
| 18 | 0.495±0.010 | 0.448±0.008 | ** |
| 20 | 0.543±0.009 | 0.486±0.006 | *** |
| 22 | 0.575±0.007 | 0.519±0.009 | ** |
| 24 | 0.603±0.007 | 0.544±0.009 | *** |
| 36 | 0.653±0.012 | 0.628±0.008 | * |
| 48 | 0.698±0.008 | 0.688±0.009 | ns |
| **Time (h)** | **PXO99A(pBBR)** | | |
|  | **Control (OD_600_)** | **Dioctyldiethylenetriamine (OD_600_)** |  |
| 12 | 0.163±0.004 | 0.141±0.005 | ** |
| 14 | 0.300±0.003 | 0.265±0.027 | ns |
| 16 | 0.406±0.004 | 0.364±0.014 | ** |
| 18 | 0.493±0.006 | 0.446±0.011 | ** |
| 20 | 0.544±0.007 | 0.467±0.010 | *** |
| 22 | 0.573±0.007 | 0.500±0.006 | *** |
| 24 | 0.603±0.007 | 0.521±0.007 | *** |
| 36 | 0.645±0.005 | 0.613±0.008 | ** |
| 48 | 0.696±0.003 | 0.678±0.009 | * |
| **Time (h)** | **ΔrpfB** | | |
|  | **Control (OD_600_)** | **Dioctyldiethylenetriamine (OD_600_)** |  |
| 12 | 0.128±0.025 | 0.064±0.008 | * |
| 14 | 0.255±0.012 | 0.127±0.009 | *** |
| 16 | 0.347±0.016 | 0.174±0.008 | *** |
| 18 | 0.444±0.011 | 0.220±0.010 | *** |
| 20 | 0.510±0.007 | 0.261±0.007 | *** |
| 22 | 0.549±0.007 | 0.297±0.010 | *** |
| 24 | 0.584±0.008 | 0.339±0.013 | *** |
| 36 | 0.640±0.006 | 0.494±0.015 | *** |
| 48 | 0.672±0.008 | 0.585±0.014 | *** |
| **Time (h)** | **ΔrpfB(rpfB)** | | |
|  | **Control (OD_600_)** | **Dioctyldiethylenetriamine (OD_600_)** |  |
| 12 | 0.167±0.004 | 0.133±0.004 | *** |
| 14 | 0.300±0.005 | 0.259±0.032 | ns |
| 16 | 0.412±0.005 | 0.354±0.014 | ** |
| 18 | 0.495±0.004 | 0.437±0.009 | *** |
| 20 | 0.548±0.004 | 0.463±0.008 | *** |
| 22 | 0.575±0.004 | 0.492±0.014 | *** |
| 24 | 0.604±0.005 | 0.519±0.006 | *** |
| 36 | 0.655±0.006 | 0.611±0.005 | *** |
| 48 | 0.702±0.006 | 0.676±0.008 | ** |
| **Time (h)** | **ΔrpfF** | | |
|  | **Control (OD_600_)** | **Dioctyldiethylenetriamine (OD_600_)** |  |
| 12 | 0.150±0.008 | 0.060±0.008 | *** |
| 14 | 0.258±0.006 | 0.121±0.008 | *** |
| 16 | 0.352±0.004 | 0.170±0.007 | *** |
| 18 | 0.446±0.005 | 0.216±0.010 | *** |
| 20 | 0.511±0.005 | 0.257±0.007 | *** |
| 22 | 0.552±0.005 | 0.289±0.007 | *** |
| 24 | 0.587±0.004 | 0.332±0.006 | *** |
| 36 | 0.644±0.004 | 0.463±0.008 | *** |
| 48 | 0.684±0.004 | 0.550±0.007 | *** |
| **Time (h)** | **ΔrpfF(rpfF)** | | |
|  | **Control (OD_600_)** | **Dioctyldiethylenetriamine (OD_600_)** |  |
| 12 | 0.167±0.008 | 0.120±0.009 | ** |
| 14 | 0.301±0.009 | 0.253±0.037 | ns |
| 16 | 0.412±0.008 | 0.343±0.021 | ** |
| 18 | 0.495±0.008 | 0.417±0.018 | ** |
| 20 | 0.548±0.009 | 0.454±0.010 | *** |
| 22 | 0.575±0.009 | 0.483±0.015 | *** |
| 24 | 0.604±0.011 | 0.507±0.012 | *** |
| 36 | 0.655±0.009 | 0.593±0.008 | *** |
| 48 | 0.701±0.008 | 0.665±0.010 | ** |
| **Time (h)** | **ΔrpfC** | | |
|  | **Control (OD_600_)** | **Dioctyldiethylenetriamine (OD_600_)** |  |
| 12 | 0.130±0.007 | 0.056±0.009 | *** |
| 14 | 0.234±0.007 | 0.117±0.007 | *** |
| 16 | 0.327±0.008 | 0.163±0.006 | *** |
| 18 | 0.424±0.008 | 0.213±0.013 | *** |
| 20 | 0.494±0.008 | 0.253±0.006 | *** |
| 22 | 0.534±0.008 | 0.291±0.006 | *** |
| 24 | 0.566±0.008 | 0.330±0.008 | *** |
| 36 | 0.613±0.008 | 0.465±0.007 | *** |
| 48 | 0.660±0.008 | 0.564±0.003 | *** |
| **Time (h)** | **ΔrpfC(rpfC)** | | |
|  | **Control (OD_600_)** | **Dioctyldiethylenetriamine (OD_600_)** |  |
| 12 | 0.161±0.006 | 0.150±0.012 | ns |
| 14 | 0.296±0.007 | 0.265±0.027 | ns |
| 16 | 0.406±0.004 | 0.371±0.010 | * |
| 18 | 0.489±0.006 | 0.455±0.011 | ** |
| 20 | 0.543±0.007 | 0.491±0.008 | *** |
| 22 | 0.572±0.007 | 0.532±0.004 | *** |
| 24 | 0.600±0.006 | 0.551±0.007 | *** |
| 36 | 0.655±0.010 | 0.608±0.010 | ** |
| 48 | 0.699±0.007 | 0.675±0.006 | * |
| **Time (h)** | **ΔrpfG** | | |
|  | **Control (OD_600_)** | **Dioctyldiethylenetriamine (OD_600_)** |  |
| 12 | 0.126±0.005 | 0.041±0.005 | *** |
| 14 | 0.230±0.007 | 0.094±0.010 | *** |
| 16 | 0.323±0.007 | 0.137±0.007 | *** |
| 18 | 0.411±0.005 | 0.175±0.014 | *** |
| 20 | 0.484±0.007 | 0.215±0.011 | *** |
| 22 | 0.523±0.007 | 0.250±0.009 | *** |
| 24 | 0.556±0.005 | 0.283±0.011 | *** |
| 36 | 0.599±0.006 | 0.409±0.010 | *** |
| 48 | 0.649±0.006 | 0.479±0.007 | *** |
| **Time (h)** | **ΔrpfG(rpfG)** | | |
|  | **Control (OD_600_)** | **Dioctyldiethylenetriamine (OD_600_)** |  |
| 12 | 0.155±0.012 | 0.148±0.011 | ns |
| 14 | 0.292±0.009 | 0.264±0.028 | ns |
| 16 | 0.402±0.010 | 0.372±0.011 | * |
| 18 | 0.490±0.012 | 0.456±0.013 | * |
| 20 | 0.537±0.015 | 0.493±0.008 | ** |
| 22 | 0.568±0.010 | 0.534±0.002 | ** |
| 24 | 0.597±0.010 | 0.552±0.007 | ** |
| 36 | 0.643±0.009 | 0.613±0.006 | ** |
| 48 | 0.687±0.014 | 0.677±0.004 | ns |
| **Time (h)** | **ΔrpfBF** | | |
|  | **Control (OD_600_)** | **Dioctyldiethylenetriamine (OD_600_)** |  |
| 12 | 0.118±0.004 | 0.047±0.010 | *** |
| 14 | 0.221±0.006 | 0.108±0.013 | *** |
| 16 | 0.312±0.007 | 0.142±0.010 | *** |
| 18 | 0.396±0.006 | 0.180±0.008 | *** |
| 20 | 0.464±0.005 | 0.223±0.013 | *** |
| 22 | 0.501±0.009 | 0.260±0.007 | *** |
| 24 | 0.539±0.005 | 0.303±0.009 | *** |
| 36 | 0.595±0.008 | 0.435±0.010 | *** |
| 48 | 0.652±0.010 | 0.495±0.006 | *** |
| **Time (h)** | **ΔrpfBF(rpfBF)** | | |
|  | **Control (OD_600_)** | **Dioctyldiethylenetriamine (OD_600_)** |  |
| 12 | 0.164±0.010 | 0.151±0.008 | ns |
| 14 | 0.299±0.011 | 0.253±0.005 | ** |
| 16 | 0.411±0.009 | 0.374±0.008 | ** |
| 18 | 0.493±0.009 | 0.454±0.005 | ** |
| 20 | 0.547±0.010 | 0.498±0.009 | ** |
| 22 | 0.574±0.010 | 0.539±0.004 | ** |
| 24 | 0.603±0.009 | 0.555±0.007 | ** |
| 36 | 0.655±0.009 | 0.617±0.007 | ** |
| 48 | 0.700±0.009 | 0.677±0.002 | * |
| **Time (h)** | **ΔrpfCG** | | |
|  | **Control (OD_600_)** | **Dioctyldiethylenetriamine (OD_600_)** |  |
| 12 | 0.120±0.007 | 0.031±0.005 | *** |
| 14 | 0.222±0.008 | 0.071±0.004 | *** |
| 16 | 0.314±0.006 | 0.108±0.003 | *** |
| 18 | 0.405±0.004 | 0.137±0.004 | *** |
| 20 | 0.464±0.011 | 0.175±0.004 | *** |
| 22 | 0.501±0.006 | 0.212±0.004 | *** |
| 24 | 0.540±0.005 | 0.244±0.004 | *** |
| 36 | 0.599±0.005 | 0.337±0.003 | *** |
| 48 | 0.650±0.004 | 0.419±0.005 | *** |
| **Time (h)** | **ΔrpfCG(rpfCG)** | | |
|  | **Control (OD_600_)** | **Dioctyldiethylenetriamine (OD_600_)** |  |
| 12 | 0.159±0.003 | 0.151±0.006 | ns |
| 14 | 0.295±0.003 | 0.254±0.006 | *** |
| 16 | 0.405±0.004 | 0.374±0.004 | *** |
| 18 | 0.492±0.004 | 0.456±0.005 | *** |
| 20 | 0.540±0.004 | 0.498±0.004 | *** |
| 22 | 0.571±0.004 | 0.540±0.005 | *** |
| 24 | 0.598±0.004 | 0.556±0.003 | *** |
| 36 | 0.649±0.007 | 0.618±0.003 | ** |
| 48 | 0.692±0.007 | 0.679±0.001 | * |
| **Time (h)** | **ΔrpfBFCG** | | |
|  | **Control (OD_600_)** | **Dioctyldiethylenetriamine (OD_600_)** |  |
| 12 | 0.109±0.011 | 0.017±0.003 | *** |
| 14 | 0.211±0.011 | 0.035±0.003 | *** |
| 16 | 0.304±0.008 | 0.061±0.004 | *** |
| 18 | 0.384±0.009 | 0.082±0.004 | *** |
| 20 | 0.453±0.016 | 0.108±0.004 | *** |
| 22 | 0.487±0.006 | 0.137±0.004 | *** |
| 24 | 0.523±0.006 | 0.166±0.004 | *** |
| 36 | 0.582±0.006 | 0.260±0.009 | *** |
| 48 | 0.622±0.006 | 0.335±0.005 | *** |
| **Time (h)** | **ΔrpfBFCG(rpfBFCG)** | | |
|  | **Control (OD_600_)** | **Dioctyldiethylenetriamine (OD_600_)** |  |
| 12 | 0.153±0.005 | 0.148±0.008 | ns |
| 14 | 0.286±0.009 | 0.251±0.008 | ** |
| 16 | 0.400±0.004 | 0.371±0.005 | ** |
| 18 | 0.486±0.005 | 0.451±0.007 | ** |
| 20 | 0.537±0.006 | 0.493±0.005 | *** |
| 22 | 0.565±0.003 | 0.533±0.005 | *** |
| 24 | 0.593±0.005 | 0.549±0.007 | *** |
| 36 | 0.643±0.006 | 0.617±0.002 | ** |
| 48 | 0.685±0.008 | 0.679±0.001 | ns |

Results for the same strain under the same treatment duration but different dioctyldiethylenetriamine concentrations were analyzed using one-way ANOVA followed by Tukey's multiple range test, with “*” stands for statistically significant differences, *P* < 0.05; “**” stands for statistically significant differences, *P* < 0.01; “***” stands for statistically significant differences, *P* < 0.001; “ns” stands for not statistically significant difference.
