# Supplementary material for: A chemical bactericide dioctyldiethylenetriamine (Xinjunan) exerts a non-lethal effect by inhibiting RpfG activity to regulate the quorum sensing system
Source: PLoS Pathog. 2026 Jun 10;22(6):e1014320. doi: 10.1371/journal.ppat.1014320 (PMC13274925; doi:10.1371/journal.ppat.1014320)
Supplement: S3 Table — (DOCX) [file ppat.1014320.s018.docx]

**S3 Table.** The *in vitro* antibacterial activity of dioctyldiethylenetriamine and dioctyldiethylenetriamine mixtures.

| **Strain** | **Bactericide** | **EC_50(ob)_（μg/mL）** | **EC_50(th)_（μg/mL）** | **Synergy ratio (SR)** |
| --- | --- | --- | --- | --- |
| PXO99A | Dioctyldiethylenetriamine | 0.366 | - | - |
|  | Kasugamycin | 14.128 | - | - |
|  | Zinc thiazole | 3.270 | - | - |
|  | Dioctyldiethylenetriamine : Kasugamycin = 1:9 | 1.673 | 2.968 | 1.774 |
|  | Dioctyldiethylenetriamine : Zinc thiazole = 1:9 | 1.166 | 1.823 | 1.563 |
| ΔrpfG | Dioctyldiethylenetriamine | 0.177 | - | - |
|  | Kasugamycin | 7.227 | - | - |
|  | Zinc thiazole | 2.285 | - | - |
|  | Dioctyldiethylenetriamine : Kasugamycin = 1:9 | 1.331 | 1.450 | 1.089 |
|  | Dioctyldiethylenetriamine : Zinc thiazole = 1:9 | 0.849 | 1.043 | 1.229 |
