# Supplementary material for: A chemical bactericide dioctyldiethylenetriamine (Xinjunan) exerts a non-lethal effect by inhibiting RpfG activity to regulate the quorum sensing system
Source: PLoS Pathog. 2026 Jun 10;22(6):e1014320. doi: 10.1371/journal.ppat.1014320 (PMC13274925; doi:10.1371/journal.ppat.1014320)
Supplement: S4 Table — (DOCX) [file ppat.1014320.s019.docx]

**S4 Table.** Strains used in this study.

| **Strains** | **Characteristics** | **Source** | |
| --- | --- | --- | --- |
| ***Xanthomonas oryzae* pv. *oryzae*** | | | |
| PXO99A | Wild-type | Lab collection | |
| rpfG-GFP-PXO99A | Co-expression of *rpfG* and *GFP* in strain PXO99A. The strain containing a vector of pK18mobSacB::*rpfG-GFP* | This study | |
| GFP-PXO99A | Expression of *GFP* in strain PXO99A. The strain containing a vector of pBBR1MCS5::*GFP* | This study | |
| PXO99A-pBBR | WT strain containing a blank pBBR1MCS5 vector, Gm^R^ | This study | |
| ΔrpfB | PXO_00067 in-frame deletion mutant | This study | |
| ΔrpfB(rpfB) | The ΔrpfB mutant containing a vector of pBBR1MCS5::rpfB | This study | |
| ΔrpfF | PXO_00068 in-frame deletion mutant | This study | |
| ΔrpfF(rpfF) | The ΔrpfF mutant containing a vector of pBBR1MCS5::rpfF | This study | |
| ΔrpfC | PXO_00069 in-frame deletion mutant | This study | |
| ΔrpfC(rpfC) | The ΔrpfC mutant containing a vector of pBBR1MCS5::rpfC | This study | |
| ΔrpfG | PXO_00070 in-frame deletion mutant | This study | |
| ΔrpfG(rpfG) | The ΔrpfG mutant containing a vector of pBBR1MCS5::rpfG | This study | |
| ΔrpfG(*rpfG*^E150A, E194A^) | The ΔrpfG mutant containing a vector of pBBR1MCS5:: *rpfG*^E150A, E194A^ | | This study |
| Δ*rpfG*(*yhjH*) | The Δ*rpfG* mutant containing a vector of pBBR1MCS5::*yhjH* | | This study |
| OE-*rpfG* | PXO_00070 in-frame overexpressed mutant | | This study |
| Δclp | PXO_04006 in-frame deletion mutant | | This study |
| rpfG-GFP-Δclp | Co-expression of *rpfG* and *GFP* in strain Δclp. The strain containing a vector of pK18mobSacB::*rpfG-GFP* | | This study |
| ΔrpfBF | Double mutant in rpfB and rpfF | This study | |
| ΔrpfBF(rpfBF) | The ΔrpfBF mutant containing a vector of pBBR1MCS5::rpfBF | This study | |
| ΔrpfCG | Double mutant in rpfC and rpfG | This study | |
| ΔrpfCG(rpfCG) | The ΔrpfCG mutant containing vectors of pBBR1MCS5::rpfCG | This study | |
| ΔrpfBFCG | Quadruple mutant in rpfB, rpfF, rpfC, and rpfG | This study | |
| ΔrpfBFCG(rpfBFCG) | The ΔrpfBFCG mutant containing vectors of pBBR1MCS5::rpfBFCGG | This study | |
| ΔrpfB-GmR | ΔrpfB strain containing a blank pBBR1MCS5 vector, GmR | This study | |
| ΔrpfF-GmR | ΔrpfF strain containing a blank pBBR1MCS5 vector, GmR | This study | |
| ΔrpfC-GmR | ΔrpfC strain containing a blank pBBR1MCS5 vector, GmR | This study | |
| ΔrpfG-GmR | ΔrpfG strain containing a blank pBBR1MCS5 vector, GmR | This study | |
| ΔrpfBF-GmR | ΔrpfBF strain containing a blank pBBR1MCS5 vector, GmR | This study | |
| ΔrpfCG-GmR | ΔrpfCG strain containing a blank pBBR1MCS5 vector, GmR | This study | |
| ΔrpfBFCG-GmR | ΔrpfBFCG strain containing a blank pBBR1MCS5 vector, GmR | This study | |
| pHM1-*P_rpfG_*-PXO99A | The PXO99A strain containing pHM1::T0T1-*P_rpfG_-gus* | This study | |
| pHM1-*P_rpfG_*-Δclp | The Δclp strain containing pHM1::T0T1-*P_rpfG_-gus* | This study | |
| pHM1-GUS-PXO99A | The PXO99A strain containing pHM1::T0T1-*gus* | This study | |
| pHM1-GUS-Δclp | The Δclp strain containing pHM1::T0T1-*gus* | This study | |
| ***Escherichia coli*** | | | |
| *Trans*1-T1 | F^-^ φ80(*lac*Z)ΔM15Δ*lac*X74*hsd*R(r_k_^-^, m_k_^-^)Δ*rec*A1398*end*A1*ton*A | TransGen Biotech, Beijing, China | |
| BL21(DE3) | F^-^ *omp*T *hsd*S_B_(r_B_^-^ m_B_^-^) *gal dcm* (DE3) | TransGen Biotech, Beijing, China | |
| BL21(DE3)-rpfG | BL21(DE3) strain containing pET28a::*rpfG*, Km^R^ | This study | |
| BL21(DE3)-rpfG^E150A, E194A^ | BL21(DE3) strain containing pET28a:: *rpfG*^E150A, E194A^, Km^R^ | This study | |
| XL1-Blue MRF’ Kan | Δ(*mcrA*)183Δ(mcrCB-hsdSMR-mrr)173, *endA1*, *supE44*, *thi-1*, *recA1* *gyrA96*, *relA1*, *lac*, [F’ *proAB laclqZ*ΔM15 Tn5], Km^R^ | Kindly donated by Guoliang Qian, Nanjing Agricultural University | |
| XL1-Blue (negative control) | Negative control strain of the bacterial one-hybrid system | Kindly donated by Guoliang Qian, Nanjing Agricultural University | |
| XL1-Blue (positive control) | Positive control strain of the bacterial one-hybrid system | Kindly donated by Guoliang Qian, Nanjing Agricultural University | |
| XL1-Blue (PTRG-Clp & pBXcmT) | XL1-Blue MRF’ Kan strain containing PTRG::Clp and pBXcmT | This study | |
| XL1-Blue (PTRG & pBXcmT-*P_rpfG_*) | XL1-Blue MRF’ Kan strain containing PTRG and pBXcmT::*P_rpfG_* | This study | |
| XL1-Blue (PTRG-Clp & pBXcmT-*P_rpfG_*) | XL1-Blue MRF’ Kan strain containing PTRG-Clp and pBXcmT::*P_rpfG_* | This study | |
| *Xanthomonas oxyzae* pv. *oyzicola* | | Lab collection | |
| *Xanthomonas campestris* pv. *campestris* | | Lab collection | |
| *Xanthomonas citri* pv. *citri* | | Lab collection | |
| *Xanthomonas vesicatoria* | | Lab collection | |
| *Stenotrophomonas maltophilia* | | Kindly donated by Yanhong Shi, Anhui Agricultural University | |
| *Agrobacterium tumefaciens* | | Lab collection | |
| *Pectobacterium carotovorum* subsp*. carotovorum* | | Lab collection | |
| *Ralstonia solanacearum* | | Lab collection | |
| *Acidovorax citrulli* | | Lab collection | |
| *Pseudomonas syringae* pv. *syringae* | | Kindly donated by Junhua Zhang, Northeast Agricultural University | |
| *Agrobacterium tumefaciens* JZA1 | | Kindly donated by Zengtao Zhong, Nanjing Agricultural University | |
| *Acidovorax avenae* subsp. *avenae* | | Kindly donated by Bin Li, Zhejiang University | |
| MSZFGNb (*Pseudomonas* spp., endophytic bacteria of rice) | | Kindly donated by Chenyang He, Chinese Academy of Agricultural Sciences | |
