# Supplementary material for: A chemical bactericide dioctyldiethylenetriamine (Xinjunan) exerts a non-lethal effect by inhibiting RpfG activity to regulate the quorum sensing system
Source: PLoS Pathog. 2026 Jun 10;22(6):e1014320. doi: 10.1371/journal.ppat.1014320 (PMC13274925; doi:10.1371/journal.ppat.1014320)
Supplement: S5 Table — (DOCX) [file ppat.1014320.s020.docx]

**S5 Table.** Primers used in this study.

| **Primer name** | **Sequence (5’-3’)** | **Description** |
| --- | --- | --- |
| rpfG-up-GFP-pK18(F) | TACCCGGGGATCCTCTAGAGATCTGTTGCTGCTGGACTACC | Used for construction of pK18mobSacB::*rpfG-GFP* vector. |
| rpfG-up-GFP-pK18(R) | GAAAAGTTCTTCTCCTTTACTCATCACCCCGGGTCGCGCCGATG |  |
| GFP-rpfG-up(F) | CATCGGCGCGACCCGGGGTGATGAGTAAAGGAGAAGAACTTTTC |  |
| GFP-rpfG-down(R) | CCAGGCGCCGCCCTCACCCTATTTGTATAGTTCATCCATGCC |  |
| rpfG-down-GFP-pK18(F) | GGCATGGATGAACTATACAAATAGGGTGAGGGCGGCGCCTGG |  |
| rpfG-down-GFP-pK18(R) | CTTGCATGCCTGCAGGTCGAGCCAGCGATACCACCAGTTC |  |
| diy-pBBR1MCS5(F) | GCGGTGGCGGCCGCTCTAGAACCGTAGCCCTTGCCTACCTAG | Used for construction the constitutive expression vector of *GFP*. |
| diy(R) | AATGTGACTTTCCTCCTTAAGCTT |  |
| GFP-diy(F) | CTTAAGGAGGAAAGTCACATTATGAGTAAAGGAGAAGAACTTTTC |  |
| GFP-pBBR1MCS5(R) | GTCGACGGTATCGATAAGCTTGCTATTTGTATAGTTCATCCATGCC |  |
| rpfG-pET28a(F) | AGCAAATGGGTCGCGGATCCATGGGATTGAACATCGTCATTGTC | Used for construction of pET28a::*rpfG* vector. |
| rpfG-pET28a(R) | CGAGTGCGGCCGCAAGCTTGTCACACCCCGGGTCGCGCC |  |
| RpfG^E150A, E194A^-pET28a(up-F) | AGCAAATGGGTCGCGGATCCATGGGATTGAACATCGTCATTGTC | Used for construction of pET28a::*rpfG*^E150A, E194A^ vector. |
| RpfG^E150A, E194A^-pET28a(up-R) | GCGTTCTGCGACCTCGTTC |  |
| RpfG^E150A, E194A^-pET28a(middle-F) | GAACGAGGTCGCAGAACGC |  |
| RpfG^E150A, E194A^-pET28a(middle -R) | CGCACCTCTGCTTCCGAC |  |
| RpfG^E150A, E194A^-pET28a(down-F) | GTCGGAAGCAGAGGTGCG |  |
| RpfG^E150A, E194A^-pET28a(down-R) | CGAGTGCGGCCGCAAGCTTGTCACACCCCGGGTCGCGCC |  |
| rpfB(up-F) | TACCCGGGGATCCTCTAGAGGCCTCCCCGCCGTTGGTG | Used for construction of in-frame deletion mutant of *rpfB* by pK18mobSacB. |
| rpfB(up-R) | ATAACCAGTACTGCCCCCCTCCAGGGTG |  |
| rpfB(down-F) | AGGGGGGCAGTACTGGTTATACCGAAGACCAAAGA |  |
| rpfB(down-R) | CTTGCATGCCTGCAGGTCGAGCACACAGCGTTGGGCGTA |  |
| rpfF(up-F) | TACCCGGGGATCCTCTAGAGAGGCTTCGTCTACATCGTCG | Used for construction of in-frame deletion mutant of *rpfF* by pK18mobSacB. |
| rpfF(up-R) | CCGCCGCACAGTTGGTTCTCCGTGATGACCG |  |
| rpfF(down-F) | GAGAACCAACTGTGCGGCGGGCTATTCG |  |
| rpfF(down-R) | CTTGCATGCCTGCAGGTCGATGCGTTTCTGGCCAAGCC |  |
| rpfC(up-F) | TACCCGGGGATCCTCTAGAGACCTGTATGCCCAACGCG | Used for construction of in-frame deletion mutant of *rpfC* by pK18mobSacB. |
| rpfC(up-R) | GTGCGGCGGGCCATGTCAGTGGAGACTTCATAGAC |  |
| rpfC(down-F) | ACTGACATGGCCCGCCGCACATCAGCCG |  |
| rpfC(down-R) | CTTGCATGCCTGCAGGTCGATGCGCACAGCATTGCCCTGG |  |
| rpfG(up-F) | TACCCGGGGATCCTCTAGAGGGCTATACCGACCGCTTC | Used for construction of in-frame deletion mutant of *rpfG* by pK18mobSacB. |
| rpfG(up-R) | CGCCCTCACCATCCGCCTTTTCCGCACAG |  |
| rpfG(down-F) | AAAGGCGGATGGTGAGGGCGGCGCCTGG |  |
| rpfG(down-R) | CTTGCATGCCTGCAGGTCGAAAGGTGTCGCCGTGGGTGTTGTGG |  |
| clp(up-F) | TACCCGGGGATCCTCTAGAGCAGTGATATGACTCTTGTCCATGTG | Used for construction of in-frame deletion mutant of *rpfG* by pK18mobSacB. |
| clp(up-R) | TGGTGCCGTGAACCGTCGTGTTTGCTGAGC |  |
| clp(down-F) | CACGACGGTTCACGGCACCACGCAGCCAGTGC |  |
| clp(down-R) | CTTGCATGCCTGCAGGTCGAGCGCGGCTATGCCGATATCG |  |
| rpfBF(up-F) | TACCCGGGGATCCTCTAGAGGTTGTCTTACGCCAACCGG | Used for construction of in-frame deletion mutant of *rpfB* and *rpfF* by pK18mobSacB. |
| rpfBF(up-R) | CGAATAGCCCGCCGCACACTGCCCCCCTCCAGGGT |  |
| rpfBF(down-F) | ACCCTGGAGGGGGGCAGTGTGCGGCGGGCTATTCG |  |
| rpfBF(down-R) | CTTGCATGCCTGCAGGTCGAGTGCGTTTCTGGCCAAGCC |  |
| rpfCG(up-F) | TACCCGGGGATCCTCTAGAGGCGCACAGCATTGCCCTG | Used for construction of in-frame deletion mutant of *rpfC* and *rpfG* by pK18mobSacB. |
| rpfCG(up-R) | CTGTGCGGAAAAGGCGGATCCCGCCGCACATCAGCC |  |
| rpfCG(down-F) | GGCTGATGTGCGGCGGGATCCGCCTTTTCCGCACAG |  |
| rpfCG(down-R) | CTTGCATGCCTGCAGGTCGAGCTATACCGACCGCTTCGA |  |
| rpfBFCG(up-F) | TACCCGGGGATCCTCTAGAGGTTGTCTTACGCCAACCGG | Used for construction of in-frame deletion mutant of *rpfB*, *rpfF*, *rpfC*, and *rpfG* by pK18mobSacB. |
| rpfBFCG(up-R) | CTGTGCGGAAAAGGCGGATCTGCCCCCCTCCAGGGT |  |
| rpfBFCG(down-F) | ACCCTGGAGGGGGGCAGATCCGCCTTTTCCGCACAG |  |
| rpfBFCG(down-R) | CTTGCATGCCTGCAGGTCGAGCTATACCGACCGCTTCGA |  |
| rpfB(comp-F) | GCGGTGGCGGCCGCTCTAGAACGTTGTCTTACGCCAACCGG | Used for construction genetic complementary vector of *rpfB*. |
| rpfB(comp-R) | GTCGACGGTATCGATAAGCTTGTTACGTTTTCGGCGCGTCC |  |
| rpfF(comp-F) | GCGGTGGCGGCCGCTCTAGAACTTCGTCTACATCGTCGATCGC | Used for construction genetic complementary vector of *rpfF*. |
| rpfF(comp-R) | GTCGACGGTATCGATAAGCTTGTCAGCCGGCGTCAAGCC |  |
| rpfC(comp-F) | GCGGTGGCGGCCGCTCTAGAACTCGACCCGCGCTGCGTG | Used for construction genetic complementary vector of *rpfC*. |
| rpfC(comp-R) | GTCGACGGTATCGATAAGCTTGCTATTCGCTGCTCCGGGG |  |
| rpfG(comp-F) | GCGGTGGCGGCCGCTCTAGAACCATCAATGGCAAGGAGCTGG | Used for construction genetic complementary vector of *rpfG*. |
| rpfG(comp-R) | GTCGACGGTATCGATAAGCTTGTCACACCCCGGGTCGCG |  |
| rpfBF(comp-F) | GCGGTGGCGGCCGCTCTAGAACGTTGTCTTACGCCAACCGG | Used for construction genetic complementary vector of *rpfB* and *rpfF*. |
| rpfBF(comp-R) | GTCGACGGTATCGATAAGCTTGTCAGCCGGCGTCAAGCC |  |
| rpfCG(comp-F) | GCGGTGGCGGCCGCTCTAGAACGCGCACAGCATTGCCCTG | Used for construction genetic complementary vector of *rpfC* and *rpfG*. |
| rpfCG(comp-R) | GTCGACGGTATCGATAAGCTTGGCTATACCGACCGCTTCGA |  |
| rpfBFCG(comp-F) | GCGGTGGCGGCCGCTCTAGAACGTTGTCTTACGCCAACCGG | Used for construction genetic complementary vector of *rpfB*, *rpfF*, *rpfC*, and *rpfG*. |
| rpfBFCG(comp-R) | GTCGACGGTATCGATAAGCTTGGCTATACCGACCGCTTCGA |  |
| RpfG^E150A, E194A^(up-F) | GCGGTGGCGGCCGCTCTAGAACCATCAATGGCAAGGAGCTGG | Used for construction the expression vector of *rpfG*^E150A, E194A^. |
| RpfG^E150A, E194A^(up-R) | GCGTTCTGCGACCTCGTTC |  |
| RpfG^E150A, E194A^(middle-F) | GAACGAGGTCGCAGAACGC |  |
| RpfG^E150A, E194A^(middle -R) | CGCACCTCTGCTTCCGAC |  |
| RpfG^E150A, E194A^(down-F) | GTCGGAAGCAGAGGTGCG |  |
| RpfG^E150A, E194A^(down-R) | GTCGACGGTATCGATAAGCTTGTCACACCCCGGGTCGCG |  |
| diy-pBBR1MCS5(F) | GCGGTGGCGGCCGCTCTAGAACCGTAGCCCTTGCCTACCTAG | Used for construction the constitutive expression vector of *yhjH*. |
| diy(R) | AATGTGACTTTCCTCCTTAAGCTT |  |
| yhjH-diy(F) | CTTAAGGAGGAAAGTCACATTATGATAAGGCAGGTTATCCAGC |  |
| yhjH-pBBR1MCS5(R) | GTCGACGGTATCGATAAGCTTGTTATAGCGCCAGAACCGCC |  |
| diy-pBBR1MCS5(F) | GCGGTGGCGGCCGCTCTAGAACCGTAGCCCTTGCCTACCTAG | Used for construction the overexpression expression vector of *rpfG*. |
| diy(R) | AATGTGACTTTCCTCCTTAAGCTT |  |
| rpfG-diy(F) | AAGCTTAAGGAGGAAAGTCACATTATGGGATTGAACATCGTCATTGTC |  |
| rpfG-pBBR1MCS5(R) | GTCGACGGTATCGATAAGCTTGTCACACCCCGGGTCGCGCC |  |
| PTRG-Clp (F) | CCGCGGCCGCAAGAATTCAGGTGACTACGACGGTACGTAAC | Used for construction of PTRG::Clp vector. |
| PTRG-Clp (R) | TAATTAATTAATTACTCGAGTTAGCGCGTGCCGTACAGC |  |
| pBXcmT-P_rpfG_ (F) | AAAGTGGGGGATCCGAATTCATCGACCGGCTGGTGATGC | Used for construction of pBXcmT::*P_rpfG_* vector. |
| pBXcmT-P_rpfG_ (R) | TTCGACAAGGATCCTCTAGAATCCGCCTTTTCCGCACAG |  |
| T0T1-pHM1 (F) | GCAGCGGAGGGGTTGGATCCGACTCCTGTTGATAGATCCAGTAA | Used for construction of pHM1::T0T1 |
| T0T1-pHM1 (R) | TTCAAATCCGCTCCCGGCGGATTTGTCCTACTCAGGAGAGCG |  |
| P_rpfG_-pHM1::T0T1 (F) | TTGCATGCCTGCAGGTCGACGGCTATACCGACCGCTTCG | Used for construction of pHM1::T0T1-*P_rpfG_-gus* vector |
| P_rpfG_-GUS (R) | GTTTCTACAGGACGTAACATATCCGCCTTTTCCGCACAGC |  |
| GUS (F) | ATGTTACGTCCTGTAGAAACC |  |
| GUS-pHM1::T0T1 (R) | CCCAAAAGCTTTCAGAATTCTCATTGTTTGCCTCCCTGCT |  |
| GUS-pHM1::T0T1 (F) | TTGCATGCCTGCAGGTCGACATGTTACGTCCTGTAGAAACC | Used for construction of pHM1::T0T1-*gus* vector |
| GUS-pHM1::T0T1 (R) | CCCAAAAGCTTTCAGAATTCTCATTGTTTGCCTCCCTGCT |  |
| PXO_00067(qF) | TTCGCTGCTTACCTGCTTGG | Used for qRT-PCR. |
| PXO_00067(qR) | GTCGATCAACTGATGCTTGAGTT |  |
| PXO_00068(qF) | TCACCCGCTATCAGACCAAC |  |
| PXO_00068(qR) | AATGAGTCGGCAAAACAAGGC |  |
| PXO_00069(qF) | TATTTGAGTTGGGGCTTGCT |  |
| PXO_00069(qR) | CATTCCTTCTGCTCTACATC |  |
| PXO_00070(qF) | CTGGGGTTGTCGGAAGAAGA |  |
| PXO_00070(qR) | ACGCTCATCTCGTCGTCAGT |  |
| PXO_03417(qF) | CACGCAGAACTACATCGCAT |  |
| PXO_03417(qR) | GGGCAGATAGTCGGAATAGC |  |
| PXO_01578(qF) | CTCAAGTTGATCGTCACCTC |  |
| PXO_01578(qR) | GCCTTCCACATTGATCACC |  |
| PXO_03410(qF) | CCGATGCGGTCACTCAGG |  |
| PXO_03410(qR) | GATGCCGTCATGCAGGAC |  |
| PXO_01008(qF) | ACCCGCCGACCGTTTGAC |  |
| PXO_01008(qR) | CCACGTTGCTGCCTTCCA |  |
| PXO_01004(qF) | TCTCAACCCGAGCACCAAG |  |
| PXO_01004(qR) | GAAATCATCGCCGACAAGC |  |
| PXO_01003(qF) | GTCCTCGCTGTCCAACCG |  |
| PXO_01003(qR) | CGATTGACCTCATCTACCG |  |
| PXO_04882(qF) | AATCGGTTGTTTGTGGGAGT |  |
| PXO_04882(qR) | GACCAGATTCGTATGAAACTTG |  |
| PXO_04886(qF) | CGGCTTATCAGGACTACACCAC |  |
| PXO_04886(qR) | GCGAGAGTAACATTAGCAACAC |  |
| PXO_01993(qF) | CAAGAACAAGGCATCGGACC |  |
| PXO_01993(qR) | TTCTGGTTGAACGCATTGAC |  |
| PXO_00481(qF) | GGCAAGTCCTCCATCAACG |  |
| PXO_00481(qR) | CAGCAGGTAGGCATTGTTG |  |
| PXO_00482(qF) | CCTCGGGTGCGATCTACAAG |  |
| PXO_00482(qR) | GTTCTTCTTGGTGGAGTGGG |  |
| PXO_01155(qF) | CAAGACCCGCTTCTATCCC |  |
| PXO_01155(qR) | CCGAACACCGAGCCACTG |  |
| PXO_00256(qF) | AGGTGGTGGTGAGCTTTCTG |  |
| PXO_00256(qR) | GACAGGCTGCGGCTCTTTAC |  |
| PXO_00264(qF) | CCATACCAAGCACAAGGACCA |  |
| PXO_00264(qR) | CAGGCGTTGAGCAAGGCAT |  |
| PXO_02043(qF) | ATATCCGCCCACCGTTTCAA |  |
| PXO_02043(qR) | AGACGCCTCTGGCTTTGCTT |  |
| PXO_04699(qF) | CGCATCCAGGAAGCCAGAC |  |
| PXO_04699(qR) | CAGGCGTAGGTCCTTGGTA |  |
| PXO_02051(qF) | CGGCGTCTTCCTCTTCGTC |  |
| PXO_02051(qR) | TCCTGGTACTCGCTCATGT |  |
| PXO99A-gyrB(qF) | ACGGCACTTACGACTCCAG |  |
| PXO99A-gyrB(qR) | CGACCACCTCGAACACCAT |  |
| Xoc-rpfB(qF) | ATGCTGACTCACCGCAATCTG |  |
| Xoc-rpfB(qR) | CGGGTCTTTTTCAGCTCCTT |  |
| Xoc-rpfF(qF) | TGAAGAACCGCAGCGTGAG |  |
| Xoc-rpfF(qR) | ATGAGTCGGCAAAACAAGGC |  |
| Xoc-rpfC(qF) | CTATTTGAGTTGGGGCTTGCT |  |
| Xoc-rpfC(qR) | GCATTCCTTCTGCTCTACATCC |  |
| Xoc-rpfG(qF) | CAGCAGAGCGAAAGCGTCA |  |
| Xoc-rpfG(qR) | TCTTCTTCCGACAACCCCA |  |
| Xoc-gyrB(qF) | CCGACGAACAAAACACCCC |  |
| Xoc-gyrB(qR) | CGACCACCTCGAACACCAT |  |
| Xcc-rpfB(qF) | CTGGAGCAGTTCAGCACCGT |  |
| Xcc-rpfB(qR) | CAGGCAGTTGGGCATCATC |  |
| Xcc-rpfF(qF) | GCAGTTCAACCCTTCATTCGT |  |
| Xcc-rpfF(qR) | GGTAGCCAGTGATGTCGTCG |  |
| Xcc-rpfC(qF) | CTATCTGAGTTGGGGGTTGTTG |  |
| Xcc-rpfC(qR) | GGTCGCCAGCACTTCGGT |  |
| Xcc-rpfG(qF) | AGAGCGAGAGCGTCAAGCAA |  |
| Xcc-rpfG(qR) | TTCTTCTTCCGACAAGCCCA |  |
| Xcc-gyrB(qF) | GGCACCTACGACTCCAGCAA |  |
| Xcc-gyrB(qR) | TCGACCACCTCGAACACCAT |  |
| Xac-rpfB(qF) | GCTTACCTGCTTGGCGAGT |  |
| Xac-rpfB(qR) | GGGGTGTACAGCGGATTGA |  |
| Xac-rpfF(qF) | TACGCCACTTCATCCTCGC |  |
| Xac-rpfF(qR) | GCCTTCCACGCAACGCT |  |
| Xac-rpfC(qF) | GCCTTGGGCTGGGACTGT |  |
| Xac-rpfC(qR) | CAGGGTCGTGGCGAAACT |  |
| Xac-rpfG(qF) | TCTGCTGTTGCTGGACTACC |  |
| Xac-rpfG(qR) | GACGCTTTCGCTCTGCTGAC |  |
| Xac-gyrB(qF) | ACCGACGCCTACCAGGAAAC |  |
| Xac-gyrB(qR) | CCTTGGTCTGCGAAGAGAAG |  |
| Xve-rpfB(qF) | GAACGCTGGAAAAAGGTCAC |  |
| Xve-rpfB(qR) | AGGCATCGGTGGACGGAA |  |
| Xve-rpfF(qF) | CCAAGCCAACCTGGGACA |  |
| Xve-rpfF(qR) | TCGCCACCGAGATTGAAGAC |  |
| Xve-rpfC(qF) | CACACCCACGGCGATACC |  |
| Xve-rpfC(qR) | CATCAACCCAAGCGAGACC |  |
| Xve-rpfG(qF) | CCTGTCGGAAGAGGAGGTGC |  |
| Xve-rpfG(qR) | TGGATGAAACGGTTCTGGCT |  |
| Xve-gyrB(qF) | CAATGGACCGACGCCTACC |  |
| Xve-gyrB(qR) | CTTCACCGACAGCACCGC |  |
| Sm-rpfB(qF) | AACGGCAACATCCTGGCG |  |
| Sm-rpfB(qR) | GCGGTGGCGGTTTCCTC |  |
| Sm-rpfF(qF) | GCCTACTCCTTCCTGTGCCA |  |
| Sm-rpfF(qR) | CAACACCTTCGCCCACCG |  |
| Sm-rpfC(qF) | GTCCCTCGCAGCACCCTAC |  |
| Sm-rpfC(qR) | CGTCGCTGTCAGAGGCAAGT |  |
| Sm-rpfG(qF) | TCATTGTTGACGACCAGACG |  |
| Sm-rpfG(qR) | CTCCAGCCCATCCATCTCC |  |
| Sm-gyrB(qF) | ACGACGCCAACAGCATTACC |  |
| Sm-gyrB(qR) | TTGCCAGTCGGGATACCA |  |
| At-rpfB(qF) | CGCATCCGCCAATACCTCC |  |
| At-rpfB(qR) | GGCATCATCACGGCAACCC |  |
| At-rpfG(qF) | CCTGACGCCTGATGAGATTAC |  |
| At-rpfG(qR) | TTCCATCCCATTTTTCGTGA |  |
| At-TraI(qF) | TGGAGCAGACTTTTTCGCAA |  |
| At-TraI(qR) | TAAGGTAAGAGTCGCCAGGTG |  |
| At-TraR(qF) | CCTTCAGACCACTCCGACAG |  |
| At-TraR(qR) | GAGGGCGGTGAGATGGG |  |
| At-gyrB(qF) | CTCACCGACAAACGCCACTC |  |
| At-gyrB(qR) | TACCGTCCTTCTCGCTGTGA |  |
| Pcc-expI(qF) | TTCTCGGCAATGAATACCCA |  |
| Pcc-expI(qR) | AGCCAACGCTTCCTGATTCT |  |
| Pcc-expR(qF) | TCCCGTTGTTCTTGCTTCAT |  |
| Pcc-expR(qR) | ATAGCATAGCCAGATTGTCGC |  |
| Pcc-gyrB(qF) | CCGCTGAGTCCCCTTCCA |  |
| Pcc-gyrB(qR) | GGATGCCAAAATCGTAGTCG |  |
| Rs-rasI(qF) | ATCTTGGTCGCTATCGCTACA |  |
| Rs-rasI(qR) | GACGCTTTCCAGCAAATAGGG |  |
| Rs-rasR(qF) | TATGGGATGAGGCGAAGGC |  |
| Rs-rasR(qR) | TGGTCAGGTTATCGTCGGAAT |  |
| Rs-gyrB(qF) | GACCGAACAGCAGAAACCG |  |
| Rs-gyrB(qR) | GTCCAGCACCTCGAACACG |  |
| Ac-aacI(qF) | CCACGACGCAGCCCTACAT |  |
| Ac-aacI(qR) | AGTTCCGCCACGGTCTGTC |  |
| Ac-aacR(qF) | GCTTTGAACAGGCGGTGCT |  |
| Ac-aacR(qR) | GTCTTTCCAGCGATAGGGC |  |
| Ac-actin(qF) | CCATGTATGTTGCCATCCAG |  |
| Ac-actin(qR) | GGATAGCATGGGGTAGAGCA |  |
| Pss-psyI(qF) | GAAACGCTGTATGCCCTGC |  |
| Pss-psyI(qR) | ACTGCGAAACGGTCCATCC |  |
| Pss-psyR(qF) | AACTATCCTGATTCCTGGCTGA |  |
| Pss-psyR(qR) | CAAAGACTCAGAGCGGCAAA |  |
| Pss-gyrB(qF) | TCCACAGGAACCGATGAAGAT |  |
| Pss-gyrB(qR) | CTTGAGGACAATGCCTACACC |  |
