# Supplementary material for: A chemical bactericide dioctyldiethylenetriamine (Xinjunan) exerts a non-lethal effect by inhibiting RpfG activity to regulate the quorum sensing system
Source: PLoS Pathog. 2026 Jun 10;22(6):e1014320. doi: 10.1371/journal.ppat.1014320 (PMC13274925; doi:10.1371/journal.ppat.1014320)
Supplement: S6 Table — (DOCX) [file ppat.1014320.s021.docx]

**S6 Table.** Plasmids used in this study.

| **Plasmid** | **Characteristics** | **Source** |
| --- | --- | --- |
| pK18mobSacB | Suicide vector for gene in-frame deletion, Km^R^ | Lab collection |
| pBBR1MCS5 | Broad-host-vector for gene complementation, Gm^R^ | Lab collection |
| pET28a | Vector for expression His-tag fusion protein | Lab collection |
| PTRG | The plasmid used for protein expression in bacterial one-hybrid assay, Tet^R^ | Kindly donated by Guoliang Qian, Nanjing Agricultural University |
| pBXcmT | The plasmid used for DNA cloning in bacterial one-hybrid assay, Chl^R^ | Kindly donated by Guoliang Qian, Nanjing Agricultural University |
| pHM1 | Broad host vector, S^R^ | Lab collection |
| pK18mobSacB-*rpfG-GFP* | pK18mobSacB::*rpfG-GFP*, recombinant suicide vector for constructing co-expressed by *rpfG* and *GFP*, Km^R^ | This study |
| pBBR1MCS5-*GFP* | pBBR1MCS5::*GFP*, recombinant vector for constitutively expressing *GFP*, Gm^R^ | This study |
| pET28a-*rpfG* | pET28a::*rpfG*, expressing full-length *rpfG*, Km^R^ | This study |
| pET28a-*rpfG*^E150A, E194A^ | pET28a::*rpfG*^E150A, E194A^, expressing full-length *rpfG*^E150A, E194A^, Km^R^ | This study |
| pK18mobSacB-*rpfB* | pK18mobSacB::*rpfB*, recombinant suicide vector for constructing *rpfB* in-frame deletion mutant, Km^R^ | This study |
| pK18mobSacB-*rpfF* | pK18mobSacB::*rpfF*, recombinant suicide vector for constructing *rpfF* in-frame deletion mutant, Km^R^ | This study |
| pK18mobSacB-*rpfC* | pK18mobSacB::*rpfC*, recombinant suicide vector for constructing *rpfC* in-frame deletion mutant, Km^R^ | This study |
| pK18mobSacB-*rpfG* | pK18mobSacB::*rpfG*, recombinant suicide vector for constructing *rpfG* in-frame deletion mutant, Km^R^ | This study |
| pK18mobSacB-*clp* | pK18mobSacB:: *clp*, recombinant suicide vector for constructing *clp* in-frame deletion mutant, Km^R^ | This study |
| pK18mobSacB-*rpfBF* | pK18mobSacB::*rpfBF*, recombinant suicide vector for constructing *rpfBF* in-frame deletion mutant, Km^R^ | This study |
| pK18mobSacB-*rpfCG* | pK18mobSacB::*rpfCG*, recombinant suicide vector for constructing *rpfCG* in-frame deletion mutant, Km^R^ | This study |
| pK18mobSacB-*rpfBFCG* | pK18mobSacB::*rpfBFCG*, recombinant suicide vector for constructing *rpfBFCG* in-frame deletion mutant, Km^R^ | This study |
| pBBR1MCS5-*rpfB* | pBBR1MCS5::*rpfB*, recombinant vector for genetic complementation of *rpfB* mutant, Gm^R^ | This study |
| pBBR1MCS5-*rpfF* | pBBR1MCS5::*rpfF*, recombinant vector for genetic complementation of *rpfF* mutant, Gm^R^ | This study |
| pBBR1MCS5-*rpfC* | pBBR1MCS5::*rpfB*, recombinant vector for genetic complementation of *rpfC* mutant, Gm^R^ | This study |
| pBBR1MCS5-*rpfG* | pBBR1MCS5::*rpfG*, recombinant vector for genetic complementation of *rpfG* mutant, Gm^R^ | This study |
| pBBR1MCS5-diy-*rpfG* | pBBR1MCS5::diy-*rpfG*, recombinant vector for overexpression of *rpfG*, Gm^R^ | This study |
| pBBR1MCS5-*rpfG*^E150A, E194A^ | pBBR1MCS5::*rpfG*^E150A, E194A^, recombinant vector for constitutively expressing *rpfG*^E150A, E194A^, Gm^R^ | This study |
| pBBR1MCS5-*yhjH* | pBBR1MCS5::*yhjH*, recombinant vector for constitutively expressing *yhjH*, Gm^R^ | This study |
| pBBR1MCS5-*rpfBF* | pBBR1MCS5::*rpfBF*, recombinant vector for genetic complementation of *rpfBF* mutant, Gm^R^ | This study |
| pBBR1MCS5-*rpfCG* | pBBR1MCS5::*rpfCG*, recombinant vector for genetic complementation of *rpfCG* mutant, Gm^R^ | This study |
| pBBR1MCS5-*rpfBFCG* | pBBR1MCS5::*rpfBFCG*, recombinant vector for genetic complementation of *rpfBFCG* mutant, Gm^R^ | This study |
| PTRG-Clp | PTRG::Clp, pTRG with the coding region of Clp, Tet^R^ | This study |
| pBXcmT-*P_rpfG_* | pBXcmT::*P_rpfG_*, pBXcmT with the *rpfG* promoter region, Chl^R^ | This study |
| pHM1-T0T1-*P_rpfG_-gus* | pHM1::T0T1-*P_rpfG_-gus*, pHM1 with the termination sequence T0T1, the *rpfG* promoter region and the *gus*, S^R^ | This study |
| pHM1-T0T1-*gus* | pHM1::T0T1-*gus*, pHM1 with the termination sequence T0T1 and the *gus*, S^R^ | This study |
