# Supplementary material for: A chemical bactericide dioctyldiethylenetriamine (Xinjunan) exerts a non-lethal effect by inhibiting RpfG activity to regulate the quorum sensing system
Source: PLoS Pathog. 2026 Jun 10;22(6):e1014320. doi: 10.1371/journal.ppat.1014320 (PMC13274925; doi:10.1371/journal.ppat.1014320)
Supplement: S7 Table — (DOCX) [file ppat.1014320.s022.docx]

**S7 Table.** Kyoto Encyclopedia of Genes and Genomes (KEGG) pathway enrichment analysis of differential genes.

| **Pathway name** | **Pathway id** | **Input number** | **Background number** |
| --- | --- | --- | --- |
| Quorum sensing | ko02024 | 9 | 34 |
| Degradation of aromatic compounds | ko01220 | 3 | 9 |
| Cysteine and methionine metabolism | ko00270 | 8 | 41 |
| Glycine, serine and threonine metabolism | ko00260 | 6 | 29 |
| Polyketide sugar unit biosynthesis | ko00523 | 2 | 5 |
| Benzoate degradation | ko00362 | 4 | 18 |
| Folate biosynthesis | ko00790 | 5 | 27 |
| Glutathione metabolism | ko00480 | 3 | 18 |
| Purine metabolism | ko00230 | 6 | 43 |
| Glycolysis/Gluconeogenesis | ko00010 | 3 | 20 |
| ABC transporters | ko02010 | 7 | 55 |
| Fatty acid degradation | ko00071 | 2 | 12 |
| Protein export | ko03060 | 2 | 13 |
| Nicotinate and nicotinamide metabolism | ko00760 | 2 | 13 |
| Amino sugar and nucleotide sugar metabolism | ko00520 | 3 | 24 |
| Pantothenate and CoA biosynthesis | ko00770 | 2 | 15 |
| DNA replication | ko03030 | 2 | 16 |
| RNA degradation | ko03018 | 2 | 16 |
| Vitamin B6 metabolism | ko00750 | 1 | 7 |
| Mismatch repair | ko03430 | 2 | 17 |
| 2-Oxocarboxylic acid metabolism | ko01210 | 2 | 18 |
| Biosynthesis of antibiotics | ko01130 | 20 | 176 |
| C5-Branched dibasic acid metabolism | ko00660 | 1 | 8 |
| Biosynthesis of amino acids | ko01230 | 11 | 94 |
| Lysine degradation | ko00310 | 1 | 8 |
| Oxidative phosphorylation | ko00190 | 5 | 49 |
| Citrate cycle (TCA cycle) | ko00020 | 2 | 19 |
| Lipopolysaccharide biosynthesis | ko00540 | 2 | 19 |
| Pentose phosphate pathway | ko00030 | 2 | 20 |
| Butanoate metabolism | ko00650 | 2 | 20 |
| Propanoate metabolism | ko00640 | 2 | 21 |
| Phenylalanine, tyrosine, and tryptophan biosynthesis | ko00400 | 2 | 21 |
| Selenocompound metabolism | ko00450 | 1 | 10 |
| Homologous recombination | ko03440 | 2 | 22 |
| Glyoxylate and dicarboxylate metabolism | ko00630 | 3 | 33 |
| Carbon metabolism | ko01200 | 7 | 78 |
| Valine, leucine, and isoleucine degradation | ko00280 | 2 | 24 |
| Biotin metabolism | ko00780 | 1 | 12 |
| Drug metabolism - other enzymes | ko00983 | 1 | 12 |
| Ubiquinone and other terpenoid-quinone Biosynthesis | ko00130 | 1 | 12 |
| Terpenoid backbone biosynthesis | ko00900 | 1 | 12 |
| Porphyrin and chlorophyll metabolism | ko00860 | 2 | 25 |
| Valine, leucine, and isoleucine biosynthesis | ko00290 | 1 | 13 |
| Microbial metabolism in diverse environments | ko01120 | 13 | 144 |
| Base excision repair | ko03410 | 1 | 14 |
| Fatty acid metabolism | ko01212 | 2 | 27 |
| Tryptophan metabolism | ko00380 | 1 | 15 |
| Fatty acid biosynthesis | ko00061 | 1 | 16 |
| Fructose and mannose metabolism | ko00051 | 1 | 17 |
| Biosynthesis of secondary metabolites | ko01110 | 24 | 272 |
| Metabolic pathways | ko01100 | 58 | 623 |
| Flagellar assembly | ko02040 | 4 | 66 |
| Aminoacyl - tRNA biosynthesis | ko00970 | 1 | 24 |
| Pyruvate metabolism | ko00620 | 1 | 25 |
| Pyrimidine metabolism | ko00240 | 1 | 25 |
| Bacterial chemotaxis | ko02030 | 1 | 40 |
| Bacterial secretion system | ko03070 | 2 | 60 |
| Two - component system | ko02020 | 6 | 126 |
| Ribosome | ko03010 | 1 | 54 |
