# Supplementary material for: A chemical bactericide dioctyldiethylenetriamine (Xinjunan) exerts a non-lethal effect by inhibiting RpfG activity to regulate the quorum sensing system
Source: PLoS Pathog. 2026 Jun 10;22(6):e1014320. doi: 10.1371/journal.ppat.1014320 (PMC13274925; doi:10.1371/journal.ppat.1014320)
Supplement: S8 Table — (DOCX) [file ppat.1014320.s023.docx]

**S8 Table.** Distribution of gene encoding protein of the RpfG (KO term: K13815) within the KEGG database.

| **Bacteria** | **Number** |
| --- | --- |
| *Xanthomonas* | 55 |
| *Stenotrophomonas* | 21 |
| *Methylobacterium* | 20 |
| *Bradyrhizobium* | 18 |
| *Azospirillum* | 15 |
| *Lysobacter* | 12 |
| *Pseudoxanthomonas* | 9 |
| *Xylella* | 8 |
| *Rhodoferax* | 7 |
| *Herbaspirillum* | 7 |
| *Rhizobium* | 7 |
| *Methylorubrum* | 7 |
| *Agrobacterium* | 6 |
| *Devosia* | 6 |
| *Luteimonas* | 5 |
| *Methylophaga* | 5 |
| *Dechloromonas* | 5 |
| *Azoarcus* | 5 |
| *Arcobacter* | 5 |
| *Thermomonas* | 4 |
| *Undibacterium* | 4 |
| *Magnetospirillum* | 4 |
| *Leptospirillum* | 4 |
| *Janthinobacterium* | 3 |
| *Massilia* | 3 |
| *Azospira* | 3 |
| *Thauera* | 3 |
| *Mesorhizobium* | 3 |
| *Rhodopseudomonas* | 3 |
| *Skermanella* | 3 |
| *Stieleria* | 3 |
| *Marilutibacter* | 2 |
| *Agrilutibacter* | 2 |
| *Thiohalobacter* | 2 |
| *Aquitalea* | 2 |
| *Thiobacillus* | 2 |
| *Methylotenera* | 2 |
| *Aromatoleum* | 2 |
| *Pelagibacterium* | 2 |
| *Labrenzia* | 2 |
| *Phreatobacter* | 2 |
| *Acidithiobacillus* | 2 |
| *Aliarcobacter* | 2 |
| *Halarcobacter* | 2 |
| *Noviluteimonas* | 1 |
| *Novilysobacter* | 1 |
| *Pseudolysobacter* | 1 |
| *Dokdonella* | 1 |
| *Methylospira* | 1 |
| *Methylogaea* | 1 |
| *Thiomicrorhabdus* | 1 |
| *Thiothrix* | 1 |
| *Thioflavicoccus* | 1 |
| *Thioalkalivibrio* | 1 |
| *Thiolapillus* | 1 |
| *Endoriftia* | 1 |
| *Sedimenticola* | 1 |
| *Sulfuricaulis* | 1 |
| *Vogesella* | 1 |
| *Chitinimonas* | 1 |
| *Symbiobacter* | 1 |
| *Roseateles* | 1 |
| *Sphaerotilus* | 1 |
| *Duganella* | 1 |
| *Rhodocyclaceae* | 1 |
| *Zoogloeaceae* | 1 |
| *Actimicrobium* | 1 |
| *Methyloversatilis* | 1 |
| *Denitratisoma* | 1 |
| *Methylobacillus* | 1 |
| *Methylophilus* | 1 |
| *Sideroxydans* | 1 |
| *Gallionella* | 1 |
| *Ferriphaselus* | 1 |
| *Sideroxyarcus* | 1 |
| *Sulfuriferula* | 1 |
| *Oryzomicrobium* | 1 |
| *Niveibacterium* | 1 |
| *Quatrionicoccus* | 1 |
| *Ferribacterium* | 1 |
| *Azonexus* | 1 |
| *Nitrogeniibacter* | 1 |
| *Parazoarcus* | 1 |
| *Aquibium* | 1 |
| *Allorhizobium* | 1 |
| *Neorhizobium* | 1 |
| *Shinella* | 1 |
| *Georhizobium* | 1 |
| *Peteryoungia* | 1 |
| *Bosea* | 1 |
| *Pleomorphomonas* | 1 |
| *Hartmannibacter* | 1 |
| *Aureimonas* | 1 |
| *Jiella* | 1 |
| *Roseibium* | 1 |
| *Methyloraptor* | 1 |
| *Yoonia* | 1 |
| *Pararhodospirillum* | 1 |
| *Paramagnetospirillum* | 1 |
| *Sulfurimonas* | 1 |
| *Poseidonibacter* | 1 |
| *Malaciobacter* | 1 |
| *Desulfosarcina* | 1 |
| *Desulfoluna* | 1 |
| *Caulifigura* | 1 |
| *Gaopeijia* | 1 |
| *Denitrovibrio* | 1 |
| *Geovibrio* | 1 |
| *Mucispirillum* | 1 |
| *Pristimantibacillus* | 1 |
| *Capillimicrobium* | 1 |
| *Leptolyngbya* | 1 |
| *Vulcanimicrobium* | 1 |
