# Supplementary material for: A chemical bactericide dioctyldiethylenetriamine (Xinjunan) exerts a non-lethal effect by inhibiting RpfG activity to regulate the quorum sensing system
Source: PLoS Pathog. 2026 Jun 10;22(6):e1014320. doi: 10.1371/journal.ppat.1014320 (PMC13274925; doi:10.1371/journal.ppat.1014320)
Supplement: S9 Table — (DOCX) [file ppat.1014320.s024.docx]

**S9 Table.** Phylogenetic relationship of RpfG in 100 bacteria.

| **Strains** | **Protein id** | **Similarity (%)** |
| --- | --- | --- |
| *Xanthomonas oryzae* pv. *oryzae* (*Xoo*) | [ACD58392](https://www.ncbi.nlm.nih.gov/protein/ACD58392) | 100.00 |
| *Xanthomonas oryzae* pv*. oryzicola* (*Xoc*) | [AEQ96401](https://www.ncbi.nlm.nih.gov/protein/AEQ96401) | 92.59 |
| *Xanthomonas euvesicatoria* pv. *vesicatoria* (*Xcv*) | [CAJ23594](https://www.ncbi.nlm.nih.gov/protein/CAJ23594) | 92.33 |
| *Xanthomonas citri* pv. *fuscans* (*Xfu*) | [CDF61564](https://www.ncbi.nlm.nih.gov/protein/CDF61564) | 92.33 |
| *Xanthomonas perforans* (*Xpe*) | [APO98847](https://www.ncbi.nlm.nih.gov/protein/APO98847) | 92.33 |
| *Xanthomonas vesicatoria* (*Xve*) | [APP74038](https://www.ncbi.nlm.nih.gov/protein/APP74038) | 92.06 |
| *Xanthomonas hortorum* (*Xhr*) | [ASW45037](https://www.ncbi.nlm.nih.gov/protein/ASW45037) | 91.53 |
| *Xanthomonas fragariae* (*Xfr*) | [AOD14922](https://www.ncbi.nlm.nih.gov/protein/AOD14922) | 90.48 |
| *Xanthomonas campestris* pv. *raphani* (*Xcp*) | [AEL06994](https://www.ncbi.nlm.nih.gov/protein/AEL06994) | 90.74 |
| *Xanthomonas campestris* pv. *campestris* (*Xcc*) | [AAY49388](https://www.ncbi.nlm.nih.gov/protein/AAY49388) | 90.74 |
| *Stenotrophomonas maltophilia* (*Sm*) | AEM51210 | 80.47 |
| *Stenotrophomonas forensic* (*Sfoe*) | [WDM65727](https://www.ncbi.nlm.nih.gov/protein/WDM65727) | 80.21 |
| *Stenotrophomonas indicatrix* (*Sinc*) | [QBR44434](https://www.ncbi.nlm.nih.gov/protein/QBR44434) | 80.31 |
| *Stenotrophomonas rhizophila* (*Srh*) | [AOA72234](https://www.ncbi.nlm.nih.gov/protein/AOA72234) | 81.27 |
| *Pseudoxanthomonas spadix* (*Psd*) | [AER56548](https://www.ncbi.nlm.nih.gov/protein/AER56548) | 75.99 |
| *Pseudoxanthomonas winnipegensis* (*Pwi*) | [WJI17609](https://www.ncbi.nlm.nih.gov/protein/WJI17609) | 77.12 |
| *Pseudoxanthomonas daejeonensis* (*Pdd*) | [UNK57125](https://www.ncbi.nlm.nih.gov/protein/UNK57125) | 77.84 |
| *Lysobacter capsica* (*Lcp*) | [ALN85766](https://www.ncbi.nlm.nih.gov/protein/ALN85766) | 77.68 |
| *Lysobacter enzymogenes* (*Lez*) | [ALN57630](https://www.ncbi.nlm.nih.gov/protein/ALN57630) | 77.40 |
| *Lysobacter helvus* (*Lhx*) | [BCT97004](https://www.ncbi.nlm.nih.gov/protein/BCT97004) | 79.33 |
| *Stenotrophomonas acidaminiphila* (*Sacz*) | [ALJ28407](https://www.ncbi.nlm.nih.gov/protein/ALJ28407) | 80.17 |
| *Xylella fastidiosa* 9a5c (*Xfa*) | [AAF83923](https://www.ncbi.nlm.nih.gov/protein/AAF83923) | 73.83 |
| *Xylella fastidiosa* Hib4 (*Xfh*) | [ALR07127](https://www.ncbi.nlm.nih.gov/protein/ALR07127) | 74.48 |
| *Xylella fastidiosa* M12 (*Xfm*) | [ACA11469](https://www.ncbi.nlm.nih.gov/protein/ACA11469) | 73.70 |
| *Thiobacillus denitrificans* (*Tbd*) | [AAZ98623](https://www.ncbi.nlm.nih.gov/protein/AAZ98623) | 49.58 |
| Thiobacillus sediment (*Tsw*) | [WRS39134](https://www.ncbi.nlm.nih.gov/protein/WRS39134) | 46.15 |
| *Thioalkalivibrio sulfidiphilus* (*Tgr*) | [ACL71185](https://www.ncbi.nlm.nih.gov/protein/ACL71185) | 43.81 |
| *Thioflavicoccus mobilis* (*Tmb*) | [AGA90467](https://www.ncbi.nlm.nih.gov/protein/AGA90467) | 44.44 |
| *Sulfuricaulis limicola* (*Slim*) | [BAV33778](https://www.ncbi.nlm.nih.gov/protein/BAV33778) | 48.44 |
| *Candidatus Endoriftia persephone* (*Eps*) | [USF86159](https://www.ncbi.nlm.nih.gov/protein/USF86159) | 48.45 |
| *Sedimenticola thiotaurini* (*Seds*) | [AKH19330](https://www.ncbi.nlm.nih.gov/protein/AKH19330) | 48.22 |
| *Methylophaga frappieri* (*Mec*) | [AFJ02594](https://www.ncbi.nlm.nih.gov/protein/AFJ02594) | 42.06 |
| *Methylophaga marina* (*Mmaf*) | [BDZ74361](https://www.ncbi.nlm.nih.gov/protein/BDZ74361) | 43.50 |
| *Thiolapillus brandeum* (*Tbn*) | [BAO43978](https://www.ncbi.nlm.nih.gov/protein/BAO43978) | 41.99 |
| *Leptospirillum ferrooxidans* (*Lfc*) | [BAM07982](https://www.ncbi.nlm.nih.gov/protein/BAM07982) | 45.23 |
| *Sideroxydans lithotrophicus* (*Slt*) | [ADE11527](https://www.ncbi.nlm.nih.gov/protein/ADE11527) | 43.24 |
| *Methylotenera versatilis* (*Meh*) | [ADI30829](https://www.ncbi.nlm.nih.gov/protein/ADI30829) | 43.61 |
| *Methylobacillus flagellates* (*Mfa*) | [ABE50918](https://www.ncbi.nlm.nih.gov/protein/ABE50918) | 46.11 |
| *Sulfuriferula plumbiphila* (*Splb*) | [BBP03788](https://www.ncbi.nlm.nih.gov/protein/BBP03788) | 45.83 |
| *Herbaspirillum rubrisubalbicans* (*Hrb*) | [ALU88265](https://www.ncbi.nlm.nih.gov/protein/ALU88265) | 40.23 |
| *Herbaspirillum frisingense* (*Hfr*) | [QNB06415](https://www.ncbi.nlm.nih.gov/protein/QNB06415) | 40.23 |
| *Herbaspirillum seropedicae* (*Hse*) | [ADJ62749](https://www.ncbi.nlm.nih.gov/protein/ADJ62749) | 40.40 |
| *Candidatus methylospira mobilis* (*Mmob*) | [QFY42381](https://www.ncbi.nlm.nih.gov/protein/QFY42381) | 44.76 |
| *Zoogloeaceae bacterium* (*Zpa*) | [AVZ78157](https://www.ncbi.nlm.nih.gov/protein/AVZ78157) | 44.76 |
| *Aquitalea magnusonii* (*Amah*) | [BBF86314](https://www.ncbi.nlm.nih.gov/protein/BBF86314) | 44.63 |
| *Duganella zoogloeoides* (*Dzo*) | [WQH02697](https://www.ncbi.nlm.nih.gov/protein/WQH02697) | 42.21 |
| *Ferriphaselus Amnicola* (*Fam*) | [BBE50308](https://www.ncbi.nlm.nih.gov/protein/BBE50308) | 44.19 |
| *Paucibacter sediminis* (*Pais*) | [WIT09748](https://www.ncbi.nlm.nih.gov/protein/WIT09748) | 43.34 |
| Sphaerotilus sulfidivorans (*Snn*) | [QEN02323](https://www.ncbi.nlm.nih.gov/protein/QEN02323) | 42.15 |
| *Undibacterium cyanobacteriorum* (*Ucy*) | [WMW81614](https://www.ncbi.nlm.nih.gov/protein/WMW81614) | 43.85 |
| *Rhodoferax antarcticus* (*Rac*) | [APW46264](https://www.ncbi.nlm.nih.gov/protein/APW46264) | 39.83 |
| *Rhodoferax mekongensis* (*Rmk*) | [WNO03344](https://www.ncbi.nlm.nih.gov/protein/WNO03344) | 41.62 |
| *Massilia violaceinigra* (*Mass*) | [ATQ75026](https://www.ncbi.nlm.nih.gov/protein/ATQ75026) | 44.66 |
| *Oryzomicrobium terrae* (*Otr*) | [QEL64830](https://www.ncbi.nlm.nih.gov/protein/QEL64830) | 42.70 |
| *Niveibacterium microcysteis* (*Niv*) | [QSI76180](https://www.ncbi.nlm.nih.gov/protein/QSI76180) | 41.36 |
| *Nitrogeniibacter mangrove* (*Aaq*) | [QID18951](https://www.ncbi.nlm.nih.gov/protein/QID18951) | 45.04 |
| *Thauera aminoaromatica* (*Tmz*) | [ACK53942](https://www.ncbi.nlm.nih.gov/protein/ACK53942) | 44.08 |
| *Aromatoleum petrolei* (*Apet*) | [QTQ37962](https://www.ncbi.nlm.nih.gov/protein/QTQ37962) | 42.78 |
| *Azoarcus olearius* (*Azo*) | [CAL96302](https://www.ncbi.nlm.nih.gov/protein/CAL96302) | 42.59 |
| *Azospira restricta* (*Ares*) | [QRJ62225](https://www.ncbi.nlm.nih.gov/protein/QRJ62225) | 44..76 |
| *Dechloromonas aquae* (*Daj*) | [WVT85259](https://www.ncbi.nlm.nih.gov/protein/WVT85259) | 41.93 |
| *Dechloromonas denitrificans* (*Dden*) | [UCV06478](https://www.ncbi.nlm.nih.gov/protein/UCV06478) | 42.78 |
| *Dechloromonas aromatica* (*Dar*) | [AAZ47632](https://www.ncbi.nlm.nih.gov/protein/AAZ47632) | 44.19 |
| *Ferribacterium limneticum* (*Fle*) | [UCV18166](https://www.ncbi.nlm.nih.gov/protein/UCV18166) | 44.19 |
| *Chitinimonas koreensis* (*Cks*) | [QNM96253](https://www.ncbi.nlm.nih.gov/protein/QNM96253) | 43.63 |
| *Janthinobacterium tructae* (*Jas*) | [QDG69549](https://www.ncbi.nlm.nih.gov/protein/QDG69549) | 42.82 |
| *Azospirillum baldaniorum* (*Abs*) | [CCD01617](https://www.ncbi.nlm.nih.gov/protein/CCD01617) | 43.58 |
| *Azospirillum thermophilum* (*Azz*) | [AWK85749](https://www.ncbi.nlm.nih.gov/protein/AWK85749) | 38.59 |
| *Azospirillum humicireducens* (*Ahu*) | [AWB08696](https://www.ncbi.nlm.nih.gov/protein/AWB08696) | 40.73 |
| *Azospirillum thiophilum* (*Ati*) | [ALG74092](https://www.ncbi.nlm.nih.gov/protein/ALG74092) | 39.89 |
| *Vulcanimicrobium alpinum* (*Vab*) | [BDE05064](https://www.ncbi.nlm.nih.gov/protein/BDE05064) | 41.01 |
| *Methylorubrum populi* (*Mpo*) | [ACB82021](https://www.ncbi.nlm.nih.gov/protein/ACB82021) | 40.96 |
| *Methylobacterium durans* (*Mets*) | [AWN39612](https://www.ncbi.nlm.nih.gov/protein/AWN39612) | 43.10 |
| *Methylobacterium mesophilicum* (*Mmes*) | [QGY04590](https://www.ncbi.nlm.nih.gov/protein/QGY04590) | 37.11 |
| *Methylobacterium organophilum* (*Mog*) | [UMY18944](https://www.ncbi.nlm.nih.gov/protein/UMY18944) | 41.13 |
| *Phreatobacter cathodiphilus* (*Phr*) | [AVO44440](https://www.ncbi.nlm.nih.gov/protein/AVO44440) | 41.18 |
| *Phreatobacter aquaticus* (*Paqt*) | [QCK85241](https://www.ncbi.nlm.nih.gov/protein/QCK85241) | 43.91 |
| *Mesorhizobium jarvisii* (*Mjr*) | [QKC63278](https://www.ncbi.nlm.nih.gov/protein/QKC63278) | 39.39 |
| *Roseibium aggregatum* (*Lagg*) | [AQQ05820](https://www.ncbi.nlm.nih.gov/protein/AQQ05820) | 37.74 |
| *Peteryoungia desertarenae* (*Pdes*) | [QLF69257](https://www.ncbi.nlm.nih.gov/protein/QLF69257) | 35.16 |
| *Bradyrhizobium diazoefficiens* (*Bja*) | [BAC47605](https://www.ncbi.nlm.nih.gov/protein/BAC47605) | 40.79 |
| *Bradyrhizobium japonicum* (*Bju*) | [BAL12747](https://www.ncbi.nlm.nih.gov/protein/BAL12747) | 40.00 |
| *Bradyrhizobium ottawaense* (*Bot*) | [AWL91097](https://www.ncbi.nlm.nih.gov/protein/AWL91097) | 41.71 |
| *Bradyrhizobium vignae* (*Bvz*) | [SPP97772](https://www.ncbi.nlm.nih.gov/protein/SPP97772) | 40.96 |
| *Bradyrhizobium guangdongense* (*Bgq*) | [QAU41363](https://www.ncbi.nlm.nih.gov/protein/QAU41363) | 43.73 |
| *Rhodopseudomonas palustris* (*Rpa*) | [WCL90268](https://www.ncbi.nlm.nih.gov/protein/WCL90268) | 39.38 |
| *Yoonia vestfoldensis* (*Lvs*) | [ARU00105](https://www.ncbi.nlm.nih.gov/protein/ARU00105) | 42.49 |
| *Rhizobium johnstonii* (*Rle*) | [CAK09100](https://www.ncbi.nlm.nih.gov/protein/CAK09100) | 41.85 |
| *Rhizobium indicum* (*Rii*) | [QKK18411](https://www.ncbi.nlm.nih.gov/protein/QKK18411) | 42.42 |
| *Agrobacterium tumefaciens* (*At*) | [ADY65450](https://www.ncbi.nlm.nih.gov/protein/ADY65450) | 38.52 |
| *Jiella pelagia* (*Jie*) | [WAP70779](https://www.ncbi.nlm.nih.gov/protein/WAP70779) | 41.64 |
| *Pelagibacterium halotolerans* (*Phl*) | [AEQ53653](https://www.ncbi.nlm.nih.gov/protein/AEQ53653) | 41.90 |
| *Pelagibacterium flavum* (*Pem*) | [UYQ72056](https://www.ncbi.nlm.nih.gov/protein/UYQ72056) | 41.64 |
| *Devosia rhizosphaerae* (*Dri*) | [WIJ24770](https://www.ncbi.nlm.nih.gov/protein/WIJ24770) | 43.63 |
| *Devosia ginsengisoli* (*Dea*) | [QDZ13064](https://www.ncbi.nlm.nih.gov/protein/QDZ13064) | 41.93 |
| *Devosia oryziradicis* (*Doy*) | [QQR36006](https://www.ncbi.nlm.nih.gov/protein/QQR36006) | 42.49 |
| *Arcobacter cloacae* (*Aclo*) | [QKF90222](https://www.ncbi.nlm.nih.gov/protein/QKF90222) | 39.15 |
| *Poseidonibacter lekithochrous* (*Alk*) | [QKJ22571](https://www.ncbi.nlm.nih.gov/protein/QKJ22571) | 36.83 |
| *Mucispirillum schaedleri* (*Msch*) | [USF23661](https://www.ncbi.nlm.nih.gov/protein/USF23661) | 33.14 |
| *Thiomicrorhabdus lithotrophica* (*Tlh*) | [WEJ62602](https://www.ncbi.nlm.nih.gov/protein/WEJ62602) | 26.35 |
